# A case study of proton shuttling in palladium catalysis†
†Electronic supplementary information (ESI) available: Experimental details and characterization data; computational studies. See DOI: 10.1039/c5sc04232a


**DOI:** 10.1039/c5sc04232a

**Published:** 2015-12-07

**Authors:** Julien Monot, Paul Brunel, Christos E. Kefalidis, Noel Ángel Espinosa-Jalapa, Laurent Maron, Blanca Martin-Vaca, Didier Bourissou

**Affiliations:** a Université de Toulouse , UPS , 118 route de Narbonne , F-31062 Toulouse , France; b CNRS , LHFA UMR5069 , F-31062 Toulouse , France . Email: bmv@chimie.ups-tlse.fr ; Email: dbouriss@chimie.ups-tlse.fr; c Université de Toulouse , INSA , UPS , LCPNO , CNRS , UMR 5215 CNRS-UPS-INSA , 135 avenue de Rangueil , 31400 Toulouse , France . Email: laurent.maron@irsamc.ups-tlse.fr

## Abstract

The mechanism of alkynoic acid cycloisomerization with SCS indenediide Pd pincer complexes has been investigated experimentally and computationally. These studies confirmed the cooperation between the Pd center and the backbone of the pincer ligand, and revealed the involvement of a second molecule of substrate. It acts as a proton shuttle in the activation of the acid, it directs the nucleophilic attack of the carboxylic acid on the π-coordinated alkyne and it relays the protonolysis of the resulting vinyl Pd species. A variety of H-bond donors have been evaluated as external additives, and polyols featuring proximal hydroxyl groups, in particular catechol derivatives, led to significant catalytic enhancement. The impact of 4-nitrocatechol and 1,2,3-benzenetriol is particularly striking on challenging substrates such as internal 4- and 5-alkynoic acids. Endo/exo selectivities up to 7.3/1 and 60-fold increase in reactivity were achieved.

## Abstract 2

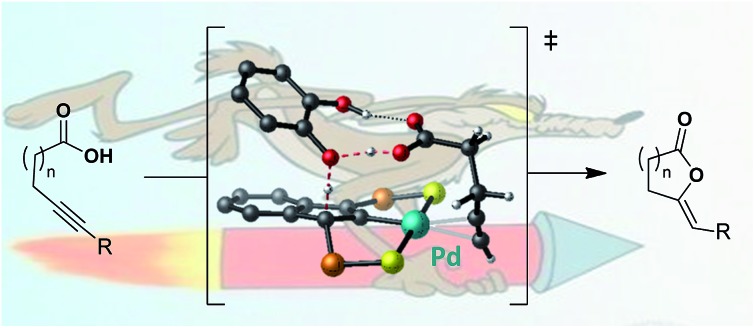
Thanks to mechanistic studies, the catalytic performance of SCS indenediide Pd pincer complexes has been spectacularly enhanced using catechol additives as proton shuttles.

## Introduction

The interplay between experiment and theory is extremely powerful for the study of the mechanism of catalytic transformations, to understand the factors influencing their efficiency and selectivity, and finally to optimize their performance.^1^ From an experimental point of view, mechanistic proposals are typically drawn on the basis of kinetic studies,^2^ isotopic labeling experiments^3^ and characterizations of intermediates.^4^ On the other hand, the advent of DFT methods has revolutionized the ability of computational methods to describe chemical systems and nowadays virtually all molecular compounds, including transition metal complexes, can be investigated *in extenso*. Accordingly, accurate and valuable information can be gained on the entire reaction profile. Geometric and electronic structures of key intermediates and transition states can be analyzed, different mechanistic scenarios can be probed and compared, kinetic data and selectivities can be estimated.^5^ Combining and comparing the experimental and theoretical data is a major source of information and often helps to identify and understand the very mechanism of catalytic transformations.

**Chart 1:**
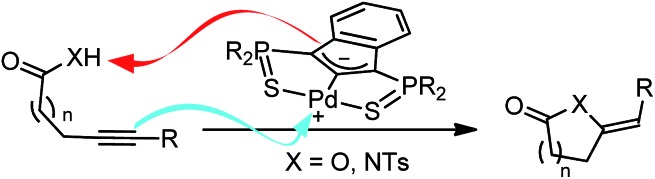
Cycloisomerization of alkynoic acids and *N*-tosylalkynylamides promoted by palladium indenediide pincer complexes.

In light of our activities on pincer complexes, we recently reported SCS indenediide Pd complexes featuring ligand non-innocent character.^6^ These indenediide complexes proved very efficient in the catalytic cycloisomerization of alkynoic acids as well as *N*-tosylalkynylamides (Chart 1).^7^ They do not require the presence of an external base and represent a rare example of metal–ligand cooperative catalysis involving Pd.^8^ The catalytic system can be recycled without deactivation up to ten times and it is efficient for a large variety of substrates, giving access to 5-, 6- and even 7-membered alkylidene lactones and lactams with high selectivity (generally in favor of the exo product). The SCS indenediide Pd complexes have clearly enhanced the efficiency and extended the scope of such cycloisomerizations, but there is certainly room for further improvement. In particular, the cyclization of substrates featuring internal alkynes remains challenging. It requires much higher temperatures and longer reaction times, and the exo/endo selectivity is often modest.

Concerning the mechanism of cycloisomerization, first insights have been obtained experimentally.^7^ The active participation of the SCS pincer ligand to the activation of substrate has been substantiated (non-innocent character by protonation of electron-rich indenediide backbone). In addition, analysis of the stereochemical outcome of the reaction (with internal alkynes or *via* D-labeling of terminal alkynes) indicated the exclusive formation of *Z* products, in line with *anti* addition of the carboxylic acid/amide to the Pd-coordinated alkyne. But the exact ways the substrate is activated and the cyclization proceeds are still to be determined.

These two aspects (precise understanding of the mode of action of the Pd indenediide pincer complexes and further catalytic improvement) prompted us to perform detailed investigations combining theory and experiment. Here, we report DFT calculations and kinetic studies that shed light onto the mechanism of the cycloisomerization. The reaction is proposed to involve a second molecule of alkynoic acid acting as a proton shuttle, and this paved the way for catalytic enhancement. A broad variety of H-bond donor additives have been screened and catechol derivatives were found to improve very significantly both activity and selectivity. The combination of Pd-ligand cooperative catalysis and proton shuttling was successfully applied to the cycloisomerization of internal 4- and 5-alkynoic acids.

## Results and discussion

### DFT studies

The mode of action of the Pd indenediide pincer complex in the cycloisomerization of alkynoic acids was first investigated computationally using DFT theory (B3PW91 functional).^9^ Calculations were performed on the real Pd system without simplification (phenyl groups were introduced at the P atoms, as in the first reported complexes7*a*). A mononuclear T-shape SCS complex with a vacant coordination site was considered as starting species since the nature of the Pd pre-catalyst (co-ligand at Pd and mono *vs.* polynuclear character) was found to only marginally influence catalytic performance.^7^

**Fig. 1:**
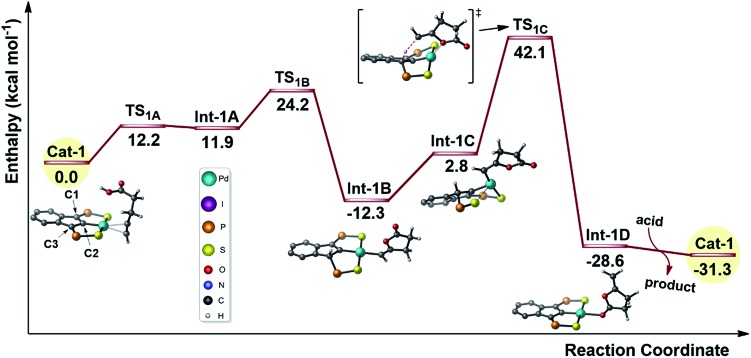
Reaction profile (kcal mol^–1^) computed for the cyclization of 4-pentynoic acid involving one molecule of substrate per Pd center. The phenyl substituents on the P atoms are omitted for clarity.

The reaction profile for the cyclization of 4-pentynoic acid involving one molecule of substrate per Pd center was explored first (Fig. 1). The “active catalyst” **Cat-1** is formed by side-on coordination of the alkyne to Pd. The carboxylic acid is readily activated (with a barrier of only 12.2 kcal mol^–1^) and the proton is transferred to the indene backbone. Cyclization then occurs by nucleophilic attack of one oxygen atom to the alkyne affording **Int-1B** (the corresponding transition state **TS_1B_** lies 24.2 kcal mol^–1^ above **Cat-1**). The catalytic cycle is closed by protonolysis of the vinyl-Pd species (back transfer of the proton from the indene backbone *via***TS_1C_**) and substrate/product exchange at Pd. This reaction profile follows the mechanistic scenario commonly proposed to account for the metal-catalyzed cycloisomerization of alkynoic acids,^10^ but two inconsistencies makes it very unlikely. First, the cyclization occurs by *syn* addition to the π-coordinated alkyne (O attacks *cis* to Pd) while *anti* addition is observed experimentally (as shown by D-labeling experiments).^11^ Second, the activation barrier for the proton back-transfer leading to **Int-1D** (54.4 kcal mol^–1^ from **Int-1B**) is prohibitively high.

**Fig. 2:**
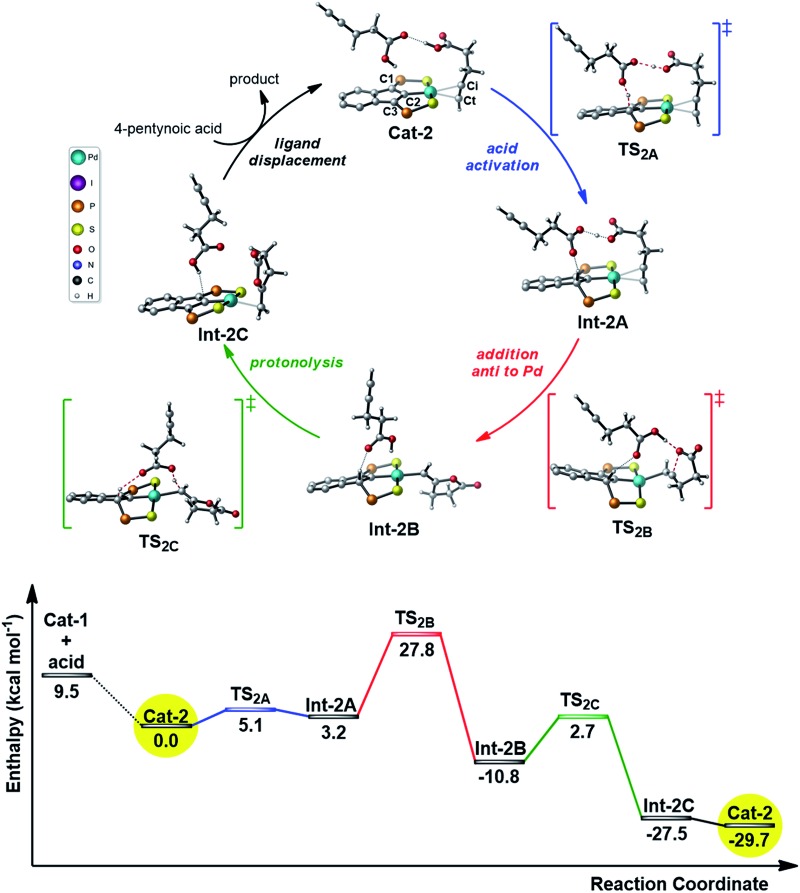
Reaction profile (kcal mol^–1^) computed for the cyclization of 4-pentynoic acid involving two molecules of substrate per Pd center. The phenyl substituents on the P atoms are omitted for clarity, the key carbon atoms of the indene backbone are labelled C1/C2/C3 and the carbons atoms of the alkynyl substrate are labelled Ct/Ci.

Given the results of the kinetic studies (*vide infra*), we envisioned to refine our model by involving a second molecule of alkynoic acid in the cycloisomerization process. Interaction of **Cat-1** with 4-pentynoic acid is downhill in energy and leads to **Cat-2** (Fig. 2). The alkyne bonded to Pd is oriented perpendicular to the coordination plane and its carboxylic acid moiety is engaged in H-bonding. The second molecule of substrate approaches perpendicularly and its acidic proton lies in close proximity to the indene backbone. Accordingly, activation of the acid moiety is very easy (activation barrier of only 5.1 kcal mol^–1^) and involves the “external” substrate as a proton shuttle between the π-bonded alkynoic acid and the electron-rich carbon atom C3 (transition state **TS_2A_**). The formation of **Int-2A** substantiates the non-innocent character of the indenediide backbone which is temporarily protonated. Cyclization then occurs by nucleophilic attack of one oxygen atom to the internal carbon atom of the alkyne (Ci). The reaction is directed by an “external” substrate molecule which remains H-bonded during the whole process. It involves the rotation of the alkyne and its slippage (in **TS_2B_**, it is essentially σ-bonded *via* the terminal carbon atom Ct). The cyclization requires important geometric changes, but it now proceeds *via anti* addition (in the resulting vinyl Pd complex **Int-2B**, the O atom is *trans* to Pd) and the corresponding activation barrier is accessible (27.8 kcal mol^–1^ from **Cat-2**). The external substrate molecule weakly interacts with the proton at C3 in **Int-2B**, and subsequently relays the protonolysis of the vinyl Pd species. The formation of the alkylidene lactone is strongly exothermic (**Int-2C** lies 27.5 kcal mol^–1^ below **Cat-2**), and thanks to this proton shuttling, the associated activation barrier is readily accessible (only 13.5 kcal mol^–1^). Product to substrate exchange at Pd closes the cycle and regenerates **Cat-2**.

Thus, involving two molecules of 4-pentynoic acid, it is possible to propose a catalytic cycle which is consistent with the experimental observations. The second molecule of substrate acts as a proton shuttle in the acid activation and in the protonolysis of the key vinyl Pd intermediate. It also directs the cyclization to *anti* addition.

### Kinetic studies

**Fig. 3:**
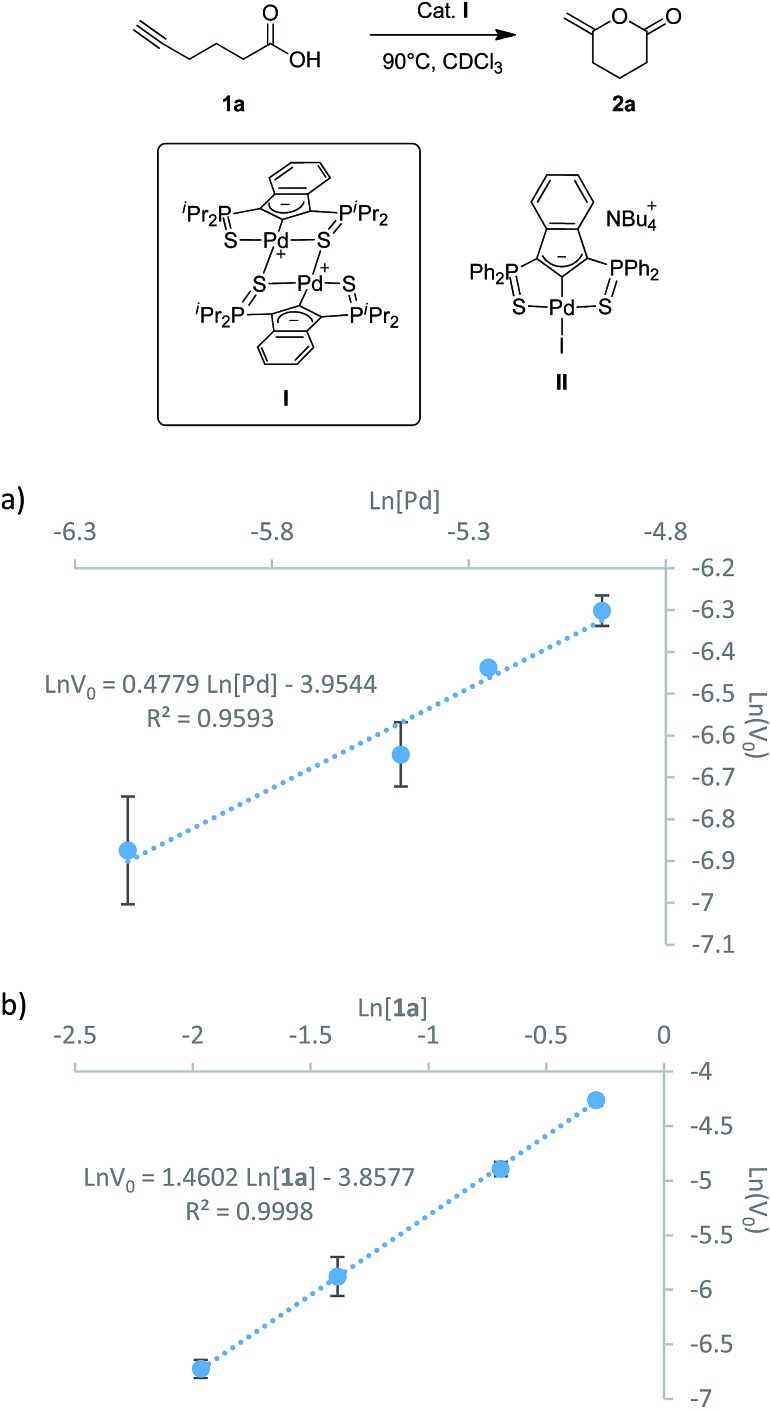
Dependence of the initial rate of 5-hexynoic acid cyclization on the concentration of palladium (a) and substrate (b). The rates were averaged over three independent measurements. The error bars represent the standard deviation of the results from the three independent measurements.

In parallel with the DFT calculations, kinetic studies were performed. The partial orders in palladium and substrate were determined using the initial rate method. The iPr-substituted palladium dimer **I** (Fig. 3) was chosen as pre-catalyst (rather than the Ph-substituted iodo/chloropalladate complexes or the corresponding neutral trimer) because of practical reasons (better solubility and stability, absence of ammonium counter-cations).7*b* The cyclization of 5-hexynoic acid **1a** was selected as model reaction and it proceeded within hours at 90 °C in deuterated chloroform and could thus be conveniently monitored by NMR using a pressure tube. Under these conditions, the Pd precatalyst and substrate are fully soluble and no induction period is observed. ^1^H NMR monitoring shows the concomitant consumption of **1a** and formation of methylene-valerolactone **2a**, and no intermediate or side-product is detected. The initial rates were determined at different Pd loadings (3, 5, 7.5, 10 mol% in [Pd], Fig. S1†) and at different substrate concentrations (0.14–0.75 M, Fig. S4†). The initial rates of the cycloisomerization reaction were determined by plotting the evolution of the methylene-valerolactone **2a** concentration *versus* reaction time at the various concentrations of catalyst or substrate (Fig. S2 and S5†). Each experiment was run in triplicate for a better accuracy and the averaged values were linearly fitted to equations ln *v*_0_ = *a* ln[Pd] + *b* (Fig. 3a) and ln *v*_0_ = *c* ln[**1a**] + *d* (Fig. 3b) for palladium and 5-hexynoic acid variations, respectively. Accordingly, the rate law can be expressed as follows: *v* = *k*[Pd]^1/2^[Sub]^3/2^.

The partial order of 0.5 in [Pd] clearly indicates that the dinuclear complex **I** is not the active species. It is in fact the resting state of the precatalyst, which dissociates and coordinates the substrate to give the active species. The dinuclear form is favored at room temperature (the SCS pincer ligand is dissymmetric and two distinct signals are observed by ^31^P NMR), but tends to dissociate upon heating (the ^31^P NMR signals progressively broaden and coalesce at *ca.* 90 °C, Fig. S24†). Similar situations (square root order in [Pd] and off-cycle equilibrium of the active mononuclear species with an inactive dimeric form) have been encountered in Pd-catalyzed Heck couplings and C–H acetoxylation of benzene.^12^

The partial order of 1.5 in [**1a**] also deserves comment. It is unexpected and reveals that the reaction is not a simple unimolecular process, as may be anticipated for a cycloisomerization. The way the substrate reacts is apparently more complicated and in line with the reaction profile computed by DFT, and it is proposed that a second molecule of alkynoic acid is involved in the catalytic cycle. The partial order in [**1a**] determined experimentally at 90 °C is not 2 but 1.5, probably due to the tendency of carboxylic acids to form dimers in solution.^13^ This self-association phenomenon tends to reduce the partial order in substrate. It has been tracked by IR and a dimer to monomer ratio of 7.5 has been determined for **1a** at 25 °C and 0.14 mol L^–1^.^14^

According to calculations, the second molecule of substrate participates *via* H-bonding and acts as a proton shuttle. We were thus intrigued about the possibility to use H-bond donors as additives in these cycloisomerization processes. These additive could substitute the “external” molecule of substrate, and optimizing the structure of the additive may afford the opportunity to enhance the catalytic performance of the Pd complex in terms of both activity and selectivity.

### Screening of H-bond donor additives

**Scheme 1:**
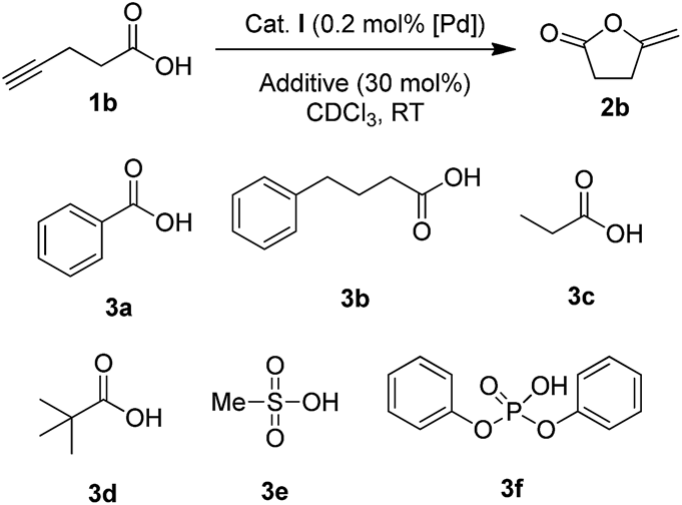
Additives having no impact (**3a–d**) or inhibiting (**3e,f**) the cyclization of 4-pentynoic acid **1b**.

The influence of additives was first investigated on 4-pentynoic acid **1b**, since its cyclization is easier to perform (it proceeds within hours at rt with complex **I**). We started the screening with aliphatic and aromatic carboxylic acids **3a–d** (30 mol%) (Scheme 1), but in all cases, the conversion of **1b** was essentially unaffected (∼75% after 1 h, ∼86% after 2 h, Table S9†).^15^ Stronger acids such as methane sulfonic acid **3e** and diphenylphosphoric acid **3f** completely inhibited the reaction, probably due to the protonation of the indenediide backbone, giving inactive indenyl species.

**Fig. 4:**
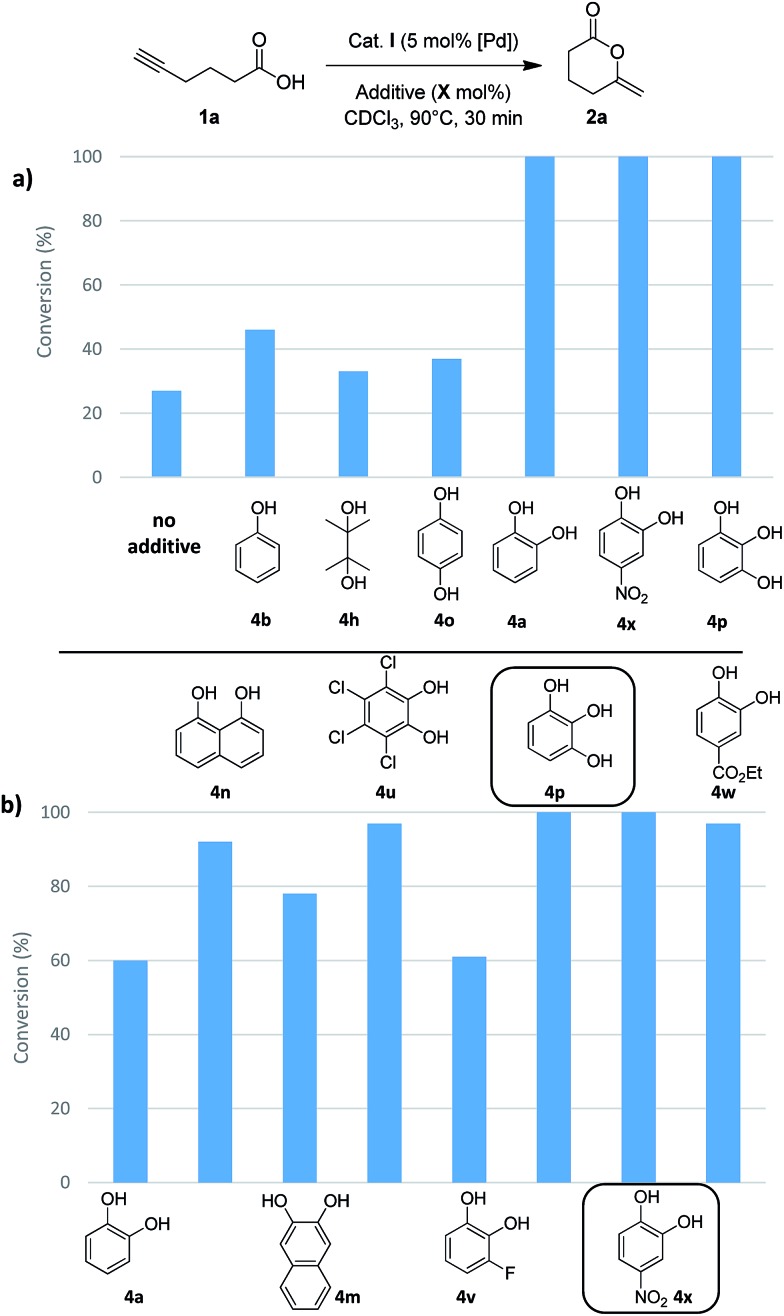
Impact of H-donor additives on the cyclization of 5-hexynoic acid **1a**. The reaction is performed with 30 or 10 mol% of additives (a and b, respectively) and the conversion of **1a** after 30 min is reported.

Then, we turned our attention to alcohols, starting with catechol (**4a**) which has already been used as additive in organo-catalyzed aldol reactions.^16^ The addition of 30 mol% of **4a** significantly speeds up the cycloisomerization of **1b** (complete conversion required only 30 min instead of 5 h without additive). To confirm the impact of **4a**, it was then tested on the cyclization of 5-hexynoic acid **1a** (which is much less reactive than **1b**), and again the reaction time was significantly reduced (from 10 h to 30 min to achieve complete conversion at 90 °C). These encouraging results prompted us to screen a broad variety of alcohols, diols and triols with aliphatic and aromatic skeletons. Overall, 24 compounds (**4a–x**) were tested on the cyclization of 5-hexynoic acid **1a**. Selected results are displayed in Fig. 4 (see Fig. S26 and S27† for more details).

**Scheme 2:**
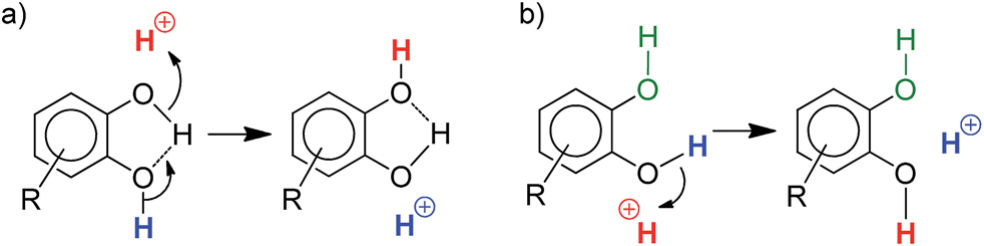
Schematic representation of two different modes of proton transfer with catechol derivatives.

Simple alcohols have little or no impact, but diols and triols were found to significantly improve the conversion, in particular the aromatic ones.^17^ After 30 min, the conversion of **1a** is only of 27% when the Pd complex **I** is used alone, but it is complete when 8 additives are adjoined: catechol **4a**, its derivatives substituted with electron-withdrawing groups (**4u**, **4v**, **4w** and **4x**), 1,2,3-benzenetriol **4p**, 1,8-dihydroxynaphtalene **4m** and 2,3-dihydroxynaphthalene **4n**. The most efficient additives are those featuring proximal hydroxyl groups, to ensure optimal proton transfer. This is particularly apparent when comparing hydroquinone **4o** (which has only little effect) and catechol.^18^ The proton transfer may involve the two hydroxyl groups (proton shuttling *via* H-bonding, Scheme 2a) or only one (Scheme 2b, the other hydroxyl group can participate in adjacent H-bonding).

In order to discriminate the additives enabling complete conversion of **1a** after 30 min, their loading was reduced, first to 10 mol% (Fig. 4b) and then to 5 mol% (Table S10†). Under these conditions, conversions of 90% or more were also achieved. The best results were obtained with 4-nitrocatechol **4x** which gave full conversion of **1a** within 30 min, even at 5 mol%. With this leading additive, we sought to optimize the reaction conditions (Table 1). Using 5 mol% of Pd and 5 mol% of **4x**, the reaction temperature could be significantly decreased (entries 2 and 3): at 60 °C, 5-hexynoic acid **1a** was fully consumed in 30 min, and even at 40 °C, full conversion was reached in only 1 h, while 10 h of reaction at 90 °C are needed without additive. The catalyst loading could also be significantly reduced. Using 5 mol% of 4-nitrocatechol **4x** and only 1 mol% of Pd, **1a** was completely cycloisomerized within 30 min at 90 °C (entry 4). Full conversion could also be achieved with only 0.2 mol% of Pd and 1 mol% of 4-nitrocatechol **4x** after 36 h at 90 °C (entry 5).

**Table 1:** Optimization of the reaction conditions for the cyclization of 5-hexynoic acid **1a** catalyzed by complex **I**, with 4-nitrocatechol **4x** as additive

Entry^*a*^	Mol% Pd	Mol% additive	*T* (°C)	Time	Conversion^*b*^
1	5	5	90	30 min	>99%
2	5	5	60	30 min	>99%
3	5	5	40	1 h	>99%
4	1	5	90	40 min	>99%
5	0.2	1	90	36 h	>99%

^*a*^Catalytic reactions performed under argon using 0.1 mmol of **1a** (0.14 M in CDCl_3_) and dimer **I** (5 mol% [Pd]).

^*b*^Conversion were determined by ^1^H NMR analysis.

The impact of the additive on the kinetics of the reaction, and in particular on the partial order in substrate, was then explored. Tetrachlorocatechol **4u** was chosen as the additive for solubility issues, and its loading was varied (catechol to Pd ratio of 0.2, 1, 2 and 4). The reactions were carried out at 25 °C to ensure accurate NMR monitoring.^9^ A strong impact of the catechol was observed, with a significant decrease of the partial order in substrate (from 1.33 without additive, to 0.62 for a catechol to Pd ratio of 0.2, and even to 0.26 for a catechol to Pd ratio of 4). Self-association of the carboxylic acid as well as crossed association with the catechol probably comes into play here and it is not surprising that fractional orders below 1 are obtained.

**Fig. 5:**
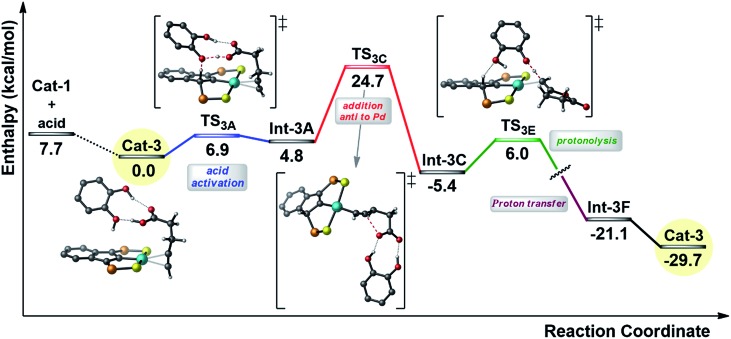
Simplified reaction profile (kcal mol^–1^) computed for the cyclization of 4-pentynoic acid in the presence of catechol. The phenyl substituents on the P atoms are omitted for clarity.

In order to gain insight into the effect of the additive, the reaction profile for the cyclization of 4-pentynoic acid in the presence of catechol (chosen for symmetry reasons) was computed at the DFT level (Fig. 5).

As for the mechanism involving two carboxylic acid molecules, the first step corresponds to the activation of the carboxylic acid with protonation of the indenyl backbone (transition state **TS_3A_**). In this case, it is assisted by the catechol molecule. One hydroxyl group acts as a relay for the proton transfer (from the substrate to the indenyl) while the second hydroxyl is involved in hydrogen bonding with the C=O group of the carboxylic acid (*cf.*Scheme 2b). Then, the cyclization occurs *via anti*-attack of the carboxylate, which is engaged in double hydrogen bonding with the catechol (**TS_3C_**). Finally, the proton borrowed by the indenyl backbone is transferred back and the alkylidene lactone is released. In this case, this is a two-step process, with protonation of the vinyl Pd species by catechol and then proton transfer from the indenyl to the catechol (see ESI†). The catechol acts here a proton shuttle (see Scheme 2a), in a similar way to the “external” molecule of carboxylic acid. Note that the rate-determining step is again the cyclization and that the corresponding activation barrier is about 3 kcal mol^–1^ lower in energy than that computed for the mechanism involving two carboxylic acid molecules, in line with the rate improvement observed experimentally.

### Cycloisomerization of internal alkynoic acids in the presence of catechol additives

The cyclization of alkynoic acids bearing internal alkynes is notoriously challenging. These substrates are much less reactive than terminal alkynoic acids, and exo/endo selectivity issues are frequently met.^11^,^19^,^20^ We were thus very eager to assess the influence of catechol additives in such difficult cycloisomerizations. Catalytic runs were performed at 90 °C with 5 mol% of Pd (dimer **I**) and 30 mol% of additive (either 4-nitrocatechol **4x** or 1,2,3-benzenetriol **4p**) on four representative substrates (Table 2).

**Table 2:** Influence of catechol additives on the cycloisomerization of alkynoic acids bearing internal alkynes

Entry^*a*^	Alkynoic acid	Lactone	Additive	Time	Conv^*b*^ (%)	Exo/endo
1	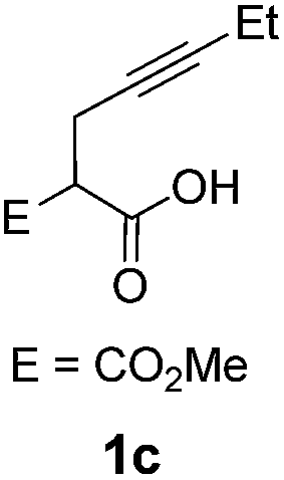	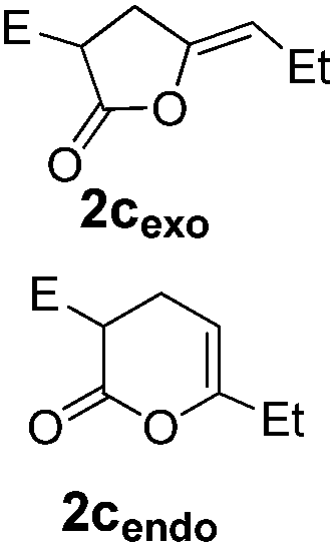	—	1.5 h	99	1/1.2
			**4x** (30%)	10 min	99	1/5.3
			**4p** (30%)	10 min	99	1/7.3
2	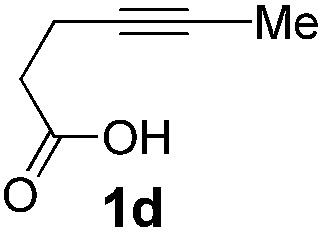	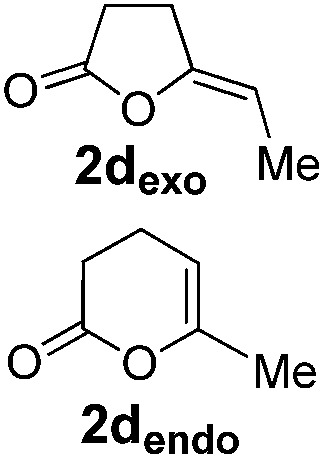	—	28 h	98	1.5/1
			**4x** (30%)	30 min	99	1/1.5
			**4p** (30%)	30 min	99	1/2.3
3	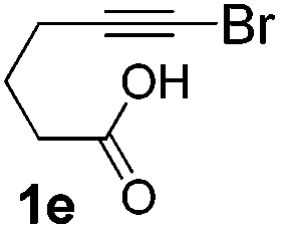	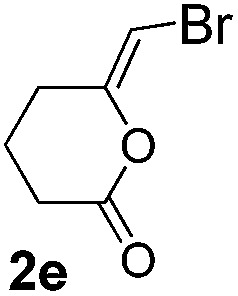	—	5 h	99	1/0
			**4x** (5%)	30 min	99	1/0
4	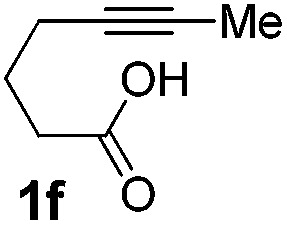	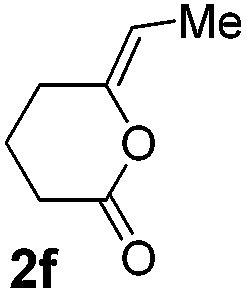	—	23 h	n.r.	1/0
			**4x** (30%)	60 h	99	1/0

^*a*^Catalytic reactions performed at 90 °C under argon atmosphere using 0.1 mmol of the corresponding alkynoic acid **1a–h** (0.1 M in CDCl_3_) and dimer **I** (5 mol% [Pd]).

^*b*^Conversions were determined by ^1^H NMR analysis.

Pd complexes have rarely been used to catalyze the cycloisomerization of internal 4-alkynoic acids.19a,b Compounds **1c** and **1d** were tested and in both cases, the additives spectacularly shortened the reaction time (entries 1 and 2). The cyclization of **1c** could be completed within 10 min (instead of 1.5 h) and that of **1d** required only 30 min (instead of 28 h), representing speeds up by approximately 10 to 60 times. The additives also noticeably influenced the regioselectivity of the cyclization and favored the formation of 6-membered lactones (6-endo *vs.* 5-exo cyclization). Starting from **1c**, a 1/1.2 mixture of **2c_exo_**/**2c_endo_** products was obtained with Pd dimer **I** alone, while in the presence of the additives, **2c_endo_** largely prevailed (the selectivity reached 1/7.3 with **4p**). In the case of 4-hexynoic acid **1d**, the catechol additives even switched the selectivity and the 6-endo cyclized product **2d_endo_** could be obtained with a selectivity of up to 1/2.3 in the case of 1,2,3-benzenetriol **4p**.

We then investigated the cycloisomerization of internal 5-alkynoic acids which has been scarcely achieved, using gold complexes.^20^ The Pd dimer **I** was previously shown to efficiently cycloisomerize 6-bromo-5-hexynoic acid **1e**, a relatively activated substrate, and the corresponding 6-membered alkylidene-lactone **2e** could be quantitatively obtained within 5 h (entry 3). In the presence of 4-nitrocatechol **4x**, the reaction time was again drastically decreased. Full conversion was achieved in only 20 min using 5 mol% of the additive. Note that **2e** is obtained exclusively in its *Z* form as the result of *anti* addition of the carboxylate to the Pd-coordinated alkyne. The catechol additive has an even more striking influence on the cyclization of 5-heptynoic acid **1f** (entry 4). No reaction occurred with Pd dimer **I** alone, even after prolonged heating, but in the presence of 4-nitrocatechol (30 mol%), complete conversion was achieved within 60 h. Under these conditions, simple hydration of the alkyne tends to compete with the cyclosiomerization,^9^ but the targeted 6-membered alkylidene-lactone **2f** was obtained in good yield (70%), again as a unique isomer (*Z*).

## Conclusions

The precise mode of action of SCS indenediide Pd complexes in the cycloisomerization of alkynoic acids has been determined thanks to DFT and kinetic studies. Cooperation between the Pd center and the backbone of the pincer ligand has been confirmed computationally. In addition, the mechanistic study revealed the involvement of a second molecule of alkynoic acid all along the catalytic cycle: it acts as a proton shuttle in the activation of the acid and in the protonolysis of the intermediate vinyl Pd species, it also directs the nucleophilic attack of the carboxylic acid on the π-coordinated alkyne (*anti* addition). The possibility of replacing the second substrate molecule by an additive was investigated. A variety of H-bond donors were screened and polyols featuring proximal hydroxyl groups led to significant catalytic enhancement. Catechol derivatives substituted by electron-withdrawing groups proved highly beneficial on both activity and selectivity. The impact of 4-nitrocatechol **4x** and 1,2,3-benzenetriol **4p** on the cyclization of internal alkynoic acids is spectacular (endo/exo selectivities up to 7.3/1 and 60-fold increase in reactivity were achieved).

This work also illustrates the interest to perform mechanistic studies on catalytic transformations. It enables us to understand the key factors influencing their efficiency and/or selectivity, and paves the way for further improvement. In this study, the combination of metal–ligand cooperation and proton shuttling by a second molecule of substrate has been pointed out. The ability of protic substrates, solvents or water molecules to act as proton shuttles in transition-metal catalyzed transformations has been occasionally proposed and supported by DFT calculations.^21^ We have shown here that this role can be transferred to an H-bond donor additive, and that optimizing the structure of this additive can significantly enhance the catalytic activity and selectivity. Proton shuttling is relatively well-established in organic synthesis and polymerization catalysis.^22^ We believe it is also highly relevant in transition-metal catalysis^23^ and can be applied to a variety of transformations.

## Supplementary Material

Supplementary information
