# Supplementary material for: A case study of proton shuttling in palladium catalysis
Source: Chem Sci. 2015 Dec 7;7(3):2179–87. doi: 10.1039/c5sc04232a (PMC5968755; doi:10.1039/c5sc04232a)
Supplement: Supplementary file 1 [file SC-007-C5SC04232A-s001.pdf]

Electronic Supplementary Material (ESI) for Chemical Science.
This journal is © The Royal Society of Chemistry 2015

Supporting Information for:

A case study of proton shuttle in palladium catalysis

Julien Monot,[a,b] Paul Brunel,[a,b] Christos E. Kefalidis,[c] Noel Ángel Espinosa-Jalapa,[a,b] Laurent Maron,*[c] Blanca Martin-Vaca,*[a,b] and Didier Bourissou*[a,b]

[a]Université de Toulouse, UPS, 118 route de Narbonne, F-31062 Toulouse, France

[b]CNRS, LHFA UMR5069, F-31062 Toulouse, France

[c]Université de Toulouse, INSA, UPS, LCPNO, CNRS, UMR 5215 CNRS-UPS-INSA, 135 avenue de Rangueil, 31400 Toulouse, France

Corresponding author emails: laurent.maron@irsamc.ups-tlse.fr, bmv@chimie.ups-tlse.fr, dbouriss@chimie.ups-tlse.fr

Table of contents

I. Materials and methods S2

II. Kinetic studies S2

II.a. General sampling procedure S2

II.b. Partial order determination: S2

II.b.1. Partial order determination for [Pd] S2

II.b.2. Partial order determination for 5-hexynoic acid **1a**. S5

II.b.3. Partial order determination for 5-hexynoic acid **1a** at room temperature. S7

II.b.3. Partial order determination for 5-hexynoic acid **1a** in presence of 1 mol% of **4u**. S9

II.b.4. Partial order determination for 5-hexynoic acid **1a** in presence of 5 mol% of **4u**. S11

II.b.5. Partial order determination for 5-hexynoic acid **1a** in presence of 10 mol% of **4u**. S13

II.b.6. Partial order determination for 5-hexynoic acid **1a** in presence of 20 mol% of **4u**. S15

III. Study of the self association 5-hexynoic acid in $CHCl_3$ by IR spectroscopy. S17

VI. ^{31}P NMR analysis of the Palladium indenediide dimer I at variable temperature. S19

VII. General procedure for cycloisomerization of alkynoic acids with additives S20

VIII. Characterization of product 2f: S23

IX. Computational details S26

References S46

I. Materials and methods

All reactions and manipulations were carried out under an atmosphere of dry argon using standard Schlenk techniques. Dry, oxygen–free solvents were employed. All organic reagents were obtained from commercial sources and dried overnight with P_2O_5 before use. Substrates **1c-f** and Catalyst **I** were prepared following the literature procedures.[S1] ^{31}P, ^{1}H and ^{13}C spectra were recorded on Bruker Avance 300 and 500. ^{31}P, ^{1}H and ^{13}C chemical shifts are expressed with a positive sign, in parts per million, relative to external 85% H_3PO_4 and Me_4Si. Unless otherwise stated, NMR spectra were recorded at 293 K. IR spectra were recorded on Thermoscientific FTIR 6700 equipped with a DLaGS detector using a CaF_2 cell.

II. Kinetic studies

II.a. General sampling procedure

All the kinetic studies were set up under Argon atmosphere. 0.7 mL of deuterated chloroform as solvent was used for all experiments. At every sampling time, a ^{1}H NMR spectra was recorded and the conversion was determined related to the integration of the alkyne proton of the substrate (δ = 1.99 ppm) and the methylene protons of the lactone (δ = 4.30 ppm).

II.b. Partial order determination:

The partial order of each reaction's components (substrate and catalyst) was determined by the initial rate method. The data of the concentration of product versus time plot were fitted with Excel. The obtained slope of the linear fitting represents the initial rate. The partial order was then determined by plotting the initial rates versus the initial concentrations.

II.b.1. Partial order determination for [Pd]

To determine the partial order of the reaction on catalyst, the initial kinetic profiles at different initial concentrations of palladium center were recorded. The final data were obtained by averaging the results of three independent trials for each experiment.

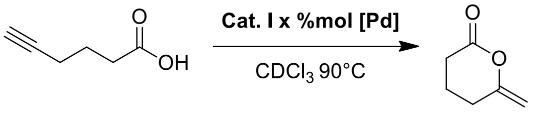

1a
0.14 M

2a

Experiment	% mol [Pd]	x mg of Cat. **I**	Initial concentration [Pd]
1	3	1.5	0.0042
2	5	2.5	0.007
3	7.5	3.8	0.0105
4	10	5.1	0.014

Figure S1. Reaction conditions for the partial order determination of [Pd].

General procedure: 10.8 μL of 5-hexynoic acid **1a** (0.0098 mmol, 0.14 M) a specific quantity of Catalyst **I** according to the above table and 0.7 mL of $CDCl_3$ were introduced in a pressure NMR tube. The

reaction mixture was heated at 90°C and ^{1}H NMR spectra were recorded every five minutes until a conversion of 10%.

a) *Serie 1:*

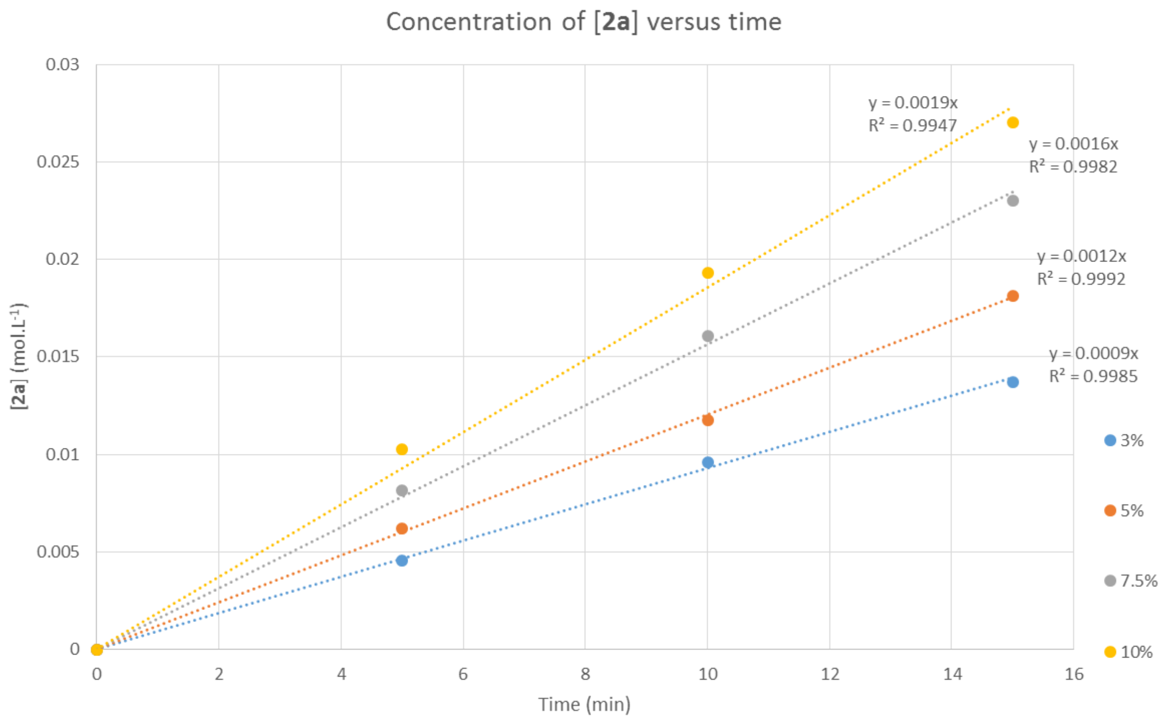

b) *Serie 2:*

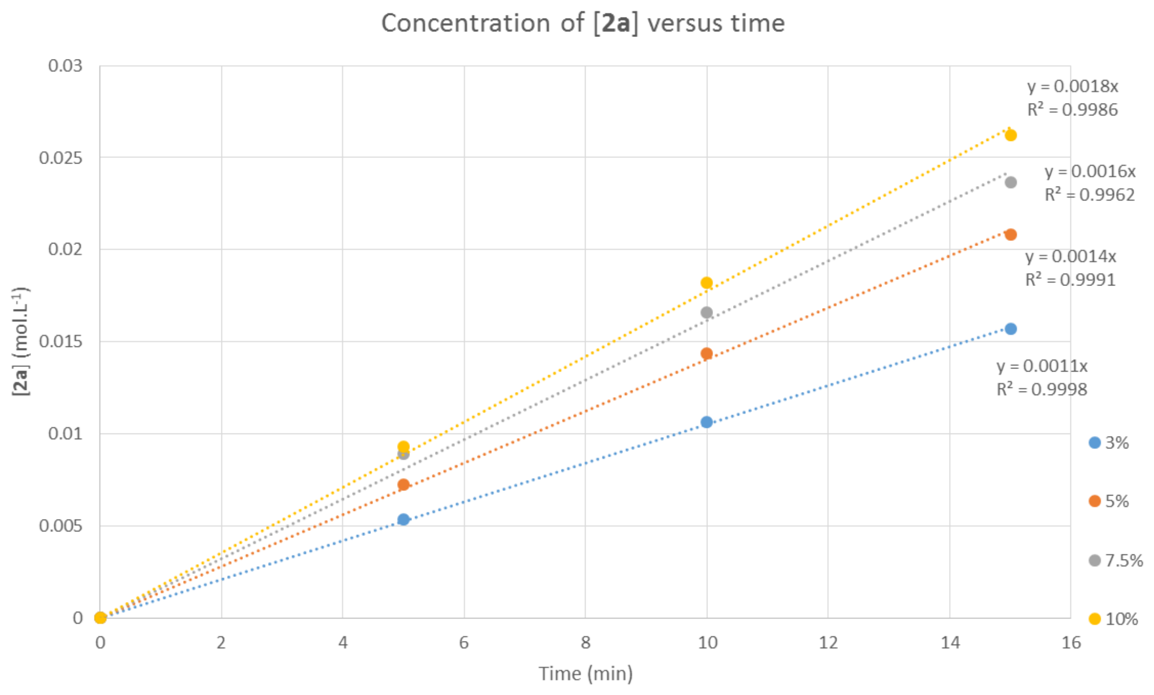

c) Serie 3:

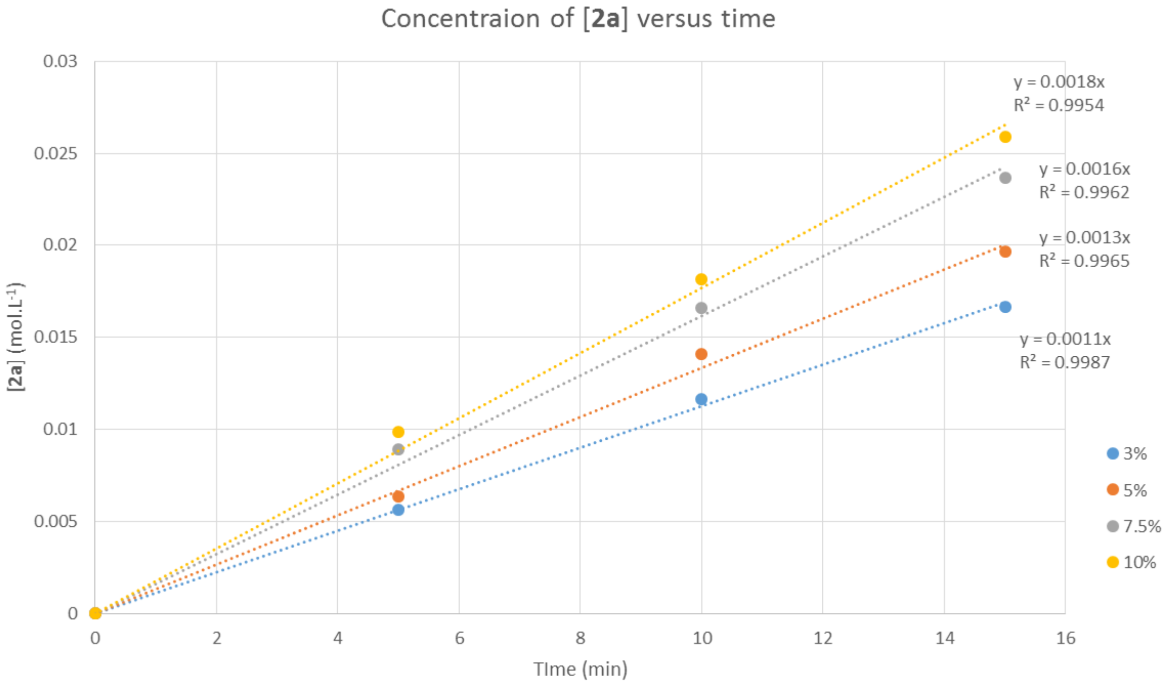

Figure S2. Determination of the intial rate through a linear fit to the equation: [**2a**] = V_0[t] at different concentration of palladium center.

Table S1. Initial rates determined for different palladium concentrations.

% mol Pd	[Pd] ($mol.L^{-1}$)	$V_0(1)$ ($mol.L^{-1}.min^{-1}$)	$V_0(2)$ ($mol.L^{-1}.min^{-1}$)	$V_0(3)$ ($mol.L^{-1}.min^{-1}$)	Average of V_0 ($mol.L^{-1}.min^{-1}$)
3	0.0042	0.0009	0.0011	0.0011	0.00103
5	0.007	0.0012	0.0013	0.0014	0.0013
7.5	0.0105	0.0016	0.0016	0.0016	0.0016
10	0.014	0.0019	0.0018	0.0018	0.00183

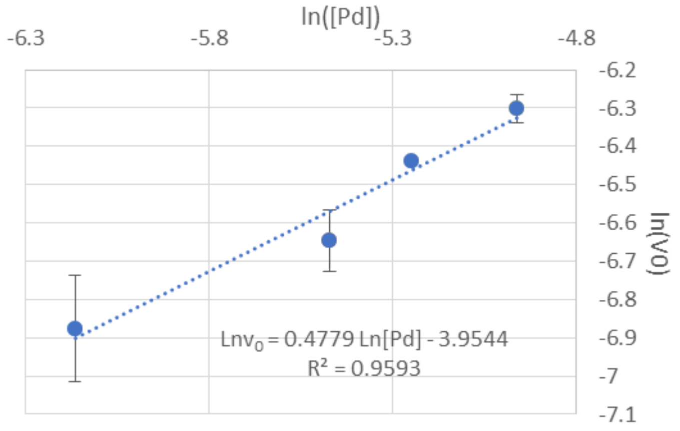

Figure S3. The dependence of initial rates on the palladium center loading.

*II.b.2. Partial order determination for 5-hexynoic acid **1a**.*

To determine the partial order of the reaction in 5-hexynoic acid, the initial kinetic profiles at different initial concentrations of 5-hexynoic acid were recorded. The final data were obtained by averaging the results of three independent trials for each experiment.

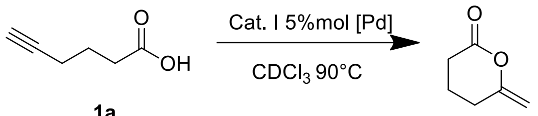

1a
x M
2a

Experiment	x ⍰L of 5-hexynoic acid	Initial concentration [**1a**] ($mol.L^{-1}$)
1	10.8	0.14
2	19.3	0.25
3	38.6	0.5
4	57.9	0.75

Figure S4. Reaction conditions for the partial order determination of [**1a**].

<u>General procedure:</u> 2.53 mg (5 %mol [Pd]) of Catalyst **I**, a specific quantity of 5-hexynoic acid **1a** according to the above table and 0.7 mL of $CDCl_3$ were introduced in a pressure NMR tube. The reaction mixture was heated at 90°C and ^1H NMR spectra were recorded every five minutes until a conversion of 30%.

a) *Serie 1:*

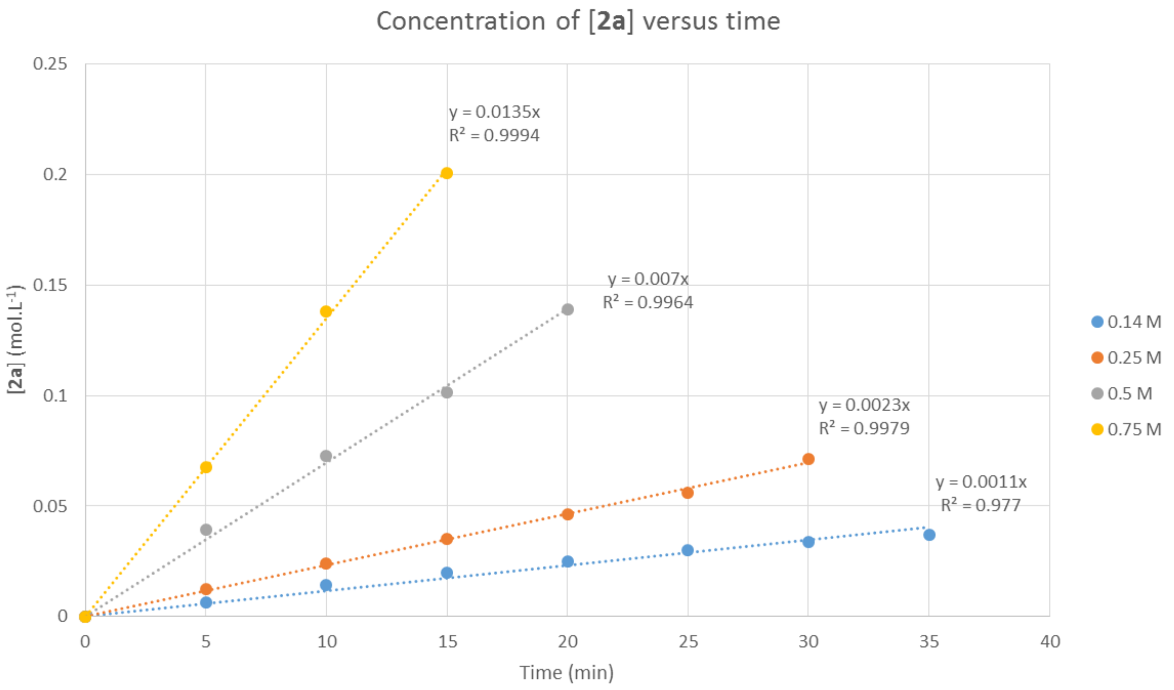

b) Serie 2:

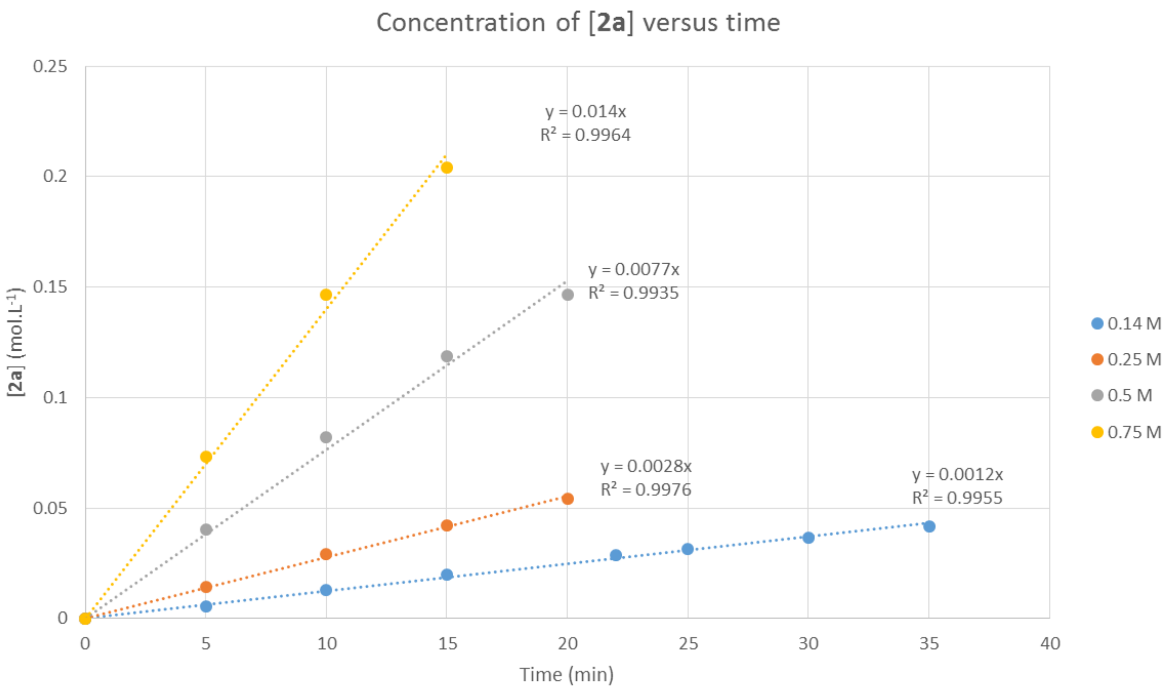

c) Serie 3:

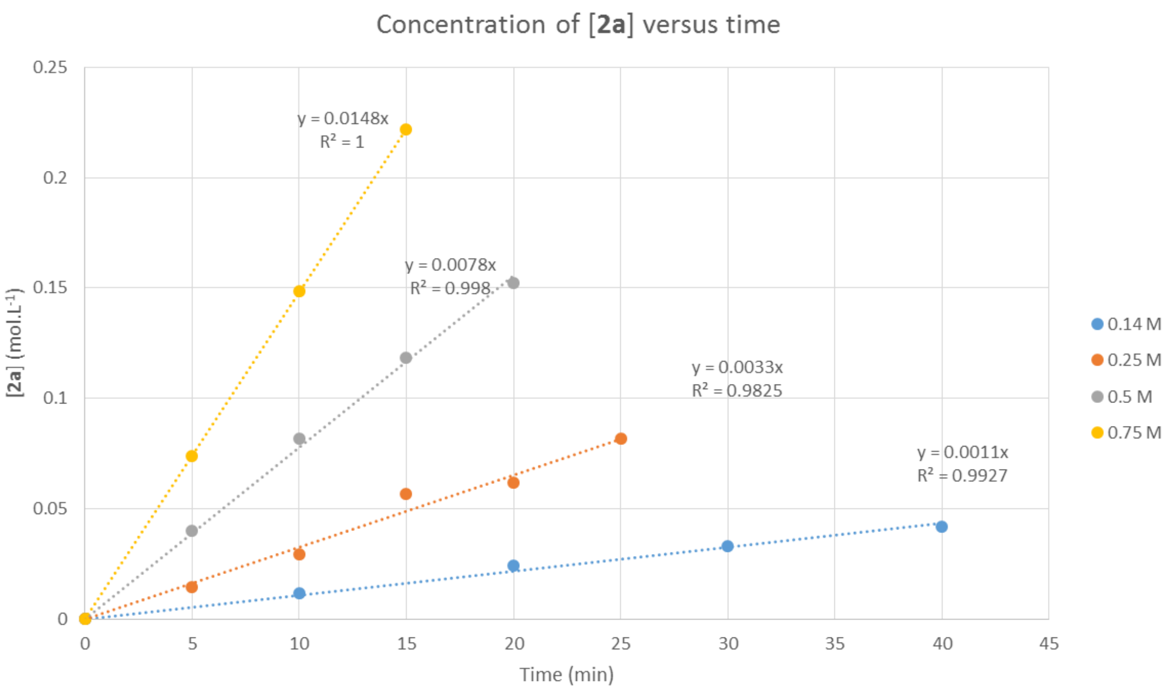

Figure S5. Determination of the intial rate through a linear fit to the equation: [**2a**] = V_0[t] at different concentration of [**1a**].

Table S2. Initial rates determined for different concentrations of substrate **1a**.

[**1a**] (mol.L^{-1})	$V_0(1)$ (mol.L^{-1}.min^{-1})	$V_0(2)$ (mol.L^{-1}.min^{-1})	$V_0(3)$ (mol.L^{-1}.min^{-1})	Average of V_0 (mol.L^{-1}.min^{-1})
0.14	0.0011	0.0012	0.0011	0.00115
0.25	0.0033	0.0028	0.0023	0.00305
0.5	0.0078	0.0077	0.007	0.00775
0.75	0.0148	0.014	0.0135	0.0144

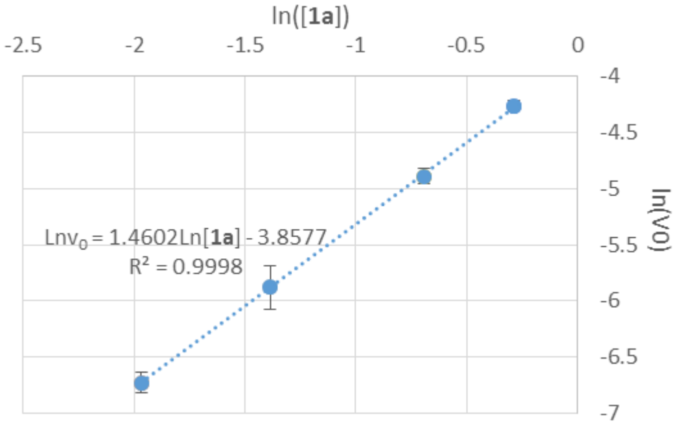

Figure S6. The dependence of initial rates on [**1a**].

*II.b.3. Partial order determination for 5-hexynoic acid **1a** at room temperature.*

To determine the partial order of the reaction on 5-hexynoic acid at room temperature, the initial kinetic profiles at different initial concentrations of 5-hexynoic acid were recorded.

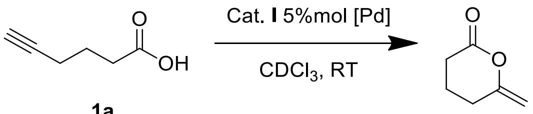

1a
x M

2a

Experiment	x μL of 5-hexynoic acid	Initial concentration [**1a**] (mol.L^{-1})
1	10.8	0.14
2	19.3	0.25
3	38.6	0.5
4	57.9	0.75

Figure S7. Reaction conditions for the partial order determination of [**1a**].

General procedure: 2.53 mg (5 %mol [Pd]) of Catalyst **I**, a specific quantity of 5-hexynoic acid **1a** according to the above table and 0.7 mL of $CDCl_3$ were introduced in a pressure NMR tube. ^{1}H NMR spectra were recorded at room temperature.

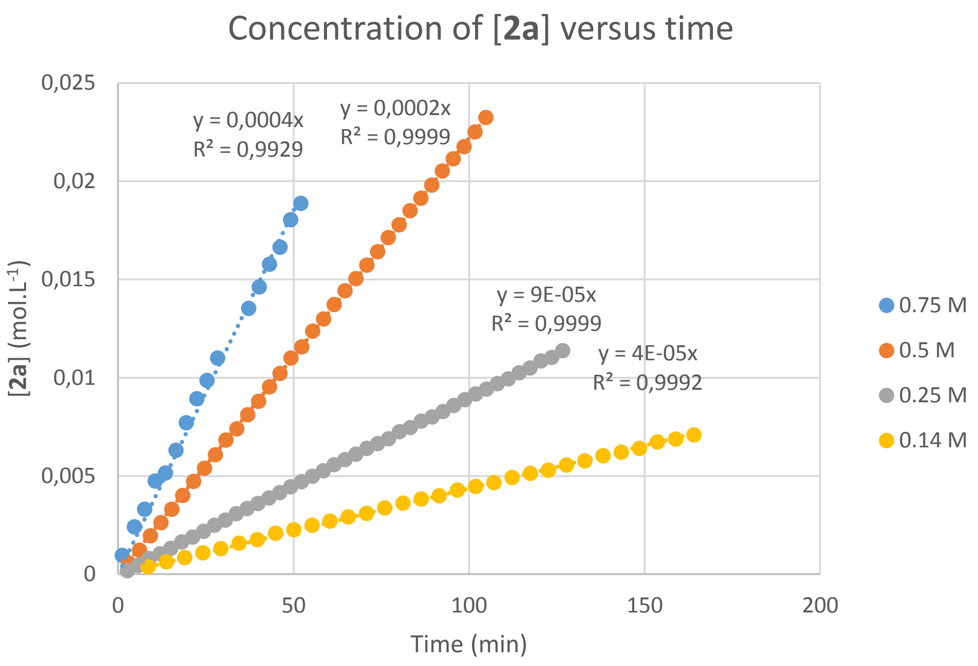

Figure S8. Determination of the intial rate through a linear fit to the equation: [**2a**] = V_0[t] at different concentration of [**1a**].

Table S3. Initial rates determined for different concentrations of substrate **1a**.

[**1a**] (mol.L^{-1})	V_0 (mol.L^{-1}.min^{-1})
0.14	4 x 10^{-5}
0.25	9 x 10^{-5}
0.5	0.0002
0.75	0.0004

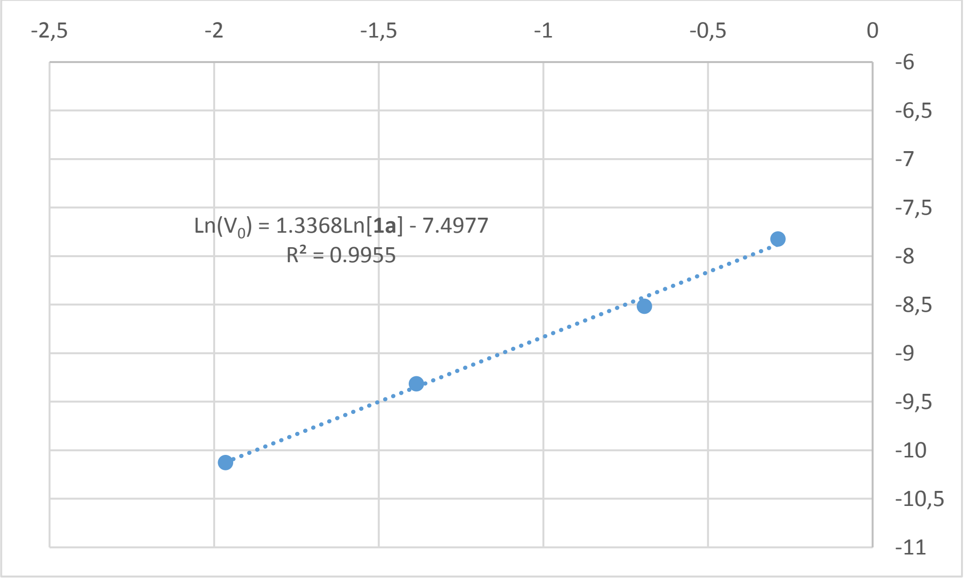

Figure S9. The dependence of initial rates on [**1a**] at room temperature.

*II.b.3. Partial order determination for 5-hexynoic acid **1a** in presence of 1 mol% of Tetrachlorocatechol **4u**.*

To determine the partial order of the reaction on 5-hexynoic acid in presence of 1 mol% of Tetrachlorocatechol **4u**, the initial kinetic profiles at different initial concentrations of 5-hexynoic acid were recorded. The final data were obtained by averaging the results of two independent trials for each experiment.

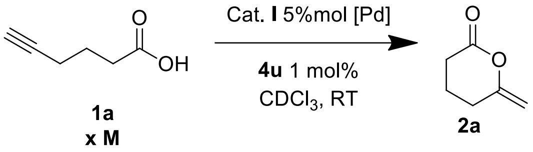

Experiment	x µL of 5-hexynoic acid	Initial concentration [**1a**] (mol.L^{-1})
1	10.8	0.14
2	19.3	0.25
3	38.6	0.5
4	57.9	0.75

Figure S10. Reaction conditions for the partial order determination of [**1a**].

<u>General procedure:</u> 2.53 mg (5 %mol [Pd]) of Catalyst **I**, a specific quantity of 5-hexynoic acid **1a** according to the above table and 0.7 mL of a solution of tetracholocatechol **4u** in $CDCl_3$ ([0.0014 M]: 3.47 mg of **4u** in 10 mL of $CDCl_3$) were introduced in a pressure NMR tube. ^{1}H NMR spectra were recorded at room temperature.

a) Serie 1:

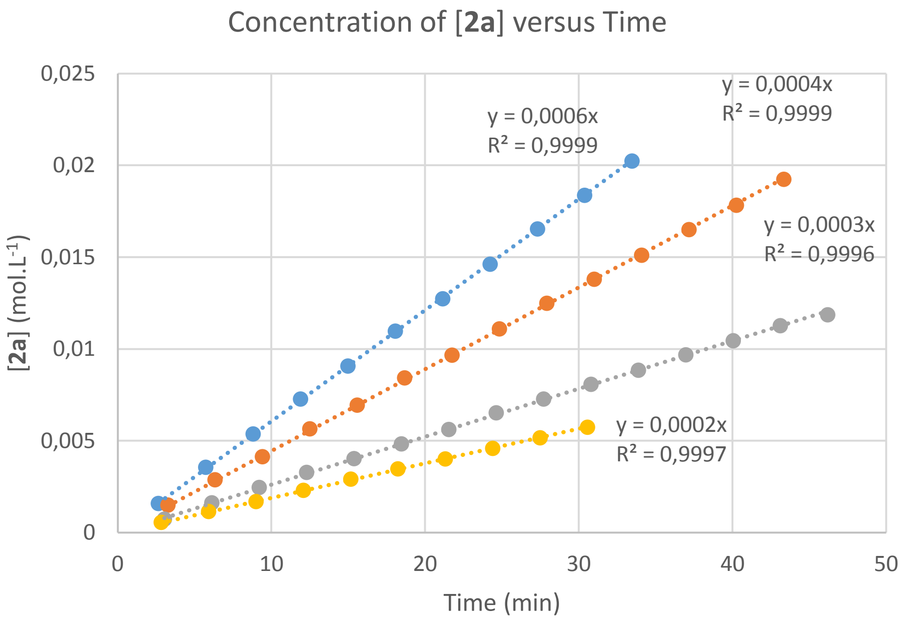

b) Serie 2:

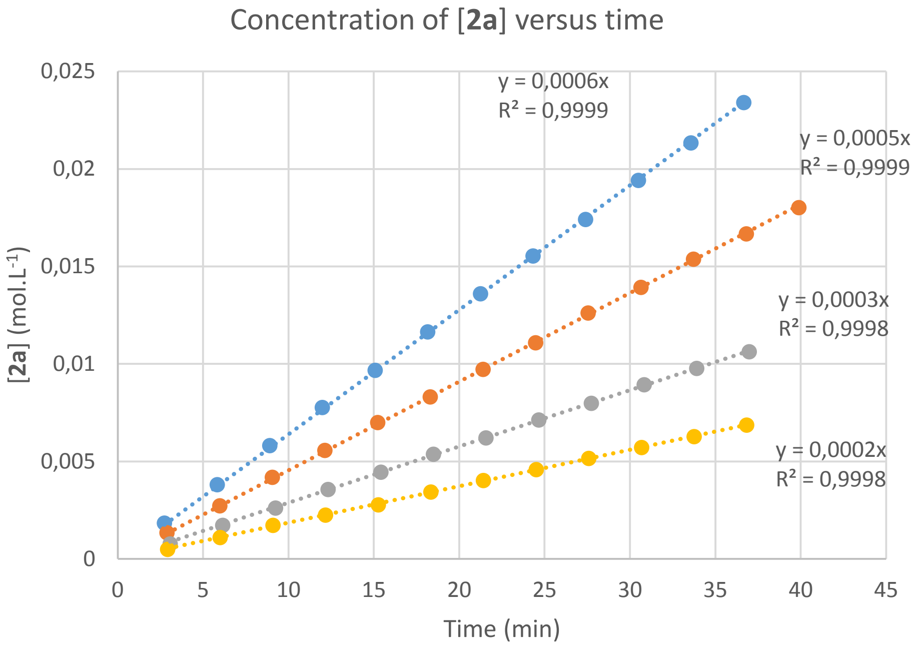

Figure S11. Determination of the intial rate through a linear fit to the equation: [**2a**] = V_0[t] at different concentration of [**1a**].

Table S4. Initial rates determined for different concentrations of substrate **1a**.

[**1a**] (mol.L^{-1})	V_0(1) (mol.L^{-1}.min^{-1})	V_0(2) (mol.L^{-1}.min^{-1})	Average of V_0 (mol.L^{-1}.min^{-1})
0.14	0.0002	0.0002	0.0002
0.25	0.0003	0.0003	0.0003
0.5	0.0004	0.0005	0.00045
0.75	0.0006	0.0006	0.0006

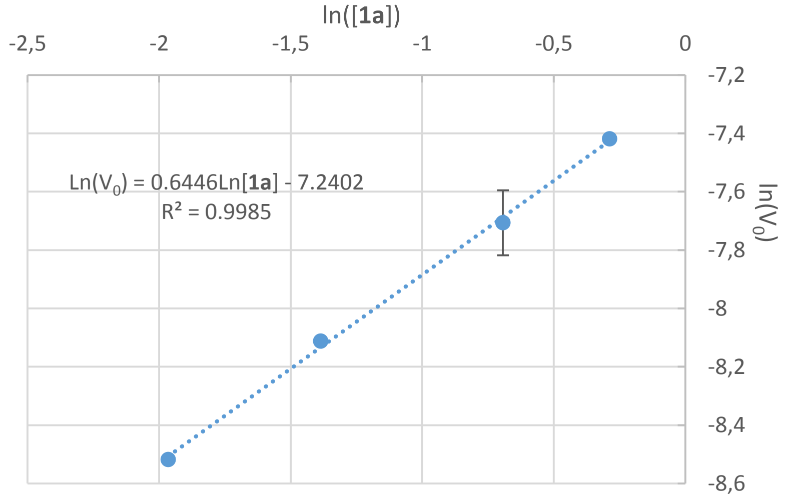

Figure S12. The dependence of initial rates on [**1a**] in presence of tetrachlorocatechol (1 mol%).

*II.b.4. Partial order determination for 5-hexynoic acid **1a** in presence of 5 mol% of Tetrachlorocatechol **4u**.*

To determine the partial order of the reaction on 5-hexynoic acid in presence of 5 mol% of Tetrachlorocatechol **4u**, the initial kinetic profiles at different initial concentrations of 5-hexynoic acid were recorded. The final data were obtained by averaging the results of two independent trials for each experiment.

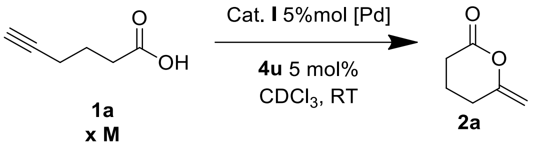

Experiment	x µL of 5-hexynoic acid	Initial concentration [**1a**] (mol.L^{-1})
1	10.8	0.14
2	19.3	0.25
3	38.6	0.5
4	57.9	0.75

Figure S13. Reaction conditions for the partial order determination of [**1a**].

<u>General procedure:</u> 2.53 mg (5 %mol [Pd]) of Catalyst **I**, a specific quantity of 5-hexynoic acid **1a** according to the above table and 0.7 mL of a solution of tetracholocatechol **4u** in $CDCl_3$ ([0.007 M]: 8.67 mg of **4u** in 5 mL of $CDCl_3$) were introduced in a pressure NMR tube. ^{1}H NMR spectra were recorded at room temperature.

a) *Serie 1:*

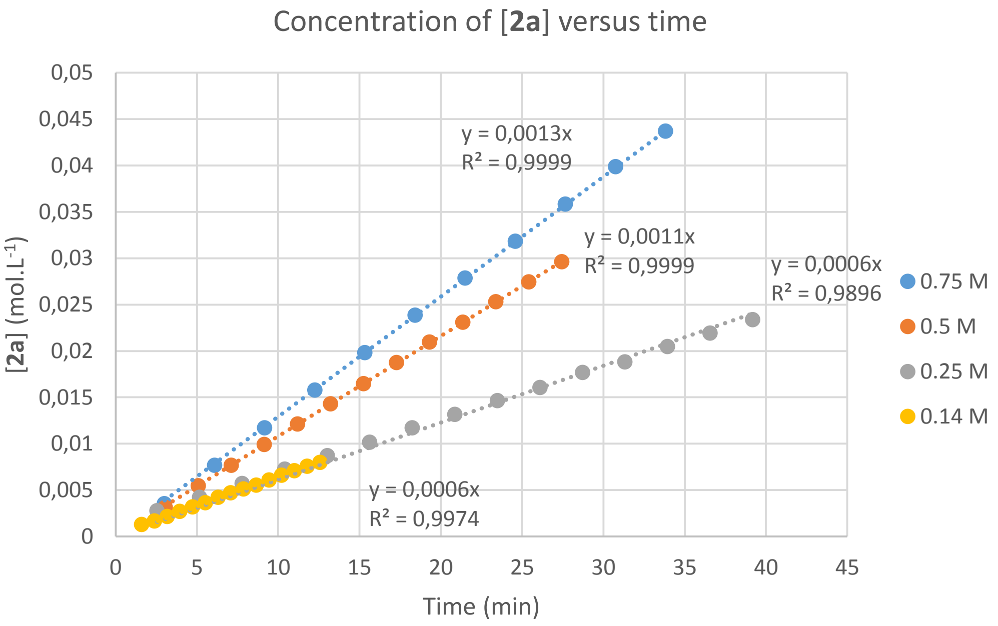

b) *Serie 2:*

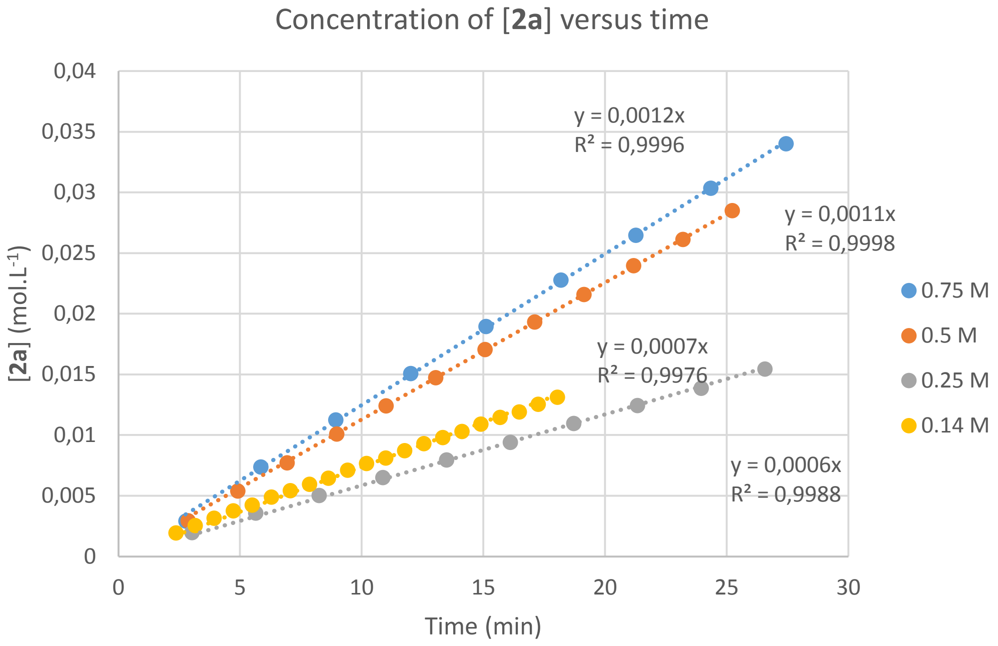

Figure S14. Determination of the intial rate through a linear fit to the equation: [**2a**] = V_0[t] at different concentration of [**1a**].

Table S5. Initial rates determined for different concentrations of substrate **1a**.

[**1a**] ($mol.L^{-1}$)	$V_0(1)$ ($mol.L^{-1}.min^{-1}$)	$V_0(2)$ ($mol.L^{-1}.min^{-1}$)	Average of V_0 ($mol.L^{-1}.min^{-1}$)
0.14	0.0006	0.0007	0.00065
0.25	0.0006	0.0006	0.0006
0.5	0.0011	0.0011	0.0011
0.75	0.0013	0.0012	0.00125

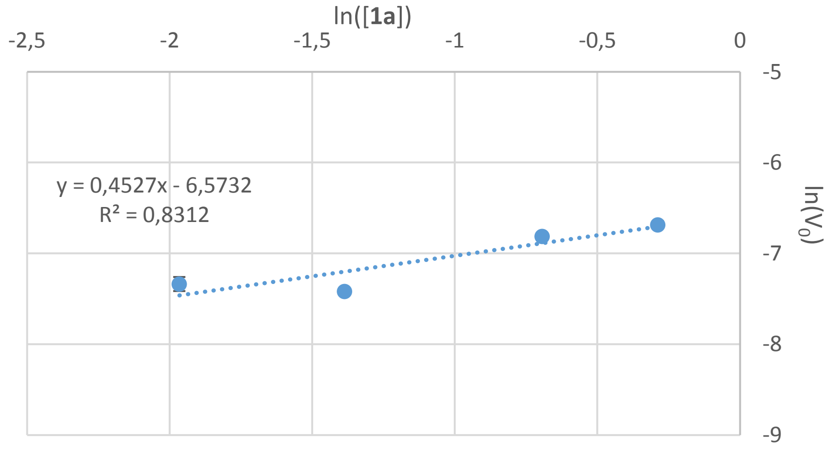

Figure S15. The dependence of initial rates on [**1a**] in presence of tetrachlorocatechol (5 mol%).

*II.b.5. Partial order determination for 5-hexynoic acid **1a** in presence of 10 mol% of Tetrachlorocatechol **4u**.*

To determine the partial order of the reaction on 5-hexynoic acid in presence of 10 mol% of Tetrachlorocatechol **4u**, the initial kinetic profiles at different initial concentrations of 5-hexynoic acid were recorded. The final data were obtained by averaging the results of two independent trials for each experiment.

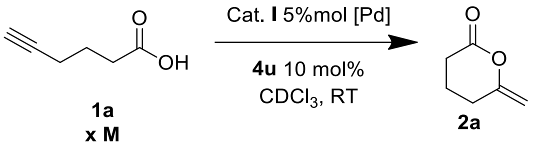

Experiment	x L of 5-hexynoic acid	Initial concentration [**1a**] (mol.L^{-1})
1	10.8	0.14
2	19.3	0.25
3	38.6	0.5
4	57.9	0.75

Figure S16. Reaction conditions for the partial order determination of [**1a**].

<u>General procedure:</u> 2.53 mg (5 %mol [Pd]) of Catalyst **I**, 2.43 mg of tetrachlorocatechol and a specific quantity of 5-hexynoic acid **1a** according to the above table were introduced in a pressure NMR tube with 0.7 mL of $CDCl_3$. ^{1}H NMR spectra were recorded at room temperature.

a) Serie 1:

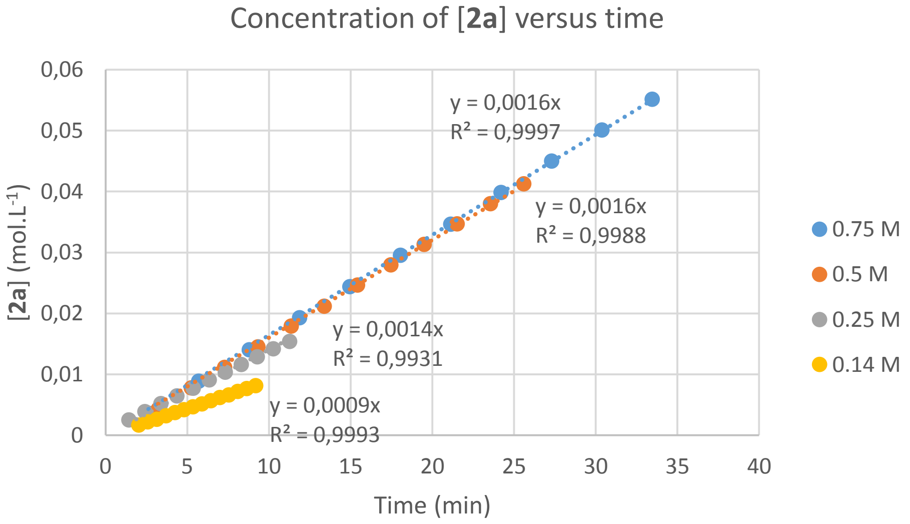

b) Serie 2:

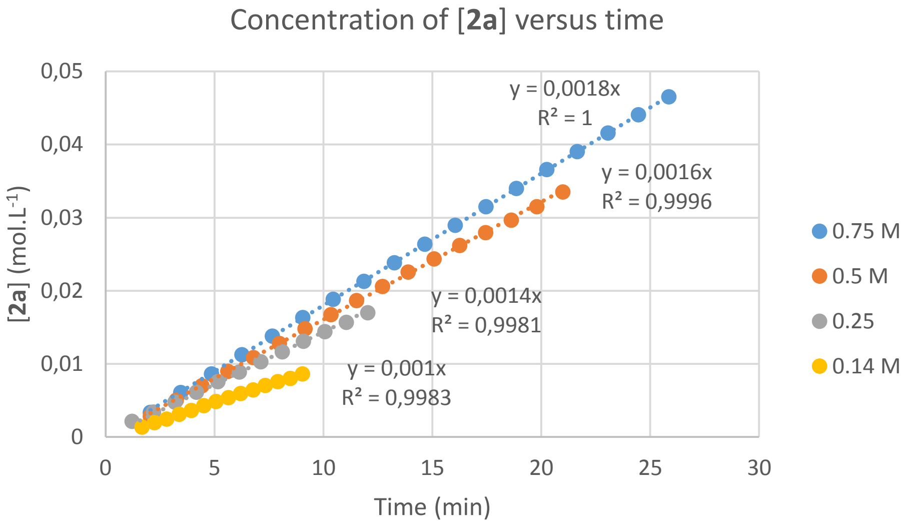

Figure S17. Determination of the intial rate through a linear fit to the equation: [**2a**] = V_0[t] at different concentration of [**1a**].

Table S6. Initial rates determined for different concentrations of substrate **1a**.

[**1a**] ($mol.L^{-1}$)	$V_0(1)$ ($mol.L^{-1}.min^{-1}$)	$V_0(2)$ ($mol.L^{-1}.min^{-1}$)	Average of V_0 ($mol.L^{-1}.min^{-1}$)
0.14	0.0009	0.001	0.00095
0.25	0.0013	0.0014	0.00135
0.5	0.0016	0.0016	0.0016
0.75	0.0016	0.0018	0.0017

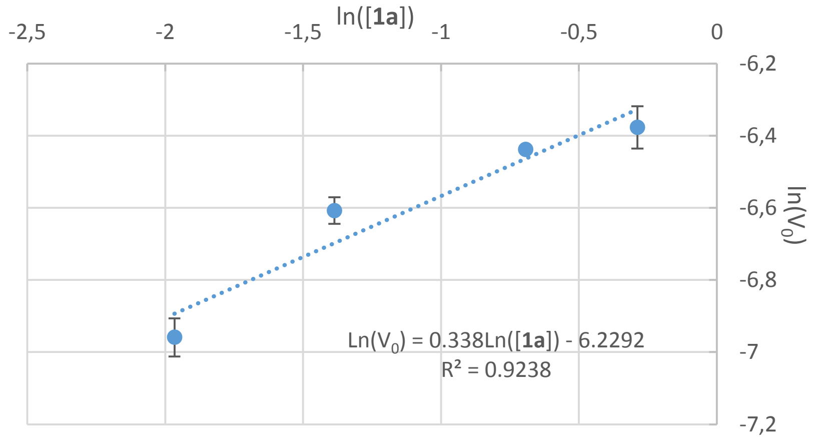

Figure S18. The dependence of initial rates on [**1a**] in presence of tetrachlorocatechol (10 mol%).

*II.b.6. Partial order determination for 5-hexynoic acid **1a** in presence of 20 mol% of Tetrachlorocatechol **4u**.*

To determine the partial order of the reaction in 5-hexynoic acid in presence of 20 mol% of Tetrachlorocatechol **4u**, the initial kinetic profiles at different initial concentrations of 5-hexynoic acid were recorded. The final data were obtained by averaging the results of two independent trials for each experiment.

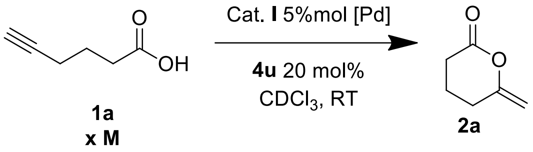

Experiment	x [illegible]L of 5-hexynoic acid	Initial concentration [**1a**] (mol.L^{-1})
1	10.8	0.14
2	19.3	0.25
3	38.6	0.5
4	57.9	0.75

Figure S19. Reaction conditions for the partial order determination of [**1a**].

<u>General procedure:</u> 2.53 mg (5 %mol [Pd]) of Catalyst **I**, 4.86 mg of tetrachlorocatechol and a specific quantity of 5-hexynoic acid **1a** according to the above table were introduced in a pressure NMR tube with 0.7 mL of $CDCl_3$. ^{1}H NMR spectra were recorded at room temperature.

a) Serie 1:

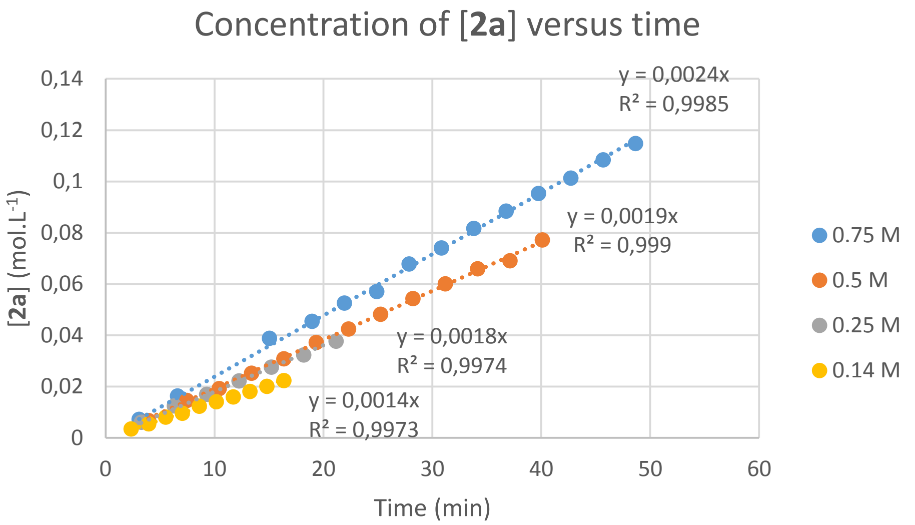

b) Serie 2:

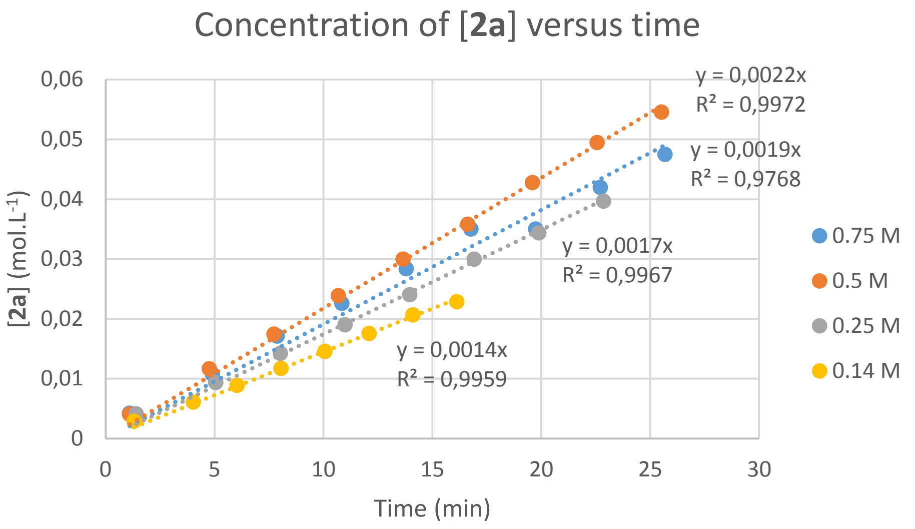

Figure S20. Determination of the intial rate through a linear fit to the equation: [**2a**] = V_0[t] at different concentration of [**1a**].

Table S7. Initial rates determined for different concentrations of substrate **1a**.

[**1a**] ($mol.L^{-1}$)	$V_0(1)$ ($mol.L^{-1}.min^{-1}$)	$V_0(2)$ ($mol.L^{-1}.min^{-1}$)	Average of V_0 ($mol.L^{-1}.min^{-1}$)
0.14	0.0014	0.0014	0.0014
0.25	0.0018	0.0017	0.00175
0.5	0.0019	0.0021	0.002
0.75	0.0024	0.002	0.0022

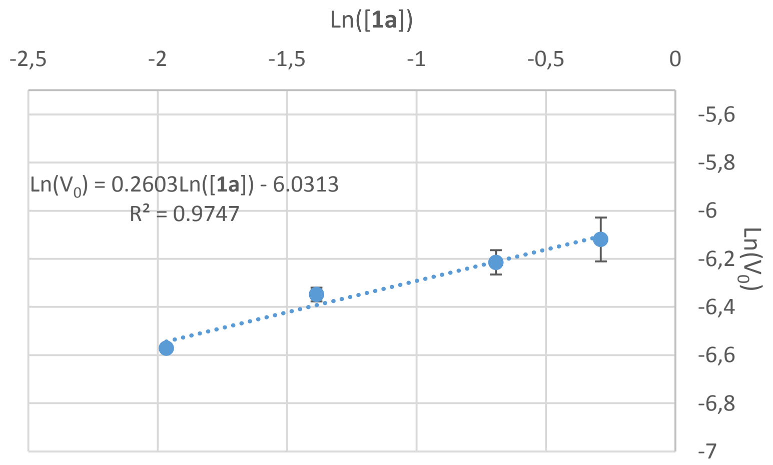

Figure S21. The dependence of initial rates on [**1a**] in presence of tetrachlorocatechol (1 mol%).

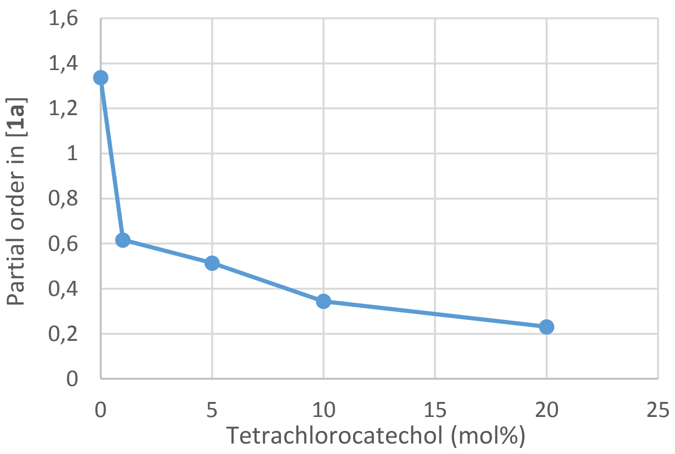

Figure S22. Partial order of 5-hexynoic **1a** versus the quantity of tetrachlorocatechol **4u**.

III. Study of the self association 5-hexynoic acid in $CHCl_3$ by IR spectroscopy.

The self-association of 5-hexynoic acid was evidenced by IR spectroscopy in $CHCl_3$ at different acid concentrations (*Vide Infra*). IR spectra were recorded with a resolution of 4 cm^{-1} in 16 scans. Two well defined absorptions bands were observed, at 1711 and 1751 cm^{-1} corresponding to the dimeric and monomer forms, respectively. The observed bands are shown in Figure S23 at different concentrations and reported in Table S8 with the intensities ratio.

Table S8. Ratio of the intensity of the IR signal corresponding to the dimer form (1711 cm^{-1}) versus the one corresponding to the monomer form (1750 cm^{-1}) for 5-hexynoic acid **1a**.

**Concentration in [1a] (mol.L^{-1})**	**Intensity of the IR signal at 1711 cm^{-1}**	**Intensity of the IR signal at 1750 cm^{-1}**	**Ratio dimer/monomer**
0.5	1.91	0.18	10.6
0.25	1.0	0.11	9.09
0.14	0.58	0.0774	7.49
0.1	0.417	0.0615	6.78
0.075	0.297	0.0483	6.15
0.05	0.194	0.0373	5.20
0.025	0.0875	0.0238	3.67
0.01	0.0346	0.014	2.47
0.0075	0.0525	0.0172	3.05
0.005	0.0137	0.00931	1.47
0.0025	0.00503	0.00506	0.99
0.001	0.00247	0.00359	0.69

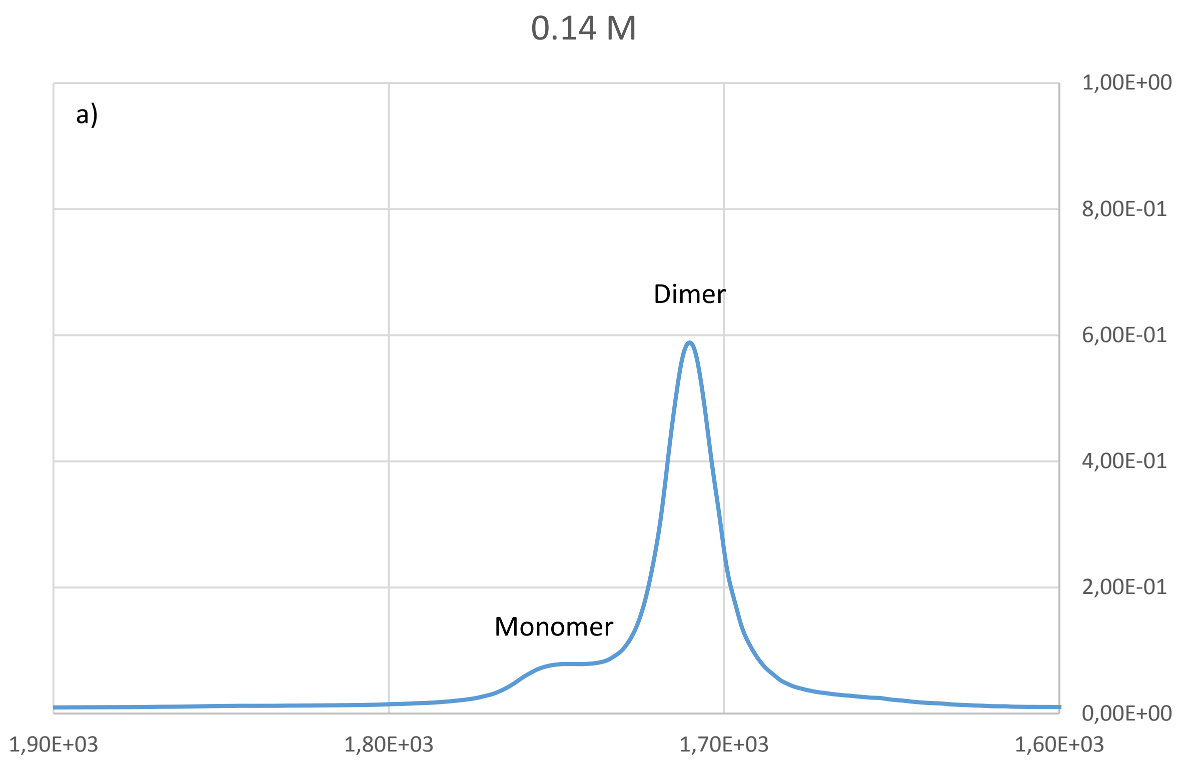
0.14 M
a)
Dimer
Monomer
1,00E+00
8,00E-01
6,00E-01
4,00E-01
2,00E-01
0,00E+00
1,90E+03
1,80E+03
1,70E+03
1,60E+03

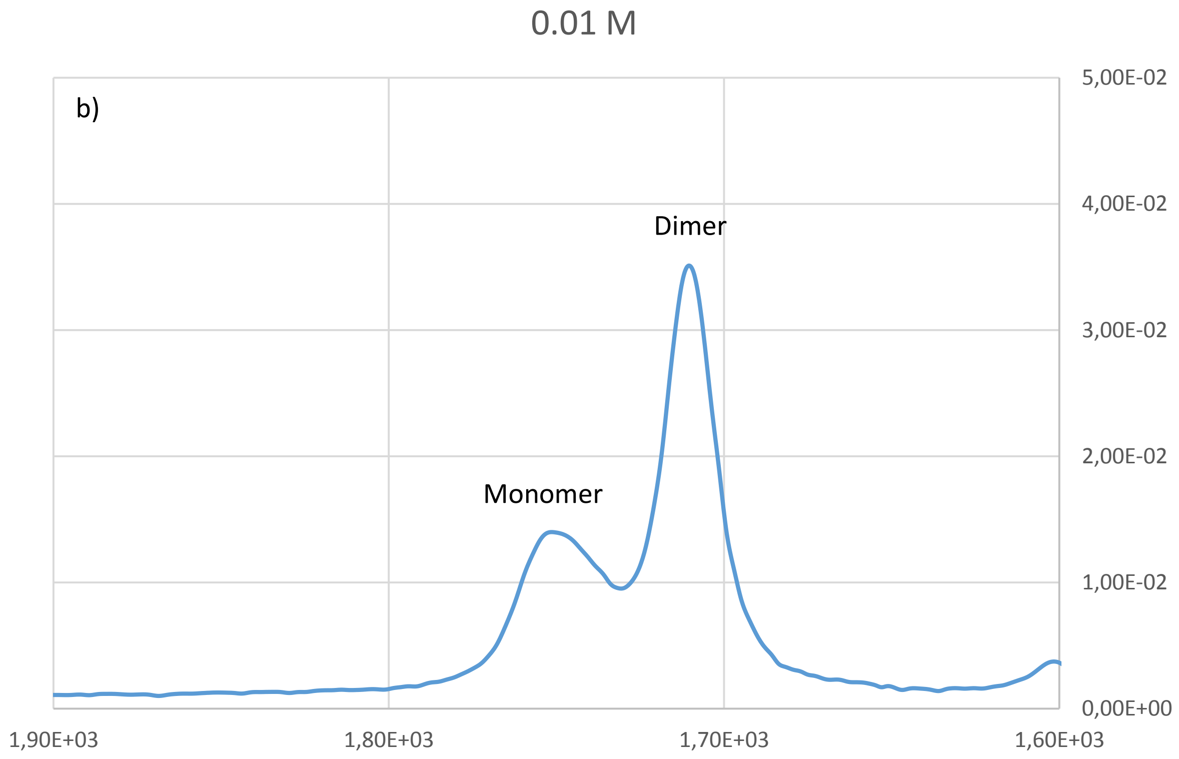
0.01 M
b)
Dimer
Monomer
5,00E-02
4,00E-02
3,00E-02
2,00E-02
1,00E-02
0,00E+00
1,90E+03
1,80E+03
1,70E+03
1,60E+03

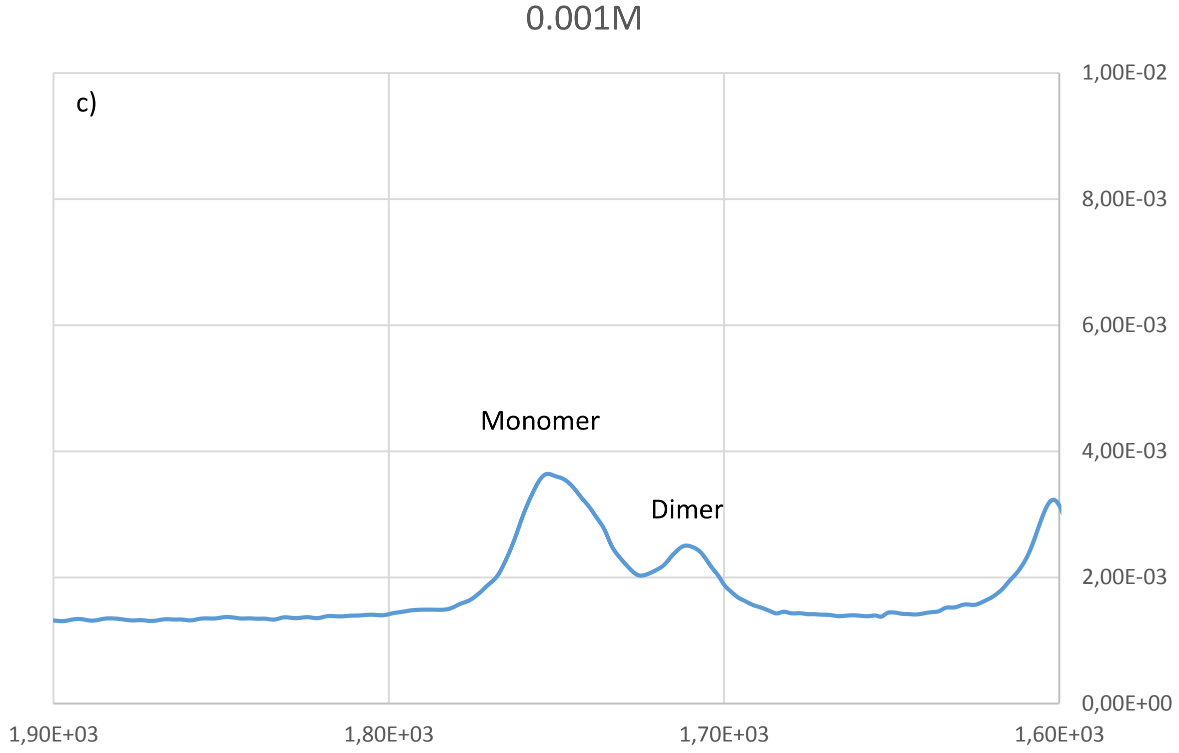

Figure S23. IR spectrum of the 5-hexynoic acid at different concentrations in chloroform: (a) [**1a**] = 0.14 M; (b) [**1a**] = 0.01 M and (c) [**1a**] = 0.001M.

VI. ^{31}P NMR analysis of the Palladium indenediide dimer I at variable temperature.

The dissociation-association behavior of the Palladium indenediide dimer **I** was evidenced by ^{31}P NMR spectroscopy at variable temperature using a 400 MHz NMR spectrometer. The association activation barrier was estimated from these experiments (Figure S24), using the following formula:

$$\Delta G^{\#} = RT_C \ln\left(\frac{RT_c\sqrt{2}}{\pi N_A h \mid \nu_A - \nu_B \mid}\right)$$

The association activation barrier was estimated to be at least of 15.8 kcal.mol^{-1}, considering the ^{31}P NMR spectroscopic data: the coalescence temperature T_c = 363 K, and the chemical-shift difference $|\nu_A - \nu_B|$ = 1017.33 Hz.

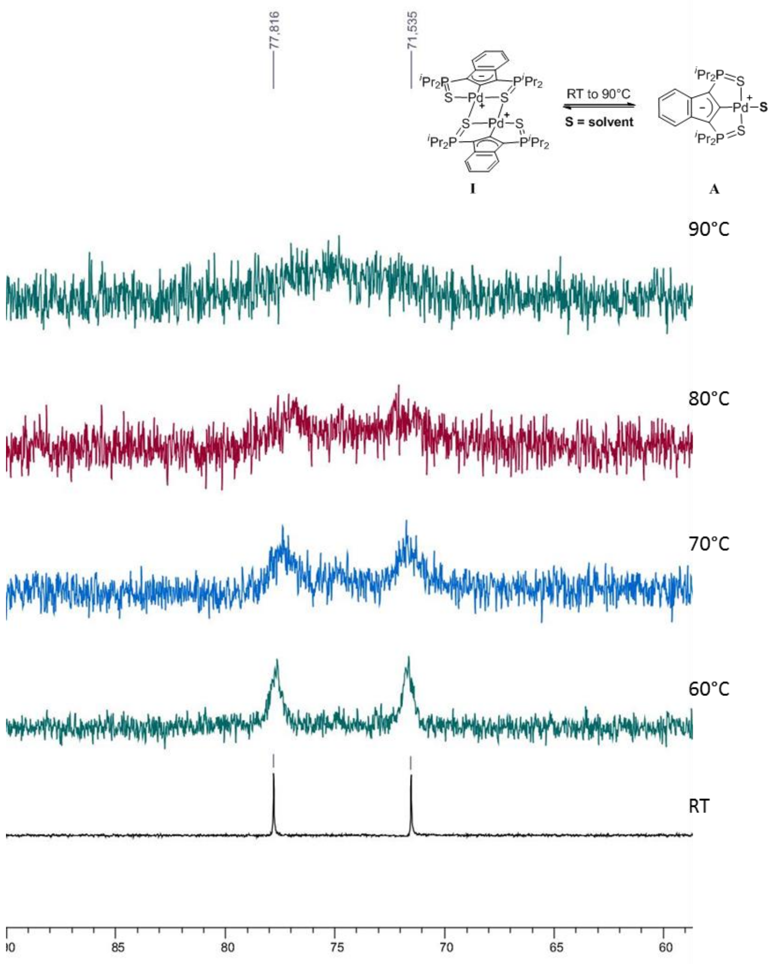

Figure S24. ^{31}P NMR analysis of Palladium indenediide dimer **I** at variable temperature in $CDCl_3$, carried out at $1.4x10^{-2}$ $mol.L^{-1}$.

VII. General procedure for cycloisomerization of alkynoic acids in presence of additives:

In a NMR pressure tube, alkynoic acid (0.098 mmol), dried additive (x mol%) and complex **I** (2.5 mg, 5 mol% [Pd]) in 0.7 mL of $CDCl_3$ were heated at the corresponding temperature under argon atmosphere. The progress of the reaction was monitored by ^1H NMR.

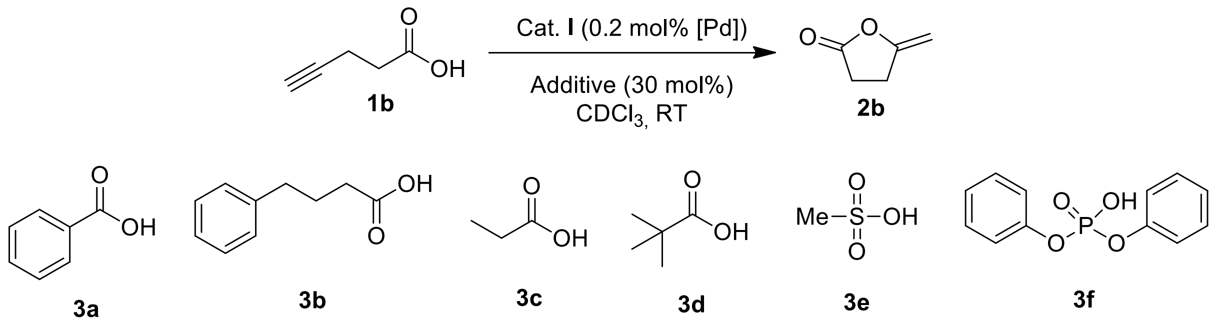

Figure S25. Additives having no impact (**3a-d**) or inhibiting (**3e,f**) the cyclisation of 4-pentynoic acid **1b**.

Table S9. Evaluation of additives 3 in the cyclisation of 4-pentynoic acid **1b**.

	Conversion (%)	
**Additives**	**1h00**	**2h00**
**/**	77%	88%
**3a**	73%	86%
**3b**	70%	86%
**3c**	72%	86%
**3d**	71%	84%
**Additives**	**30 min**	
**/**	44%	
**3e**	0%	
**3f**	6%	

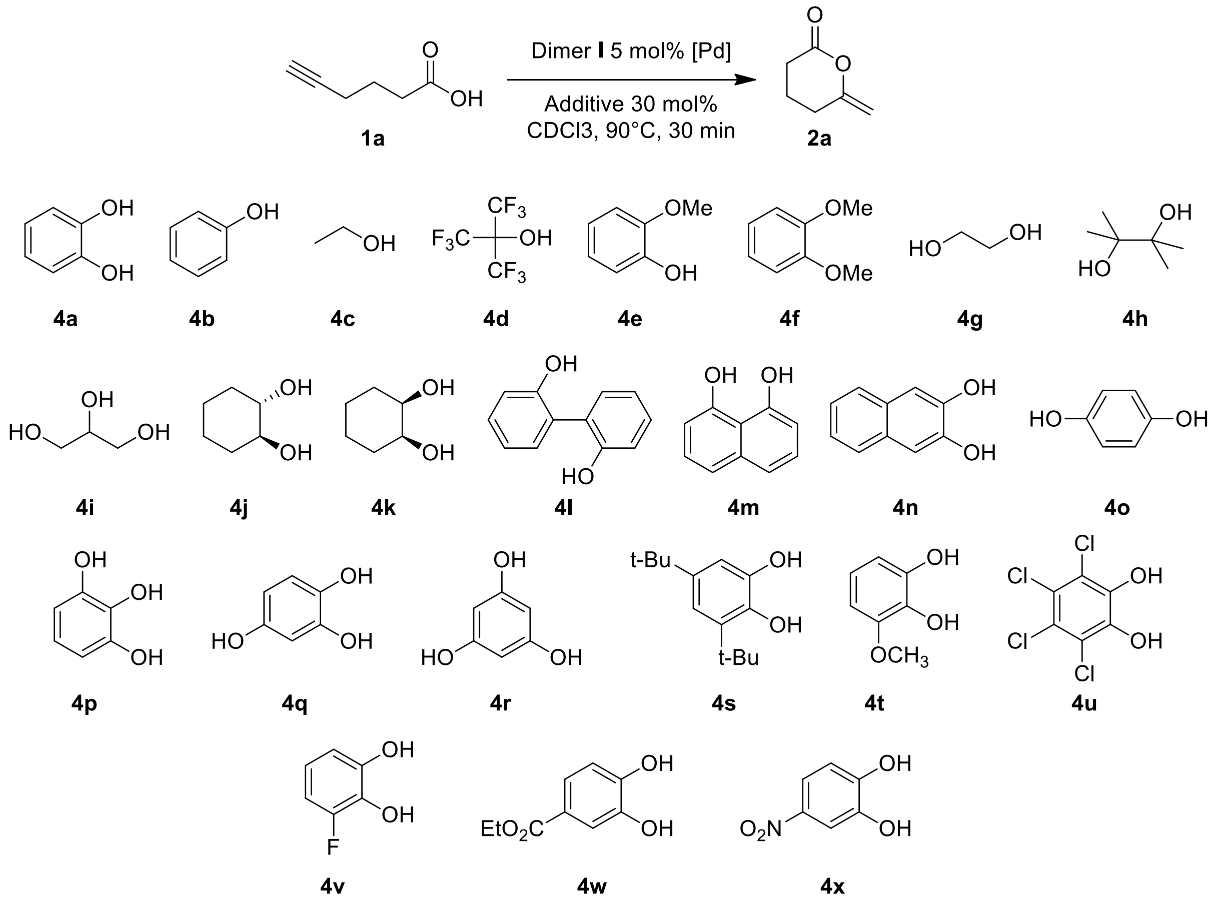

Figure S26. H-bond additives library used in the cycloisomerization of 5-hexynoic acid **1a** catalyzed by indenediide dimer **I**.

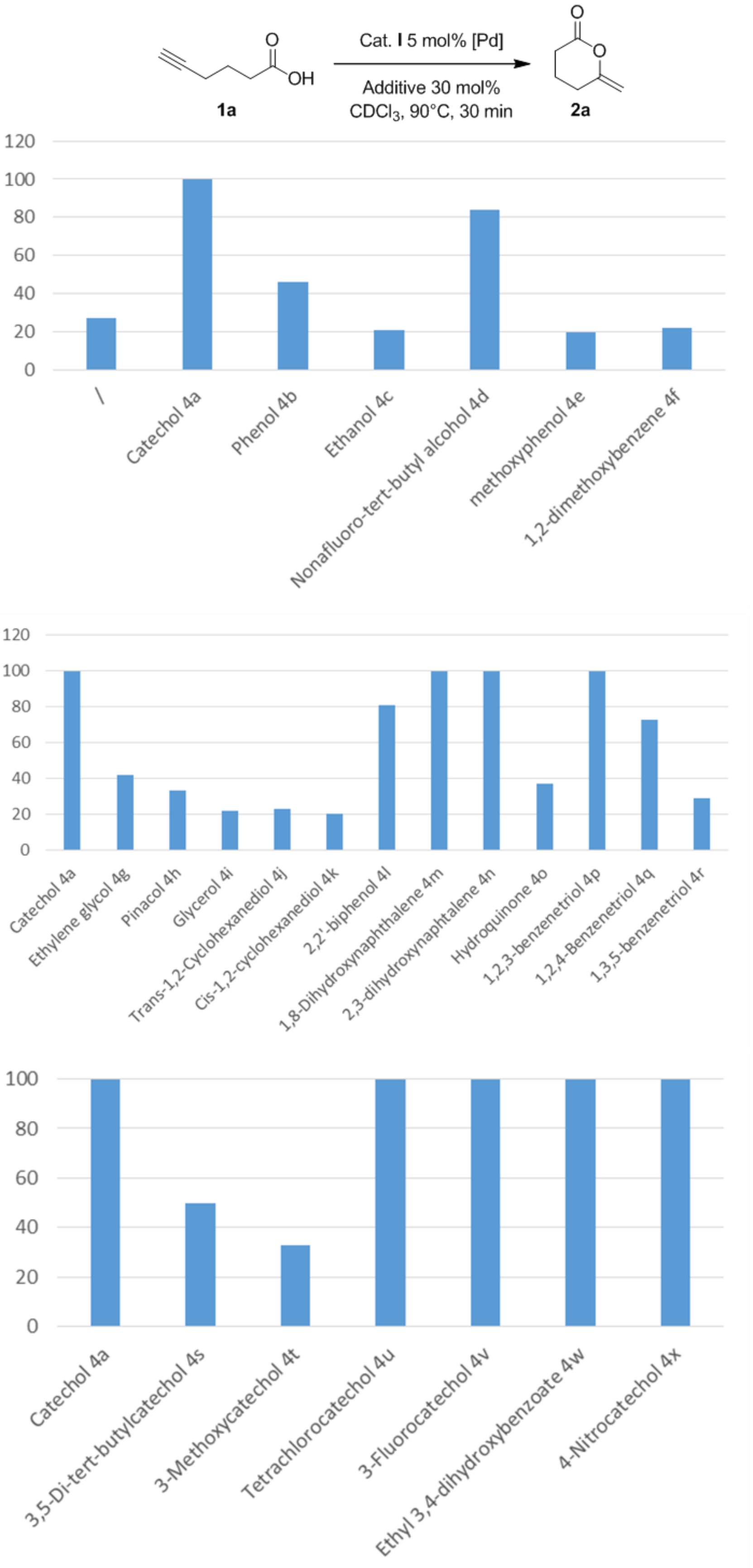

Figure S27. Evaluation of the impact of weak H-donor compounds **4** (30 %mol) on the cyclization of 5-hexynoic acid **1a**.

Table S10. Evaluation of the additives in the cyclization of 5-hexynoic acid **1a** and optimization of the reaction conditions.

Entry[a]	Additives	mol% Pd	mol% additive	T°C	conversion[b]
1	1,2,3-benzenetriol **4p**	5	5	90	89%
2	Ethyl 3,4-dihydroxybenzoate **4w**	5	5	90	88%
3	Tetrachlorocatechol **4u**	5	5	90	90%
4	4-Nitrocatechol **4x**	5	5	90	100%
5	4-Nitrocatechol **4x**	5	5	60	100%
6	4-Nitrocatechol **4x**	5	5	40	79%
7	4-Nitrocatechol **4x**	1	5	90	100%
8	4-Nitrocatechol **4x**	0.2	1	90	92%[c]

(a) Catalytic reactions performed under argon during 30 min using 0.1 mmol of 5-hexynoic acid (0.14 M in $CDCl_3$) and 5 mol% [Pd] with dimer **I**. (*b*) Conversion were determined by ^1H NMR analysis. (c) Reaction time of 36h.

VIII. Characterization of product 2f:

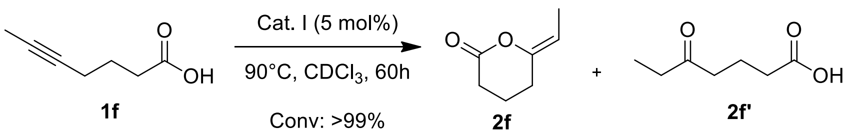

Ratio **2f**/**2f'**: 70/30

Product **2f** was obtained following the general procedure at 90°C in 60 h. Under these conditions, hydration of the alkyne moiety was observed. The residue was purified by flash chromatography. The high volatility of compound **2f** prevented accurate determination of isolated yield. A NMR yield of 70% was determined using mesitylene as internal standard. The NMR signals corresponding to the hydration product **2f'** in the crude are in total agreement with the reported characterization.[S2]

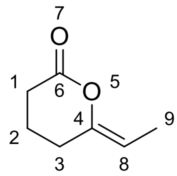

RMN-^{1}H ($CDCl_3$) : δ_{ppm} 4.62 (qt, $^3J_{HH}$ = 6.9 Hz, $^4J_{HH}$ = 0.9 Hz, 1H, H^8), 2.62 (t, $^3J_{HH}$ = 6.9 Hz, 2H, H^1), 2.39 (m, 2H, H^3), 1.85 (m, 2H, H^2), 1.63 (td, $^3J_{HH}$ = 6.9 Hz, $^5J_{HH}$ = 1.5 Hz, 3H, H^9).

RMN-^{13}C{^{1}H} ($CDCl_3$) : δ_{ppm} 168,5 (C^6), 147,98 (C^4), 103,61 (C^8), 30,65 (C^1), 27,17 (C^3), 18,89 (C^2), 9,54 (C^9).

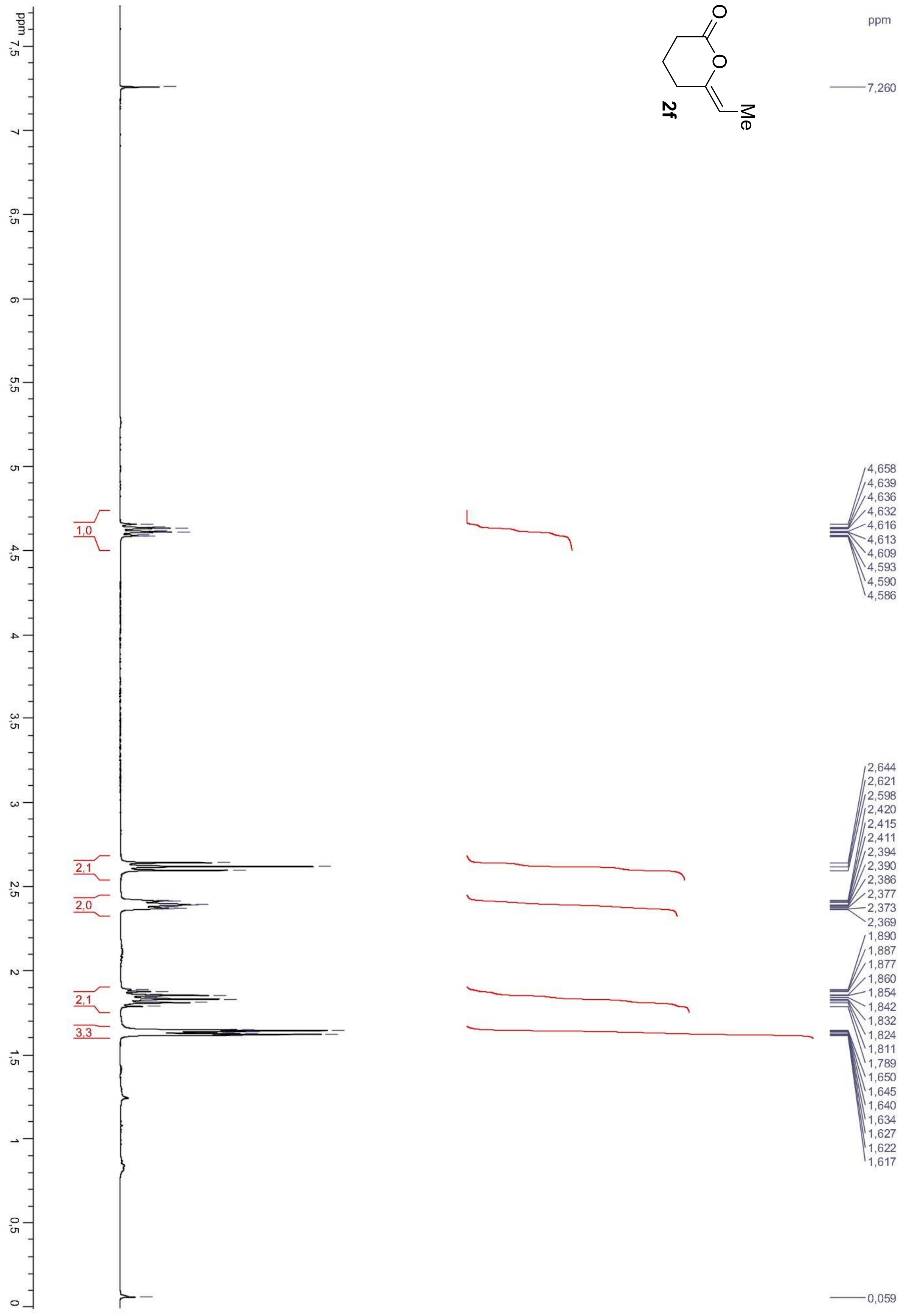
ppm
7,260
4,658
4,639
4,636
4,632
4,616
4,613
4,609
4,593
4,590
4,586
2,644
2,621
2,598
2,420
2,415
2,411
2,394
2,390
2,386
2,377
2,373
2,369
1,890
1,887
1,877
1,860
1,854
1,842
1,832
1,824
1,811
1,789
1,650
1,645
1,640
1,634
1,627
1,622
1,617
0,059
1,0
2,1
2,0
2,1
3,3
2f
Me

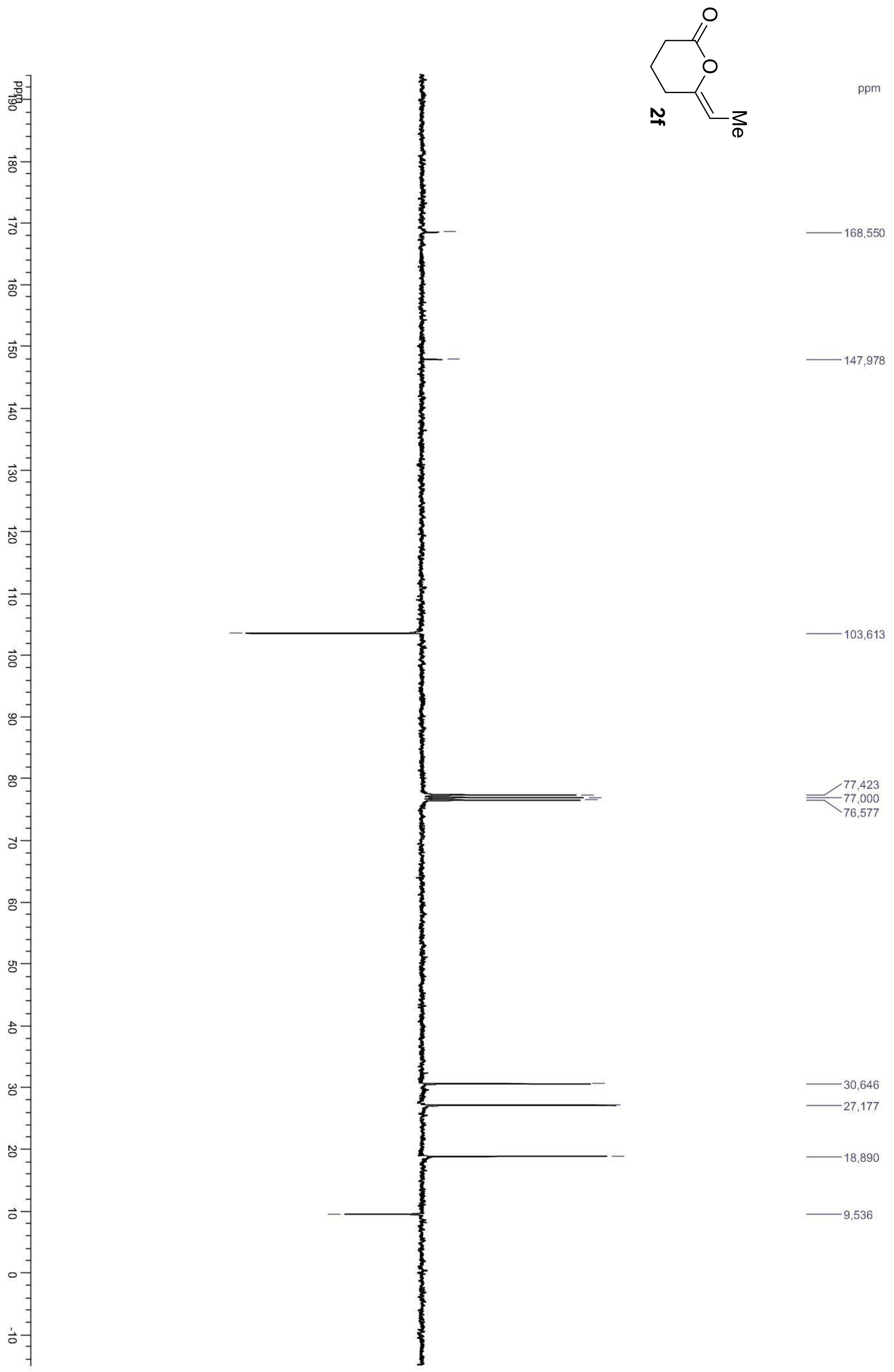

O
O
Me
2f
ppm
168,550
147,978
103,613
77,423
77,000
76,577
30,646
27,177
18,890
9,536

IX. Computational details

Calculations were carried out with the Gaussian 09 program[S3] on the real experimental palladium-pincer system at the B3PW91 level of theory.[S4] Palladium atom was treated with the corresponding Stuttgart-Dresden RECP (relativistic effective core potential) in combination with its adapted basis set,[S5] augmented by an extra set of a *f* polarization function.[S6] Phosphorus atoms were represented by the ECP from Dolg *et al.* and its associated basis set,[S7] augmented also by *d* polarization functions.[S8] For the remaining atoms the 6-31G(d,p) basis set was used.[S9] Geometry optimizations carried out without any symmetry restrictions, and were followed by analytical frequency calculations to confirm that a minimum or a transition state had been reached. The connection between the transition state and the corresponding minima were done by performing IRC calculations.[S10] Finally, the CYLview program was used for the representation of 3D structures.[S11]

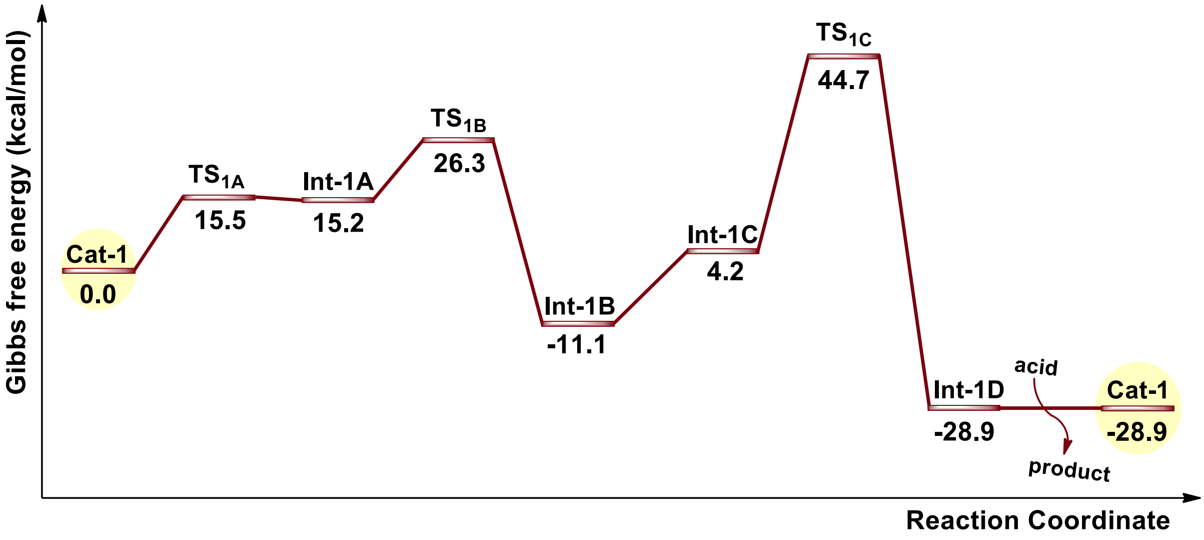

Figure S28. Gibbs free energy profile for the cyclization of 4-pentynoic acid **1b** involving one molecule of substrate per Pd center.

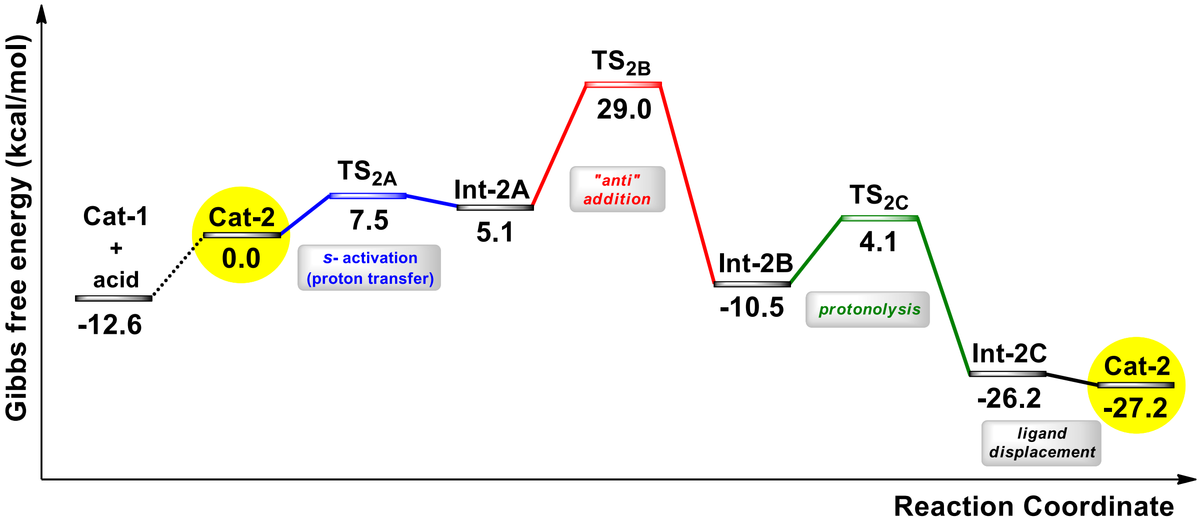

Figure S29. Gibbs free energy profile for the cyclization of 4-pentynoic acid **1b** involving two molecules of substrate per Pd center.

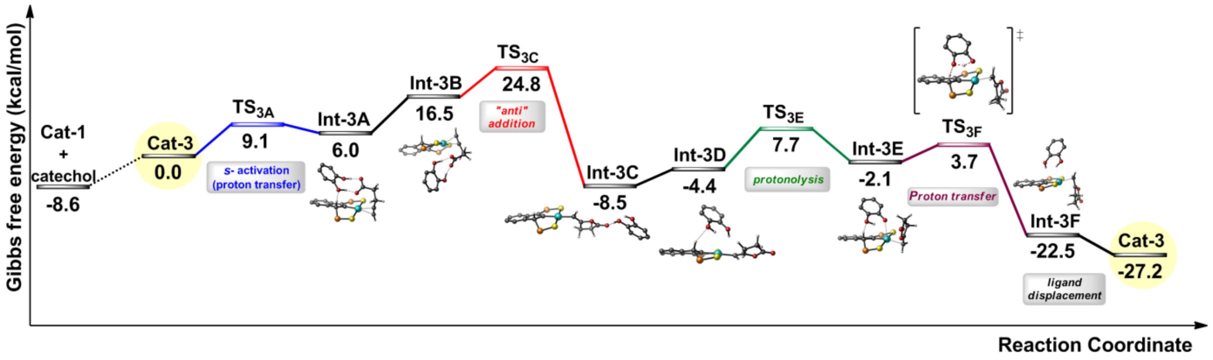

Figure S30. Gibbs free energy profile for the cyclization of 4-pentynoic acid **1b** involving catechol **4a** as additive.

Cartesian coordinates of the optimized structures along with their enthalpy and Gibbs free energies.

Cat-1
H= *-2552.762035* a.u.
G= *-2552.887446* a.u.

S	2.36229100	2.28585300	0.20849900
S	-2.35685400	2.50565400	0.11942600
C	-0.89184300	-1.72176400	-0.23776300
C	0.54350800	-1.78586400	-0.26567600
C	1.17806500	-3.02631500	-0.43093600
C	0.41207800	-4.17603000	-0.57418700
C	-0.99134000	-4.11150800	-0.56114900
C	-1.64461900	-2.89663300	-0.39677800
C	-1.24795900	-0.33716300	-0.05551600
C	-0.06949300	0.44518200	0.01415400
C	1.03048300	-0.43845700	-0.10607500
H	2.26252000	-3.09226800	-0.45567200
H	0.90406200	-5.13640400	-0.70476200
H	-1.57225500	-5.02157700	-0.68657700
H	-2.73180200	-2.85872300	-0.40578300
P	2.63935600	0.24413700	0.04037400
P	-2.76896600	0.48450800	0.21300300
C	3.75587200	-0.11644400	-1.37985800
C	4.48354000	-1.31306200	-1.42211000
C	3.82551400	0.77842000	-2.45321700
C	5.26145300	-1.61686400	-2.53710300
H	4.45630900	-2.00160900	-0.58238300
C	4.60892200	0.46873300	-3.56345200
H	3.28668400	1.72113600	-2.41791600
C	5.32368700	-0.72737100	-3.60938200
H	5.82299900	-2.54654400	-2.56421900
H	4.66212300	1.17303600	-4.38874800
H	5.93475600	-0.96414700	-4.47619800
C	3.52595100	-0.41073800	1.50946100
C	4.87783000	-0.10760400	1.71811700
C	2.82304600	-1.16262700	2.45583100
C	5.51921200	-0.55661000	2.86874900
H	5.42583800	0.47835100	0.98521200
C	3.47366600	-1.61326100	3.60397100
H	1.77581600	-1.39470100	2.28529000
C	4.81794000	-1.31052600	3.81143700
H	6.56675200	-0.31824500	3.03053100
H	2.92728300	-2.20227900	4.33516300
H	5.32194800	-1.66155700	4.70782400
C	-4.03123900	0.01141600	-1.03435600
C	-3.59270200	-0.34889600	-2.31439600
C	-5.40053100	0.04263100	-0.74819000
C	-4.52154800	-0.67015600	-3.30081000
H	-2.52749400	-0.38675100	-2.52381000
C	-6.32452100	-0.27546000	-1.74122700
H	-5.74456300	0.30677400	0.24740200
C	-5.88657200	-0.63119700	-3.01638100
H	-4.17827300	-0.95676900	-4.29083400
H	-7.38708900	-0.25070600	-1.51623400
H	-6.60933500	-0.88374100	-3.78742900
C	-3.52764600	0.09545800	1.84618900
C	-4.27027700	1.06037900	2.53564800
C	-3.35327600	-1.17873300	2.40003800
C	-4.85364500	0.74424000	3.76147600
H	-4.36806700	2.06099700	2.12358100
C	-3.93668600	-1.48588400	3.62760500
H	-2.74985500	-1.92067600	1.88526400
C	-4.69026300	-0.52871900	4.30635600
H	-5.42721500	1.49722900	4.29484900
H	-3.79399800	-2.47350200	4.05724800
H	-5.14075400	-0.77158900	5.26488000
Pd	0.01091300	2.42308800	0.16257800
H	0.54732700	2.13536700	-3.44422800
C	0.26980100	4.57183100	0.86200200
C	-0.08887600	4.74355700	-0.29984900
H	0.61115800	4.63974400	1.87350200
O	0.11829100	2.98247800	-3.24694100
C	-0.55184600	5.19310700	-1.61688500
C	1.08840600	3.90686700	-3.12892900
C	0.54271100	5.24871300	-2.69549000
H	-0.96821100	6.20021700	-1.48870500
H	-1.36984000	4.54511300	-1.94725100
O	2.25104200	3.68936200	-3.38414500
H	1.39192800	5.85558200	-2.37597600
H	0.11409100	5.72455900	-3.58655600

TS$_{1A}$
H= *-2552.742652* a.u.
G= *-2552.862727* a.u.

S	2.51315500	2.50134800	0.20648100
S	-2.20043100	2.82423400	0.33762900
C	-0.81552100	-1.19670000	-1.09227800
C	0.51656200	-1.41741500	-0.64773900

C	1.10983800	-2.67339400	-0.80238500
C	0.36708100	-3.70402700	-1.37550400
C	-0.94516800	-3.48862400	-1.80924800
C	-1.53860900	-2.23197200	-1.68143200
C	-1.13790000	0.23861700	-0.89321500
C	0.03756100	0.81108000	-0.19248100
C	1.01018000	-0.15535600	-0.10506100
H	2.13726400	-2.84642400	-0.49323600
H	0.81851000	-4.68493000	-1.49798300
H	-1.50215500	-4.30267500	-2.26500200
H	-2.54648700	-2.06041200	-2.05138700
P	2.63435600	0.44242700	0.30332800
P	-2.58893500	0.82896800	0.01409100
C	3.85450600	-0.20190200	-0.89590700
C	4.99436200	-0.90560000	-0.49188300
C	3.60008200	0.03097400	-2.25564500
C	5.88687000	-1.37919100	-1.45247500
H	5.18639600	-1.08523500	0.56151900
C	4.49965500	-0.45157800	-3.20233900
H	2.71432000	0.58136700	-2.57204100
C	5.63922600	-1.15281100	-2.80520000
H	6.77346200	-1.92457600	-1.14130800
H	4.30467900	-0.27623700	-4.25630500
H	6.33587100	-1.52395100	-3.55224300
C	3.15710300	-0.12533600	1.96608800
C	4.15101100	0.56922000	2.66586600
C	2.55902800	-1.25568500	2.53364900
C	4.55445800	0.12234100	3.92153200
H	4.59215900	1.46469300	2.23666100
C	2.96649600	-1.69501800	3.79246500
H	1.77232200	-1.78230700	2.00096400
C	3.96413500	-1.00997700	4.48411500
H	5.32407700	0.66359800	4.46442200
H	2.49776600	-2.56984700	4.23400000
H	4.27696900	-1.35311200	5.46639100
C	-4.13453100	0.59177700	-0.93330700
C	-4.14191100	0.89956200	-2.30253000
C	-5.30722700	0.16000900	-0.30193700
C	-5.32144700	0.75372800	-3.02820200
H	-3.24101100	1.26440100	-2.79024000
C	-6.48193700	0.02398900	-1.03890000
H	-5.30495100	-0.07050700	0.75874800
C	-6.48871200	0.31605200	-2.40150000
H	-5.32676600	0.98719700	-4.08906000
H	-7.39022700	-0.31099700	-0.54612100
H	-7.40495900	0.20644900	-2.97526200
C	-2.78963300	-0.07570300	1.60219800
C	-2.71772100	0.61422300	2.81603100
C	-2.97734700	-1.46594100	1.59838600
C	-2.83780800	-0.08027800	4.01880800
H	-2.56131600	1.68917100	2.80835700
C	-3.09341900	-2.15219200	2.80448400
H	-3.02139600	-2.01339300	0.66224400
C	-3.02577600	-1.46094500	4.01457700
H	-2.78181100	0.46107400	4.95894700
H	-3.23464600	-3.22926800	2.79722600
H	-3.11823200	-2.00025100	4.95337900
Pd	0.16212700	2.75327500	0.05573400
H	-1.15231700	0.94833900	-1.96950800
C	0.44678800	5.04067300	0.12250100
C	-0.01078500	4.81280400	-0.99691100
H	0.88123100	5.46192800	1.00383100
O	-1.16716100	1.95625500	-2.88655100
C	-0.60375400	4.73861600	-2.33831200
C	0.02837800	2.36784500	-3.16276400
C	0.15481200	3.88778300	-3.37328400
H	-0.68628500	5.77505500	-2.69296800
H	-1.61876200	4.33700000	-2.23917600
O	1.03125700	1.66170900	-3.27723500
H	1.21543300	4.14733400	-3.40558300
H	-0.27511400	4.12249800	-4.35492700

Int-1A

H= *-2552.740574* a.u.

G= *-2552.860501* a.u.

S	2.44169000	2.52888000	0.26599100
S	-2.26833100	2.80130700	0.30211100
C	-0.82887000	-1.25024100	-1.02206900
C	0.48155800	-1.44351700	-0.52061700
C	1.07817000	-2.70306800	-0.58672400
C	0.35733200	-3.76053200	-1.14331300
C	-0.93189500	-3.56691700	-1.64421200
C	-1.52893400	-2.30401600	-1.59670000
C	-1.16780300	0.20238400	-0.89660700
C	0.00998600	0.81446100	-0.19260400
C	0.96364100	-0.14962000	-0.02681200
H	2.09091800	-2.86135300	-0.22674200
H	0.81234600	-4.74573200	-1.19953400
H	-1.46924000	-4.40023200	-2.08803700
H	-2.51904300	-2.14664000	-2.01688900
P	2.59797200	0.47188500	0.33345100
P	-2.64575300	0.80603300	0.01065100
C	3.76103500	-0.14096900	-0.93589900
C	4.86010000	-0.94445700	-0.61148600
C	3.49316200	0.21181700	-2.26731800
C	5.70287700	-1.39655300	-1.62546800
H	5.05935500	-1.21636300	0.42057500
C	4.34331500	-0.25410300	-3.26725000
H	2.63152800	0.83126700	-2.52981600
C	5.44406800	-1.05149700	-2.95103100
H	6.55916300	-2.01753600	-1.37745400
H	4.13673200	0.01022600	-4.30019700
H	6.10184400	-1.40642800	-3.74008200
C	3.19730200	-0.10331800	1.96493900
C	4.33725500	0.47850300	2.53484400
C	2.50658000	-1.10829000	2.64981600
C	4.78916800	0.04268900	3.77705100
H	4.85980100	1.27704600	2.01515500
C	2.96479600	-1.53811300	3.89486800
H	1.61156500	-1.54602900	2.21763000
C	4.10469900	-0.96653800	4.45640400
H	5.67231700	0.49567300	4.21831200
H	2.42555800	-2.31699000	4.42635200
H	4.45796400	-1.30159300	5.42763900
C	-4.14628700	0.53756700	-0.99089300
C	-4.10006200	0.89902000	-2.34699700
C	-5.32400400	0.03437100	-0.42588600
C	-5.24037700	0.73080600	-3.12874200
H	-3.19084700	1.32047100	-2.77754400
C	-6.45722200	-0.12195900	-1.22138800
H	-5.35886200	-0.23313700	0.62562500
C	-6.41409700	0.22166300	-2.57158900
H	-5.20910200	1.00498200	-4.17925900
H	-7.37221700	-0.51130200	-0.78409900
H	-7.29874800	0.09695100	-3.19020400
C	-2.83731400	-0.09940300	1.59531000

C	-2.75067100	0.59798200	2.80416100
C	-3.04329300	-1.48708800	1.60054300
C	-2.87238500	-0.08732700	4.01177300
H	-2.58325400	1.67123900	2.78766100
C	-3.16079000	-2.16398600	2.81181100
H	-3.10240200	-2.03955300	0.66846600
C	-3.07706200	-1.46567600	4.01680100
H	-2.80516900	0.45908700	4.94813000
H	-3.31665200	-3.23899000	2.81249300
H	-3.17131000	-1.99791900	4.95941800
Pd	0.09725300	2.75235100	0.05491800
H	-1.24282600	0.75340700	-1.88554500
C	0.31681800	5.02998800	0.26157300
C	-0.01612600	4.86762800	-0.91212900
H	0.63779800	5.40612700	1.20936800
O	-1.29030600	2.22045300	-2.99573400
C	-0.42524700	4.84055900	-2.31929700
C	-0.04529800	2.42167800	-3.14584500
C	0.39284100	3.90679100	-3.22927800
H	-0.37206100	5.88044600	-2.67007900
H	-1.47108500	4.51327400	-2.37314300
O	0.84571400	1.55517900	-3.24399000
H	1.46421100	3.98112200	-3.02661300
H	0.23075400	4.23583400	-4.26324100

TS_{1B}

H= *-2552.720970* a.u.

G= *-2552.84288* a.u.

S	2.03248400	2.27368000	0.77849100
S	-2.49685500	2.54586200	-0.57387000
C	-0.98503800	-1.69500400	-0.81197500
C	0.31438800	-1.78059100	-0.25824500
C	0.91817100	-3.02815000	-0.09509300
C	0.21409900	-4.17254000	-0.47481500
C	-1.06692000	-4.08346500	-1.02254800
C	-1.67176600	-2.83544800	-1.20647600
C	-1.32693700	-0.23875600	-0.95531100
C	-0.17226200	0.51065900	-0.31574200
C	0.76648900	-0.41731000	0.04136500
H	1.91880700	-3.11363400	0.31773000
H	0.67565200	-5.14825300	-0.34916100
H	-1.59255100	-4.98663500	-1.31876400
H	-2.65929000	-2.76322100	-1.65526000
P	2.37057900	0.27877800	0.44285200
P	-2.82821500	0.56597400	-0.23686100
C	3.46290900	-0.00426200	-0.99663500
C	4.42372500	-1.02335800	-1.02981300
C	3.24658600	0.80871600	-2.11685100
C	5.17642100	-1.21440400	-2.18723400
H	4.59032400	-1.65854600	-0.16465000
C	4.00481600	0.60847200	-3.26751700
H	2.49305800	1.59595400	-2.11803700
C	4.96904900	-0.39893900	-3.30093700
H	5.92746200	-1.99938600	-2.21541900
H	3.81655600	1.26979500	-4.10828700
H	5.56457400	-0.55110000	-4.19752300
C	3.11735800	-0.51441800	1.91024900
C	4.45978600	-0.27098600	2.23063000
C	2.32256000	-1.28252600	2.76866900
C	5.00239800	-0.80626900	3.39573700
H	5.07619500	0.33663800	1.57421300
C	2.87404800	-1.81726600	3.93152200
H	1.27747900	-1.45756500	2.53050100
C	4.21167100	-1.58116900	4.24472000
H	6.04305000	-0.61566200	3.64173800
H	2.25593700	-2.41632700	4.59417100
H	4.63826800	-1.99743800	5.15307500
C	-4.32297700	-0.01899500	-1.11673000
C	-4.41583100	0.23246000	-2.49349500
C	-5.35905300	-0.68955600	-0.45790600
C	-5.53149700	-0.20012700	-3.20310900
H	-3.63014100	0.78400900	-3.00300800
C	-6.47557000	-1.11556100	-1.17622900
H	-5.29953000	-0.87478400	0.60971300
C	-6.56091400	-0.87577900	-2.54592100
H	-5.59975500	-0.00300500	-4.26886000
H	-7.27996400	-1.63231100	-0.66081300
H	-7.43245400	-1.20899300	-3.10222000
C	-2.97316100	0.10836700	1.52947500
C	-3.08414200	1.12372300	2.48444800
C	-2.96297700	-1.23619500	1.92817800
C	-3.19082900	0.79531300	3.83435200
H	-3.07163300	2.16148900	2.16347500
C	-3.06726600	-1.55368500	3.28067700
H	-2.86376800	-2.03001800	1.19458800
C	-3.18268300	-0.54034700	4.23243800
H	-3.27353500	1.58587600	4.57433800
H	-3.05542400	-2.59507000	3.58942600
H	-3.26284400	-0.79334700	5.28599100
Pd	-0.12954100	2.46752200	-0.17332100
H	-1.38016800	0.06090100	-2.01288400
C	-0.05341400	4.64016300	0.02713500
C	0.17177700	5.02083500	-1.13777000
H	-0.22801400	4.99816800	1.02690700
O	0.83193400	3.19260600	-2.56750500
C	0.34502300	5.73727700	-2.39427500
C	1.67258100	3.81321100	-3.30317300
C	1.61350100	5.34763400	-3.14454500
H	0.34005100	6.80606200	-2.12522000
H	-0.53640400	5.56266500	-3.02215900
O	2.51072800	3.33333400	-4.07724600
H	2.50464300	5.66850400	-2.59229500
H	1.65243200	5.82991400	-4.12561900

Int-1B

H= *-2552.779131* a.u.

G= *-2552.902362* a.u.

S	2.37520500	2.02922100	-0.27967600
S	-2.29138300	2.41662400	-0.86270700
C	-0.99316900	-1.93947600	-1.05854000
C	0.33855400	-2.04251600	-0.58470900
C	0.88848800	-3.30110800	-0.33552400
C	0.11014300	-4.43744200	-0.56567400
C	-1.19510300	-4.33222900	-1.04958400
C	-1.75180900	-3.07523600	-1.30969200
C	-1.28513200	-0.48345800	-1.29382500
C	-0.04902700	0.26570900	-0.83766800
C	0.86833900	-0.67929700	-0.46143700
H	1.90799800	-3.40296900	0.02385800
H	0.53219200	-5.42040800	-0.37384800
H	-1.77892400	-5.22990000	-1.23190400
H	-2.76308200	-2.99245100	-1.70047600
P	2.47152900	-0.00839400	-0.01524800
P	-2.64827800	0.45216800	-0.44339100
C	3.77518700	-0.74595000	-1.08357300
C	4.49074300	-1.88541700	-0.69649700

C	3.99908500	-0.17544600	-2.34222400
C	5.42016400	-2.45140700	-1.56739300
H	4.33582700	-2.32280400	0.28548600
C	4.92794700	-0.74752100	-3.20764400
H	3.45545200	0.72109400	-2.62779100
C	5.63787400	-1.88461600	-2.82229000
H	5.97696200	-3.33284400	-1.26187300
H	5.10078600	-0.29951800	-4.18190200
H	6.36499400	-2.32624900	-3.49812300
C	2.91360600	-0.46689300	1.70333800
C	4.18963800	-0.16358300	2.19732400
C	1.95855000	-1.04656800	2.54435300
C	4.50686500	-0.44952400	3.52163600
H	4.92908100	0.30042900	1.55031900
C	2.28431600	-1.33147200	3.87012800
H	0.96545800	-1.26809500	2.16586100
C	3.55506800	-1.03512600	4.35828900
H	5.49592100	-0.21164100	3.90259400
H	1.54050900	-1.78255300	4.52064100
H	3.80516400	-1.25605700	5.39228800
C	-4.26566800	-0.08419900	-1.11857600
C	-4.49980400	0.11736900	-2.48668200
C	-5.25983500	-0.65901000	-0.31985000
C	-5.71172800	-0.27070900	-3.04893200
H	-3.74689700	0.60046600	-3.10402800
C	-6.47318400	-1.04217700	-0.89027800
H	-5.09298200	-0.80179400	0.74281900
C	-6.69830400	-0.85334500	-2.25199100
H	-5.88864400	-0.11011800	-4.10835700
H	-7.24371200	-1.48463000	-0.26546500
H	-7.64526200	-1.15200400	-2.69270900
C	-2.61562700	0.05653600	1.34710200
C	-2.52697500	1.10629400	2.26678300
C	-2.67461500	-1.27026800	1.79824200
C	-2.50556500	0.83059900	3.63270600
H	-2.46320400	2.12822300	1.90356600
C	-2.65091200	-1.53565200	3.16608800
H	-2.72933600	-2.09235200	1.09185600
C	-2.56888800	-0.48698500	4.08262400
H	-2.43449700	1.64856600	4.34361000
H	-2.69614800	-2.56398200	3.51350800
H	-2.55266400	-0.69849700	5.14833000
Pd	0.06129200	2.26666300	-0.65653500
H	-1.47805800	-0.26315100	-2.35374400
C	0.20916800	4.29898800	-0.44195200
C	0.17791400	5.21281100	-1.40709900
H	0.34693300	4.70905900	0.56215000
O	0.00494800	4.83889700	-2.76007700
C	0.29245600	6.72291500	-1.37162400
C	0.29709700	5.85857200	-3.60219700
C	0.75689700	7.05819100	-2.78741300
H	0.97233100	7.06018000	-0.58621200
H	-0.69004400	7.17450200	-1.17953800
O	0.20626200	5.76130800	-4.79983000
H	1.85022200	7.10520500	-2.86217000
H	0.35554300	7.98061600	-3.21154700

Int-1C

H= *-2552.754988* a.u.

G= *-2552.87797* a.u.

S	2.21822900	1.54451600	0.84370300
S	-2.95015700	2.06423800	-0.98710800
C	-1.21859600	-2.17924200	-0.52001300
C	-0.02022800	-2.30526800	0.22084800
C	0.29414500	-3.50715800	0.85777000
C	-0.58179100	-4.58641800	0.72710100
C	-1.74855200	-4.47340900	-0.03024000
C	-2.07540700	-3.26585000	-0.65733500
C	-1.30958100	-0.77214600	-1.05718400
C	0.01624200	-0.14379000	-0.68670700
C	0.69629000	-1.03638500	0.10396700
H	1.20208600	-3.60693300	1.44545600
H	-0.34534200	-5.52884700	1.21397700
H	-2.40877700	-5.32927300	-0.13802300
H	-2.98608500	-3.18661300	-1.24198500
P	2.27487600	-0.48456200	0.76823300
P	-2.75051200	0.22900200	-0.28477300
C	3.63879300	-1.12208400	-0.28350600
C	3.54492500	-2.39660600	-0.85674000
C	4.77064600	-0.33259900	-0.50783800
C	4.58859500	-2.87863400	-1.64348000
H	2.65842200	-3.00555000	-0.70315400
C	5.81116800	-0.82323400	-1.29479100
H	4.81897200	0.66686400	-0.08438300
C	5.72157800	-2.09421900	-1.86072700
H	4.51202800	-3.86423300	-2.09382200
H	6.68735900	-0.20635100	-1.47251600
H	6.53137400	-2.47136600	-2.47916800
C	2.51229100	-1.22037900	2.43073200
C	3.64923800	-1.96597000	2.75832300
C	1.52252800	-0.98875200	3.39494800
C	3.79251200	-2.48163500	4.04616400
H	4.41959500	-2.14270800	2.01411700
C	1.67224600	-1.50933900	4.67648100
H	0.64482200	-0.39937900	3.14465000
C	2.80572800	-2.25530700	5.00344300
H	4.67787800	-3.05786600	4.29944800
H	0.90267000	-1.32938400	5.42133300
H	2.92048600	-2.65720100	6.00638600
C	-4.24998100	-0.77654000	-0.66830700
C	-4.52094300	-1.07085800	-2.01196800
C	-5.17528000	-1.14422200	0.31276400
C	-5.68245900	-1.75267700	-2.36244000
H	-3.83456100	-0.74760500	-2.79009900
C	-6.34200000	-1.82115500	-0.04333800
H	-4.99410700	-0.89658700	1.35328400
C	-6.59426300	-2.13381900	-1.37666400
H	-5.88057200	-1.97849800	-3.40651300
H	-7.05532100	-2.10087800	0.72696500
H	-7.50286800	-2.66260300	-1.65073000
C	-2.49751000	0.14734600	1.54159900
C	-2.27636700	1.35069100	2.21881000
C	-2.53578200	-1.05710000	2.25954100
C	-2.10165700	1.35392700	3.60158300
H	-2.25167500	2.27653300	1.65061200
C	-2.36949400	-1.04576500	3.64355700
H	-2.70160400	-2.00004000	1.74927800
C	-2.15526700	0.15786200	4.31595500
H	-1.93058200	2.29356800	4.11886300
H	-2.41060500	-1.98103200	4.19491300
H	-2.03539200	0.16341200	5.39624800
Pd	0.83258900	1.54131500	-1.18766700
H	-1.51397300	-0.71477800	-2.13129700
C	-0.08640100	1.68707000	-2.93968000
C	0.38745200	2.78550300	-3.51456200
H	-0.86122200	1.09603500	-3.41591300

O	1.38728000	3.46374400	-2.74945400
C	0.14592500	3.58876300	-4.76316900
C	1.55717600	4.74960000	-3.18198900
C	0.60735500	4.98831500	-4.34504700
H	-0.89925800	3.54448000	-5.07619200
H	0.76253800	3.21373000	-5.59043200
O	2.33593400	5.50594800	-2.67143400
H	-0.22256100	5.59474300	-3.96362200
H	1.10978000	5.56475400	-5.12384100

TS_{1C}
H= *-2552.692413* a.u.
G= *-2552.813544* a.u.

S	2.87532400	1.45482000	-0.90640900
S	-1.77137600	2.35321300	-0.28693900
C	-0.83223700	-1.96912300	-0.97601300
C	0.46431600	-2.23140500	-0.45420900
C	0.90637800	-3.54624800	-0.30546300
C	0.05320700	-4.59067800	-0.66690500
C	-1.21892700	-4.33339000	-1.18233600
C	-1.66608200	-3.01895500	-1.34547700
C	-1.01196900	-0.49036500	-1.08026600
C	0.25259100	0.07635400	-0.51587600
C	1.11254800	-0.94080200	-0.20186200
H	1.90198300	-3.76000300	0.07334600
H	0.38940200	-5.61788900	-0.55433600
H	-1.86162100	-5.16033700	-1.47113600
H	-2.64685800	-2.82045000	-1.77049900
P	2.79509800	-0.38483300	0.00530500
P	-2.37927900	0.40988100	-0.24945400
C	3.93989400	-1.56108800	-0.80822800
C	4.88118300	-2.30045800	-0.08500000
C	3.83650800	-1.71751400	-2.19707500
C	5.71491400	-3.19795500	-0.75162100
H	4.96653000	-2.17480200	0.98995800
C	4.67012700	-2.61751200	-2.85292000
H	3.11549100	-1.12803500	-2.75680800
C	5.60870500	-3.35752200	-2.13162800
H	6.44922500	-3.76864800	-0.19029100
H	4.58937600	-2.73859000	-3.92917100
H	6.26065500	-4.05649500	-2.64817300
C	3.25550700	-0.33094500	1.77694800
C	4.23346400	0.56764200	2.21706700
C	2.62892800	-1.19201300	2.68503500
C	4.59227800	0.59196900	3.56261700
H	4.69072500	1.25765900	1.51324500
C	2.99392700	-1.16112900	4.02972300
H	1.85033100	-1.87014800	2.34670800
C	3.97542900	-0.27240700	4.46733100
H	5.34668100	1.29397700	3.90577600
H	2.50362200	-1.82496200	4.73599500
H	4.25315100	-0.24598600	5.51736000
C	-3.92705400	0.15533500	-1.18559600
C	-3.88189800	0.40897300	-2.56500300
C	-5.12447800	-0.23072300	-0.57428300
C	-5.03712800	0.25201100	-3.32503300
H	-2.95022500	0.74304900	-3.02496000
C	-6.27568300	-0.37883400	-1.34666100
H	-5.16196500	-0.41181300	0.49528700
C	-6.23137200	-0.14277700	-2.71938100
H	-5.00508200	0.44505500	-4.39350800
H	-7.20675900	-0.67634900	-0.87269000
H	-7.13051800	-0.25984000	-3.31822600
C	-2.62654000	-0.22879400	1.45491900
C	-2.41438800	0.62915600	2.53846200
C	-2.99156700	-1.56445300	1.68097600
C	-2.57278200	0.15702900	3.84039600
H	-2.12015400	1.65808600	2.35039300
C	-3.14458100	-2.02852400	2.98497900
H	-3.14765700	-2.24265200	0.84853300
C	-2.93757900	-1.16897000	4.06441900
H	-2.40805100	0.82832500	4.67830800
H	-3.42443200	-3.06405400	3.15660700
H	-3.06003200	-1.53533600	5.08008000
Pd	0.53041900	1.90412400	-0.93709600
H	-0.99655300	-0.00178000	-2.14797500
C	-0.59407800	1.31630600	-3.62123400
C	-0.10844300	2.52940900	-3.83485600
H	-0.85570700	0.87723500	-4.59765300
O	0.36987700	3.34347800	-2.67609100
C	0.16329300	3.43839600	-5.01433500
C	0.51829900	4.65277900	-2.97886000
C	0.11473300	4.85023600	-4.42706800
H	-0.56150300	3.27266600	-5.81495900
H	1.16341700	3.24402300	-5.42622400
O	0.89879900	5.46756900	-2.17568400
H	-0.90485000	5.25567500	-4.42617700
H	0.76574500	5.58551100	-4.90457700

Int-1D
H= *-2552.80502* a.u.
G= *-2552.930732* a.u.

S	2.52963500	0.85954200	-0.55791500
S	-2.06554600	1.76098800	0.09011500
C	-1.08592200	-2.52485800	0.97480100
C	0.33023600	-2.76064500	0.90837000
C	0.83369000	-4.04482900	1.16663300
C	-0.04345800	-5.07259500	1.49391300
C	-1.42603200	-4.84001200	1.56735200
C	-1.94905600	-3.57830900	1.30909700
C	-1.30006500	-1.13506100	0.65476700
C	-0.05211100	-0.52452600	0.37878400
C	0.94955200	-1.51083400	0.54221200
H	1.90104100	-4.24377400	1.09761200
H	0.34555900	-6.06839100	1.69062600
H	-2.09558200	-5.65683100	1.82454600
H	-3.02270000	-3.41597000	1.36505300
P	2.60792700	-0.99807400	0.33244800
P	-2.73588200	-0.14730300	0.49279400
C	3.54718900	-2.18224800	-0.71167400
C	4.91384400	-2.41673900	-0.52713500
C	2.85929000	-2.83283600	-1.74361400
C	5.58903500	-3.29165800	-1.37623200
H	5.44793500	-1.92469700	0.28029800
C	3.53917400	-3.70843800	-2.58570900
H	1.79511000	-2.65545000	-1.87145000
C	4.90356700	-3.93708000	-2.40435300
H	6.65032300	-3.47267100	-1.22998000
H	3.00125700	-4.21709500	-3.38077800
H	5.43142500	-4.62267900	-3.06177300
C	3.54162500	-0.89723900	1.91708900
C	4.54730500	0.05858300	2.09570600
C	3.23076000	-1.78686800	2.95234600
C	5.25377600	0.10797100	3.29629500
H	4.75429400	0.77501800	1.30538800
C	3.93919700	-1.72940700	4.15093000

H	2.42642900	-2.50649900	2.82897600
C	4.95297900	-0.78708900	4.32228200
H	6.03196900	0.85382900	3.43323600
H	3.69124700	-2.41691500	4.95481000
H	5.50061400	-0.74277700	5.25982000
C	-3.86676100	-0.72477100	-0.84545400
C	-3.66665400	-0.26596000	-2.15203300
C	-4.85742500	-1.68075900	-0.59034500
C	-4.45070000	-0.76002000	-3.19136000
H	-2.90277800	0.48317500	-2.34139500
C	-5.63847000	-2.17240700	-1.63490000
H	-5.02875500	-2.03555900	0.42173800
C	-5.43647200	-1.71322100	-2.93525200
H	-4.29358000	-0.39678200	-4.20330000
H	-6.40654900	-2.91299100	-1.42985400
H	-6.04818200	-2.09576200	-3.74779500
C	-3.76277500	-0.14663300	2.01374300
C	-5.03561600	0.43847800	2.01463100
C	-3.23278100	-0.66981400	3.19730600
C	-5.77229600	0.49576800	3.19421700
H	-5.44756600	0.84996400	1.09718700
C	-3.97863700	-0.61380300	4.37416000
H	-2.24373600	-1.11846500	3.18834400
C	-5.24491300	-0.03189400	4.37396700
H	-6.75794200	0.95286900	3.19367100
H	-3.56707300	-1.02580300	5.29122300
H	-5.82270800	0.01229000	5.29322400
Pd	0.23467000	1.31562100	-0.19479500
H	0.21198600	2.23304400	-5.45375800
C	0.19775100	3.31269600	-5.36491500
C	0.21077600	3.90319900	-4.17805600
H	0.16762300	3.91209200	-6.26681200
O	0.24388700	3.13163600	-3.01370500
C	0.18525600	5.36047300	-3.78923400
C	0.48497700	3.89430600	-1.93472900
C	0.68560800	5.33377800	-2.34287700
H	-0.84232400	5.73927200	-3.83551700
H	0.79580800	5.96991800	-4.45794200
O	0.54416000	3.43609600	-0.81150400
H	0.16068800	5.99848300	-1.65504700
H	1.75669400	5.55237200	-2.26253400

Cat-2

H= *-2897.074699* a.u.

G= *-2897.074699* a.u.

S	2.20368800	1.95437800	0.85535500
S	-2.39037800	2.20794400	-0.21245600
C	-1.17749000	-1.96567800	0.89474800
C	0.24443300	-2.07200300	1.07675900
C	0.81633500	-3.32393800	1.35368400
C	0.00179800	-4.44397300	1.44762000
C	-1.38811400	-4.34127900	1.26138900
C	-1.97921800	-3.11630900	0.98395100
C	-1.46397000	-0.58294000	0.60994300
C	-0.25526700	0.15962100	0.62049700
C	0.79477500	-0.75102500	0.90487800
H	1.88970900	-3.42192200	1.49229000
H	0.44364200	-5.41313900	1.66380700
H	-2.00591400	-5.23277100	1.33083600
H	-3.05343900	-3.05461500	0.82454300
P	2.41044900	-0.08525900	1.11391600
P	-2.94628300	0.34807000	0.48310000
Pd	-0.08956000	2.12111700	0.35399900
H	0.05373700	-0.77580800	-1.57537100
H	0.98960300	1.35979900	-3.24408000
C	0.73507500	-2.60101800	-4.15452000
H	-0.26309000	-2.75511200	-4.58186900
C	0.68367500	-1.35794500	-3.30056600
O	1.19327500	-0.30071300	-3.63214500
O	0.01370600	-1.55274900	-2.17033300
H	1.42136200	-2.40703000	-4.98035300
C	-4.16820100	-0.43867400	-0.63572900
C	-5.54221300	-0.22249100	-0.47967700
C	-3.70003100	-1.23763500	-1.68611000
C	-6.44131300	-0.79510900	-1.37631800
H	-5.90954200	0.38440200	0.34261400
C	-4.60612300	-1.81179700	-2.57458700
H	-2.63444200	-1.41632000	-1.79621500
C	-5.97456600	-1.58943100	-2.42300000
H	-7.50715200	-0.62506100	-1.25307200
H	-4.24218400	-2.43810000	-3.38414100
H	-6.67812500	-2.03952900	-3.11799200
C	-3.80641900	0.51666800	2.10407700
C	-4.48041800	1.69637300	2.43682800
C	-3.77820600	-0.55216700	3.00810300
C	-5.13986700	1.79771200	3.66108200
H	-4.46528700	2.53662400	1.74800100
C	-4.43621200	-0.44156800	4.23111300
H	-3.22945300	-1.45760900	2.76594400
C	-5.12042300	0.72953400	4.55669900
H	-5.66034900	2.71622700	3.91770900
H	-4.40714600	-1.26990600	4.93346200
H	-5.62981800	0.81281400	5.51286200
C	3.65913800	-0.75104100	-0.06313200
C	4.30212800	-1.96773100	0.20481000
C	3.90785200	-0.07945300	-1.26516200
C	5.17847100	-2.51236000	-0.73076800
H	4.13311700	-2.48280100	1.14616600
C	4.78769600	-0.63154700	-2.19518200
H	3.43236200	0.87341300	-1.48040000
C	5.42056000	-1.84537900	-1.93255200
H	5.67630600	-3.45426800	-0.51749400
H	4.97311200	-0.10056200	-3.12398700
H	6.10783800	-2.27025700	-2.65929400
C	3.07726100	-0.45423700	2.78292800
C	4.42472600	-0.20748600	3.07759900
C	2.21392300	-0.90944800	3.78474500
C	4.90199200	-0.41848400	4.36800700
H	5.09721700	0.14911300	2.30224000
C	2.70071000	-1.12354600	5.07333700
H	1.17093600	-1.09978500	3.55024800
C	4.04108200	-0.87808900	5.36579000
H	5.94647200	-0.22418300	4.59496700
H	2.02984900	-1.48394200	5.84801400
H	4.41712900	-1.04446800	6.37156800
C	1.14857500	-3.86062400	-3.36849600
H	2.13825900	-3.70438700	-2.92143700
H	0.45579500	-4.01406300	-2.53403900
C	1.18015600	-5.04549100	-4.21766800
C	1.20187600	-6.01650800	-4.93693800
H	1.22201700	-6.87913400	-5.56291300
C	0.10469900	4.33056300	0.78617100
C	0.05992600	4.37646400	-0.44013900
C	-0.01341200	4.69698300	-1.86811700
C	1.30990000	4.52239800	-2.62970300
H	-0.33341000	5.74437000	-1.94093100

H	-0.79120400	4.08741100	-2.33801600
C	1.73313600	3.08531900	-2.86670900
H	2.13415800	5.03373400	-2.12862400
H	1.19244400	4.98383600	-3.61843000
O	0.68967000	2.29223300	-3.10426500
O	2.89252200	2.72722100	-2.88246300
H	0.18104700	4.51682400	1.83690200

TS_{2A}

H= *-2897.066515* a.u.

G= *-2897.066515* a.u.

S	2.06470500	2.19315100	0.89311900
S	-2.45192500	2.08748300	-0.50653000
C	-0.93180100	-2.03878800	0.38711900
C	0.34774100	-1.97242200	0.99984900
C	0.94869000	-3.13349300	1.49873100
C	0.26664500	-4.34290500	1.39671300
C	-0.99227200	-4.40935100	0.78677700
C	-1.58968200	-3.26299700	0.26421300
C	-1.25427200	-0.69514500	-0.15031800
C	-0.17760900	0.19868100	0.32683200
C	0.77552100	-0.58080600	0.95300100
H	1.93402300	-3.09951700	1.95472100
H	0.72254700	-5.24955000	1.78532900
H	-1.50185500	-5.36577400	0.70785100
H	-2.55116300	-3.32478700	-0.23922200
P	2.29587500	0.21480500	1.41909900
P	-2.80272600	0.17540600	0.15693100
Pd	-0.15942300	2.16746200	0.10357800
H	-1.10212100	-0.84436700	-1.48388800
H	0.86737700	0.92999000	-2.92888100
C	0.21916200	-2.47664600	-4.32202500
H	-0.08967200	-2.06293100	-5.29042700
C	0.07706900	-1.36752700	-3.28728200
O	1.02952900	-0.58334500	-3.13527600
O	-1.04828000	-1.32462400	-2.66343700
H	1.28186500	-2.71438500	-4.41427700
C	-4.25774100	-0.55532000	-0.67703100
C	-5.51669200	-0.47754700	-0.06652500
C	-4.12897600	-1.12871900	-1.94976100
C	-6.63936600	-0.97588300	-0.72269200
H	-5.62044000	-0.03233200	0.91803200
C	-5.25884400	-1.62994200	-2.59302900
H	-3.15879300	-1.18987300	-2.43447500
C	-6.51111500	-1.55465200	-1.98443700
H	-7.61318400	-0.91323700	-0.24550900
H	-5.15669600	-2.08013400	-3.57631800
H	-7.38742900	-1.94650100	-2.49350100
C	-3.20546900	0.15979900	1.95535800
C	-3.32277300	1.35747100	2.66615000
C	-3.37796600	-1.06451800	2.61716700
C	-3.60958600	1.33249300	4.03058600
H	-3.18627300	2.30032500	2.14413500
C	-3.66062000	-1.08115600	3.98079400
H	-3.28432500	-2.00134600	2.07699200
C	-3.77763300	0.11573900	4.68843700
H	-3.70137900	2.26669200	4.57747900
H	-3.79013500	-2.03204700	4.48995500
H	-4.00128400	0.09823800	5.75164100
C	3.73437100	-0.53714000	0.57005800
C	4.51130700	-1.51751200	1.20160600
C	3.99705200	-0.17085900	-0.75607800
C	5.55058900	-2.13019000	0.50528900
H	4.31470900	-1.79677700	2.23251500
C	5.03880100	-0.79218800	-1.44112200
H	3.40974100	0.59338700	-1.25901500
C	5.81375600	-1.76782200	-0.81533700
H	6.15453300	-2.88797200	0.99651600
H	5.23765000	-0.50434800	-2.46885500
H	6.62583000	-2.24624000	-1.35622300
C	2.60201600	0.05269100	3.21755200
C	3.79737400	0.53786700	3.76550400
C	1.61864100	-0.48895000	4.05154700
C	4.00633600	0.47242200	5.13961400
H	4.55925600	0.96761000	3.12083800
C	1.83718500	-0.55340200	5.42738100
H	0.68961400	-0.85741800	3.62742200
C	3.02740200	-0.07478500	5.97094300
H	4.93282000	0.85028800	5.56245000
H	1.07362200	-0.97811300	6.07273500
H	3.19386800	-0.12514300	7.04342500
C	-0.60791200	-3.73150000	-4.01042600
H	-0.30342700	-4.13917200	-3.03843800
H	-1.65975000	-3.44686100	-3.89868000
C	-0.47884200	-4.76279300	-5.03435100
C	-0.36440600	-5.60799900	-5.89158100
H	-0.26438200	-6.35539400	-6.64472300
C	-0.17764500	4.41659400	0.42790600
C	-0.04648500	4.36732500	-0.79208300
C	0.08794300	4.53877700	-2.24075900
C	1.46382500	4.15584100	-2.80596500
H	-0.11200800	5.59758100	-2.45130200
H	-0.68891600	3.95297800	-2.74206900
C	1.76690400	2.66394900	-2.83317500
H	2.27325300	4.65023400	-2.26390800
H	1.51338600	4.50828000	-3.84378500
O	0.66978800	1.93142100	-2.91888400
O	2.90666800	2.23980600	-2.81376400
H	-0.26071900	4.68404000	1.45989400

Int-2A

H= *-2897.06966* a.u.

G= *-2897.06966* a.u.

S	2.05323500	2.27355300	0.92734200
S	-2.48827600	2.16216800	-0.38766200
C	-0.89546100	-1.98290000	0.29426000
C	0.33428900	-1.90370600	0.98788800
C	0.92385700	-3.05797100	1.50580400
C	0.27458300	-4.27992800	1.32908600
C	-0.93454000	-4.35807800	0.63349200
C	-1.52207500	-3.20821800	0.09888400
C	-1.23392200	-0.61625300	-0.21878800
C	-0.16126300	0.28636400	0.33594700
C	0.74748500	-0.49722500	0.99214900
H	1.87469000	-3.01447700	2.02845900
H	0.72290100	-5.18575500	1.72787200
H	-1.41565300	-5.32212500	0.49543100
H	-2.44790800	-3.27388000	-0.46612700
P	2.26992100	0.30310500	1.48453600
P	-2.80754000	0.22720600	0.20524500
Pd	-0.16292900	2.24390200	0.11461100
H	-1.19387200	-0.59415600	-1.34443700
H	0.81174400	0.75887300	-3.11871400
C	0.23304100	-2.54905100	-4.39651400
H	0.29843300	-2.25470900	-5.45100600
C	-0.01522300	-1.28221100	-3.57502800

O	0.99283100	-0.51813300	-3.46306100
O	-1.15673000	-1.08647000	-3.09408800
H	1.21926100	-2.94280800	-4.13353600
C	-4.23475000	-0.52499400	-0.64242100
C	-5.49397800	-0.51050200	-0.02728700
C	-4.07782800	-1.05148700	-1.93325100
C	-6.59700500	-1.02858800	-0.70040100
H	-5.61312500	-0.09992800	0.97077700
C	-5.19166000	-1.57224900	-2.58995500
H	-3.10723700	-1.06276700	-2.42758800
C	-6.44552100	-1.56150900	-1.98019900
H	-7.57297000	-1.01634200	-0.22381200
H	-5.07285200	-1.98618000	-3.58696400
H	-7.30697400	-1.96806400	-2.50290100
C	-3.11011200	0.08652200	2.00991300
C	-3.07997500	1.22761900	2.81667200
C	-3.35145700	-1.17113600	2.58148100
C	-3.28951700	1.11229600	4.19010100
H	-2.89285900	2.19602600	2.36137400
C	-3.55595000	-1.27775000	3.95485400
H	-3.37695300	-2.06259000	1.96283400
C	-3.52597300	-0.13767600	4.75893300
H	-3.26803700	2.00153800	4.81349200
H	-3.74117200	-2.25303900	4.39572600
H	-3.69068300	-0.22513000	5.82939100
C	3.68615900	-0.49198900	0.64540700
C	4.48214700	-1.43590600	1.30873600
C	3.90767000	-0.19514100	-0.70601700
C	5.50272200	-2.08314000	0.61655400
H	4.31399800	-1.66028800	2.35791400
C	4.93021600	-0.85382100	-1.38534300
H	3.31019700	0.54198600	-1.23999900
C	5.72502800	-1.79299500	-0.72960400
H	6.12343900	-2.81200500	1.13007000
H	5.09515000	-0.62176100	-2.43280400
H	6.52155900	-2.30030700	-1.26724000
C	2.54244600	0.15052100	3.28695400
C	3.72285800	0.65266400	3.85205000
C	1.55326200	-0.40272800	4.10668600
C	3.91178200	0.59080300	5.22919900
H	4.48835000	1.09279100	3.21879100
C	1.75178200	-0.46252000	5.48570300
H	0.63379500	-0.78300200	3.67208500
C	2.92763000	0.03174600	6.04627500
H	4.82678400	0.98099900	5.66536900
H	0.98388100	-0.89562400	6.12014400
H	3.07854500	-0.01533200	7.12112400
C	-0.85469400	-3.61283100	-4.21347200
H	-0.89861800	-3.90791500	-3.15761300
H	-1.82834700	-3.16183600	-4.43610600
C	-0.65168200	-4.79649400	-5.04229400
C	-0.47502200	-5.77344800	-5.73344300
H	-0.31922500	-6.63275500	-6.34404800
C	-0.19592500	4.50452300	0.35527000
C	-0.03906000	4.39977400	-0.85876100
C	0.14076700	4.48407500	-2.30894500
C	1.52520500	4.04155600	-2.80489500
H	-0.03968800	5.53244600	-2.58175200
H	-0.62552300	3.87565800	-2.80015000
C	1.75823500	2.52978900	-2.82940100
H	2.32821000	4.50371900	-2.22567400
H	1.63781400	4.39012000	-3.83901200
O	0.65426700	1.84095200	-2.94927400
O	2.89661000	2.08471300	-2.78020000
H	-0.30327900	4.82524900	1.36951400

TS_{2B}

H= *-2897.030426* a.u.

G= *-2897.030426* a.u.

S	2.92179600	1.53188600	0.67056500
S	-1.67624800	2.04933500	-0.19678500
C	-0.58161200	-2.25960700	0.39192800
C	0.61153100	-2.28956900	1.15276500
C	1.04179900	-3.48525100	1.72860700
C	0.27705500	-4.63745600	1.53708000
C	-0.89154900	-4.60876400	0.77383300
C	-1.32443500	-3.41537900	0.18712400
C	-0.76248800	-0.86848100	-0.14334400
C	0.40325900	-0.07716600	0.41298200
C	1.18478000	-0.93887400	1.13397400
H	1.96134400	-3.52991500	2.30445100
H	0.60477500	-5.57427700	1.97928700
H	-1.46164700	-5.52080300	0.62353500
H	-2.22243000	-3.39702300	-0.42521700
P	2.75928300	-0.26742200	1.65126400
P	-2.23767500	0.15229600	0.30650000
Pd	0.69577400	1.78088200	-0.10934100
H	-0.73248900	-0.80331100	-1.24598900
H	1.03750000	1.76157000	-4.49651400
C	0.66825000	-1.52671000	-4.74498100
H	0.38788300	-1.50591300	-5.80416400
C	0.43422800	-0.13133900	-4.18128000
O	1.19798600	0.74744200	-4.76020300
O	-0.39546300	0.08691100	-3.29544600
H	1.74360700	-1.73126700	-4.72957300
C	-3.68146700	-0.39079400	-0.66702400
C	-4.92271000	-0.64420800	-0.07291600
C	-3.52472900	-0.49020800	-2.05901100
C	-6.00525600	-1.01397500	-0.86913700
H	-5.04772800	-0.54852300	1.00090400
C	-4.61435800	-0.86292100	-2.84091600
H	-2.57043800	-0.26288000	-2.53253400
C	-5.85075800	-1.12692100	-2.24934200
H	-6.96948400	-1.20841000	-0.40856500
H	-4.49694500	-0.93844300	-3.91787200
H	-6.69674600	-1.41367200	-2.86764900
C	-2.60470700	-0.04844100	2.09098300
C	-2.48132000	1.05977100	2.93539200
C	-2.98597500	-1.29252400	2.61591600
C	-2.74306100	0.92569000	4.29784300
H	-2.17726400	2.01609900	2.51910700
C	-3.24425500	-1.41674200	3.97879100
H	-3.07618900	-2.16068800	1.97144200
C	-3.12522400	-0.30901100	4.81896800
H	-2.64770800	1.78926900	4.94946700
H	-3.53910700	-2.38079100	4.38306200
H	-3.33246200	-0.41006700	5.88079100
C	4.12093700	-1.39602300	1.16726700
C	4.72494700	-2.25504700	2.09288600
C	4.51958900	-1.41643900	-0.17521900
C	5.72067000	-3.13500000	1.67193000
H	4.42868300	-2.23219200	3.13733400
C	5.51274700	-2.30043500	-0.58706200
H	4.06336500	-0.73143200	-0.88446400
C	6.11266600	-3.15912400	0.33453600
H	6.19260600	-3.79767100	2.39167500

H	5.82194000	-2.31332100	-1.62797600
H	6.89078600	-3.84428900	0.01017900
C	2.84060800	-0.10036000	3.47120700
C	3.96350100	0.49682300	4.06004700
C	1.77333700	-0.52799800	4.26707200
C	4.01701200	0.65508700	5.44119300
H	4.78582000	0.84571900	3.44129000
C	1.83623700	-0.36698700	5.65100100
H	0.89739500	-0.97730600	3.80916700
C	2.95452400	0.22210400	6.23685600
H	4.88548200	1.12166100	5.89683000
H	1.00659900	-0.69899100	6.26830600
H	2.99871600	0.34978600	7.31478800
C	-0.10868500	-2.61959500	-4.00643000
H	0.20265300	-2.64688700	-2.95480200
H	-1.17396300	-2.36048800	-3.99673400
C	0.07074000	-3.94121200	-4.59822500
C	0.22559100	-5.03174300	-5.09683600
H	0.36105600	-5.99201200	-5.53892200
C	1.47781000	3.42425500	-1.33967000
C	0.49395400	3.62547900	-2.09864800
C	-0.82349800	4.09233000	-2.53709600
C	-0.68870200	4.81812700	-3.88242400
H	-1.24827200	4.74009800	-1.76046500
H	-1.48800600	3.22966700	-2.66236700
C	0.03990400	3.86579100	-4.84045300
H	-0.10061500	5.73682900	-3.76795400
H	-1.66789400	5.09148400	-4.28257200
O	0.91134900	3.14827700	-4.18971100
O	-0.21503300	3.82283200	-6.03492200
H	2.54271700	3.34918900	-1.48371400

Int-2B

H= *-2897.091838* a.u.

G= *-2897.091838* a.u.

S	2.36031000	1.20532700	-0.22355800
S	-2.10835400	0.98297800	-1.68648100
C	-1.04490300	-2.65576200	0.83573800
C	0.16813900	-2.39454900	1.52071500
C	0.58942800	-3.25272400	2.53764400
C	-0.19809100	-4.36036800	2.85699000
C	-1.38272100	-4.62541400	2.16730800
C	-1.80762900	-3.77604200	1.14056000
C	-1.20185800	-1.61496900	-0.23648200
C	-0.03739500	-0.66714800	-0.06155000
C	0.74540100	-1.17670000	0.93897300
H	1.51853000	-3.07457500	3.07046800
H	0.12284700	-5.03276000	3.64812700
H	-1.97374700	-5.49997700	2.42376000
H	-2.72044600	-3.99163200	0.59071100
P	2.26596100	-0.26796000	1.21852900
P	-2.65451600	-0.47187600	-0.34949900
Pd	0.15356900	1.09576000	-1.02500300
H	-1.15133000	-2.03949200	-1.25363800
H	0.44160700	-0.32735600	-2.90315700
C	0.41616300	-2.28663900	-5.46731300
H	0.20886600	-1.49179600	-6.19246000
C	0.04180800	-1.77942600	-4.09398000
O	0.72476400	-0.68320900	-3.77882900
O	-0.78048000	-2.31809500	-3.37240000
H	1.50119400	-2.43518400	-5.49485500
C	-4.12668600	-1.39031400	-0.92494200
C	-5.36785200	-1.25227300	-0.29362700
C	-4.00004100	-2.20497800	-2.06024200
C	-6.47662600	-1.93622800	-0.78906800
H	-5.47033900	-0.61334100	0.57768200
C	-5.11339300	-2.88695300	-2.54289600
H	-3.04587600	-2.29550900	-2.57355300
C	-6.34970700	-2.75521300	-1.90908100
H	-7.43953000	-1.82524900	-0.29892000
H	-5.01488900	-3.51910900	-3.42055900
H	-7.21544400	-3.28764400	-2.29288600
C	-3.02675400	0.20356800	1.31499000
C	-3.00863100	1.58808600	1.51062100
C	-3.32611800	-0.65344100	2.38453300
C	-3.29262600	2.11382600	2.76995800
H	-2.76588400	2.24100400	0.67692300
C	-3.60506900	-0.11865000	3.64028500
H	-3.33727300	-1.72944700	2.24384600
C	-3.59056400	1.26334400	3.83301700
H	-3.27893200	3.18975500	2.91741300
H	-3.83577400	-0.78404900	4.46737800
H	-3.81370400	1.67625500	4.81304600
C	3.72303900	-1.38198700	1.09947000
C	4.23950400	-2.03117000	2.22771500
C	4.28175400	-1.62236100	-0.16118300
C	5.30305300	-2.92102900	2.09124000
H	3.82468700	-1.83287400	3.21168200
C	5.34486500	-2.51283900	-0.28955200
H	3.89128000	-1.09803700	-1.02904800
C	5.85485800	-3.16293000	0.83394500
H	5.70369500	-3.42001400	2.96905300
H	5.77922100	-2.69293800	-1.26863300
H	6.68680500	-3.85397000	0.73071300
C	2.29850400	0.41949800	2.91642000
C	3.45918200	1.04410200	3.39334000
C	1.14651500	0.38516400	3.70892000
C	3.46544500	1.62096000	4.65947900
H	4.35199700	1.08375400	2.77529400
C	1.16127000	0.96482300	4.97723900
H	0.24349500	-0.08760300	3.33497500
C	2.31727300	1.58024800	5.45269300
H	4.36545700	2.10586000	5.02645900
H	0.26550600	0.93541600	5.59080200
H	2.32512100	2.03238200	6.44055500
C	-0.32625900	-3.57434600	-5.83566300
H	-0.12322400	-4.34116100	-5.07876700
H	-1.40685400	-3.39435100	-5.79432100
C	0.04359100	-4.07005900	-7.15657600
C	0.35934500	-4.46950800	-8.25259800
H	0.63368500	-4.82489500	-9.21948600
C	0.37308600	2.86057600	-2.05100900
C	0.52372100	4.08009800	-1.54027100
C	0.58182700	4.56678000	-0.11163200
C	1.29846400	5.90908600	-0.23812000
H	1.08553000	3.84346700	0.53239900
H	-0.43508100	4.71245500	0.27649700
C	1.06458700	6.30299800	-1.68782100
H	2.38169600	5.81149200	-0.09557400
H	0.94751300	6.69855300	0.42932300
O	0.64940500	5.21281800	-2.38071400
O	1.22209300	7.37967700	-2.20447200
H	0.34950800	2.80764600	-3.14291100

TS_{2C}

H= *-2897.070453* a.u.

G= *-2897.070453* a.u.

S	2.92637900	1.21326800	-1.19209100
S	-1.77604800	1.91314400	-1.07671700
C	-0.66710100	-2.45392100	-0.89918200
C	0.63028600	-2.53074500	-0.33490000
C	1.12720600	-3.75877200	0.10365500
C	0.32442300	-4.89503900	-0.02067500
C	-0.94781500	-4.81919600	-0.58927200
C	-1.44730200	-3.59326500	-1.04264000
C	-0.90213800	-1.03588600	-1.33795300
C	0.34557400	-0.28476100	-0.93658500
C	1.21228200	-1.18567700	-0.38189200
H	2.12835400	-3.83994200	0.51779600
H	0.70377600	-5.85535300	0.31813300
H	-1.54928300	-5.71786000	-0.69161200
H	-2.42868200	-3.53826800	-1.50779100
P	2.83833600	-0.50619700	-0.07924700
P	-2.29019400	-0.01463200	-0.66246400
C	4.12151400	-1.70019500	-0.61371700
C	5.05480500	-2.23965500	0.27710500
C	4.14151100	-2.07194200	-1.96498900
C	6.00279100	-3.15292700	-0.18384200
H	5.04569200	-1.94680400	1.32246000
C	5.08864600	-2.98566500	-2.41547100
H	3.42643600	-1.63605800	-2.65741700
C	6.01886800	-3.52644500	-1.52596400
H	6.73041600	-3.56811900	0.50765600
H	5.10359200	-3.27265000	-3.46281200
H	6.75977900	-4.23676000	-1.88217600
C	3.09649100	-0.21553600	1.71239200
C	3.89717900	0.85020400	2.13576800
C	2.49094400	-1.05888900	2.65180600
C	4.10436700	1.05958500	3.49754700
H	4.33172300	1.52217400	1.40084100
C	2.70263300	-0.84186100	4.01180600
H	1.84629300	-1.87047200	2.32599500
C	3.51061500	0.21370200	4.43408500
H	4.72181600	1.89054900	3.82606800
H	2.22851200	-1.49260600	4.74102200
H	3.66965400	0.38329200	5.49539400
C	-3.82920200	-0.48637900	-1.52892100
C	-3.80655700	-0.45143200	-2.93219800
C	-5.00419900	-0.82478500	-0.85034400
C	-4.95713100	-0.77755700	-3.64348600
H	-2.90354000	-0.15063700	-3.46136000
C	-6.15198400	-1.14489800	-1.57460100
H	-5.02755500	-0.83423500	0.23475800
C	-6.12776200	-1.12626700	-2.96741700
H	-4.94050800	-0.75265000	-4.72914600
H	-7.06512800	-1.40508000	-1.04701200
H	-7.02370300	-1.37653600	-3.52867600
C	-2.49041000	-0.33692500	1.13164300
C	-2.28696600	0.71500800	2.03057300
C	-2.82734200	-1.61211700	1.60972600
C	-2.42595500	0.49494800	3.39968000
H	-2.01608900	1.69529700	1.64838300
C	-2.96422300	-1.82249700	2.97953800
H	-2.97368700	-2.43896900	0.92286400
C	-2.76574000	-0.77038900	3.87408900
H	-2.26608200	1.31498700	4.09360900
H	-3.22401000	-2.81109600	3.34706700
H	-2.87521900	-0.93929400	4.94185200
Pd	0.58570300	1.60166000	-1.39016800
H	-1.00767200	-0.91466800	-2.42774000
C	1.03786500	3.63565000	-2.06930400
C	0.29860900	4.75192500	-1.91266500
H	2.10489900	3.82089400	-1.93717400
O	0.88970600	5.95031600	-1.55066800
C	-1.16910100	4.99289100	-2.15677000
C	0.03667100	7.01565100	-1.73117200
C	-1.24594600	6.50505100	-2.35521000
H	-1.74838600	4.68596900	-1.27765500
H	-1.51794400	4.40056200	-3.00455300
O	0.35049100	8.13392900	-1.43482300
H	-2.10778100	6.99415500	-1.89804000
H	-1.23045700	6.78487000	-3.41511400
H	0.62338500	2.99789400	-3.16461300
C	-0.73631000	2.13767000	-9.66136800
C	-0.33343300	2.22795600	-8.52437800
H	-1.08475300	2.06575200	-10.66586700
C	0.13646800	2.32265400	-7.14623700
H	1.19721300	2.04501500	-7.10191200
H	0.08649100	3.36023800	-6.79916800
C	-0.66532900	1.44048200	-6.17984200
H	-1.72803700	1.70846100	-6.24189300
H	-0.59372000	0.38560100	-6.45869600
C	-0.24511000	1.57822300	-4.71204000
O	0.26420800	2.71826400	-4.40508200
O	-0.44850200	0.61992600	-3.94842500

Int-2C

H= *-2897.118492* a.u.

G= *-2897.118492* a.u.

S	2.36613400	1.31065000	0.99998500
S	-2.28262400	1.80858300	0.30489200
C	-1.15190100	-2.47438000	0.90227900
C	0.27466500	-2.64517100	0.92981800
C	0.82066500	-3.93729400	0.99402500
C	-0.02768300	-5.03492200	1.03627500
C	-1.42382300	-4.86836900	1.00661400
C	-1.98886500	-3.60240600	0.93580000
C	-1.40551600	-1.06006400	0.81619300
C	-0.17326200	-0.36033700	0.79639000
C	0.85983100	-1.32988200	0.87447100
H	1.89846400	-4.08099200	1.00506100
H	0.39142500	-6.03649400	1.08560600
H	-2.06827100	-5.74322500	1.03166700
H	-3.06963300	-3.48826100	0.89445900
P	2.50863100	-0.75381300	1.02397200
P	-2.84939900	-0.07096700	0.93966300
C	3.61933000	-1.33974700	-0.32007200
C	4.49476600	-2.41604400	-0.13386200
C	3.50813000	-0.73908800	-1.58048300
C	5.25932900	-2.88318000	-1.20243800
H	4.58625100	-2.88773400	0.83980600
C	4.27566400	-1.21174300	-2.64117400
H	2.81943000	0.08748800	-1.73279400
C	5.15267600	-2.28096300	-2.45432600
H	5.93873400	-3.71763700	-1.05286800
H	4.18600200	-0.74200600	-3.61653800
H	5.75125600	-2.64516000	-3.28482200
C	3.29558400	-1.28700900	2.59300300
C	4.62987700	-0.95232400	2.86209100
C	2.53759300	-1.96549900	3.55221600
C	5.20082600	-1.30368200	4.08154600
H	5.21859100	-0.41465900	2.12365800

C	3.11824500	-2.31725200	4.77048700
H	1.50270400	-2.21695600	3.34114800
C	4.44580500	-1.98818200	5.03560100
H	6.23426900	-1.04075200	4.28899300
H	2.52875500	-2.84866700	5.51214600
H	4.89400700	-2.26177800	5.98682800
C	-4.23409900	-0.71141400	-0.07701400
C	-3.94076800	-1.23043400	-1.34501800
C	-5.55827900	-0.66614900	0.37272800
C	-4.97265500	-1.69948400	-2.15317100
H	-2.91261400	-1.26422400	-1.69305400
C	-6.58516300	-1.13432600	-0.44498000
H	-5.78772100	-0.27237600	1.35813000
C	-6.29348300	-1.65133200	-1.70586000
H	-4.74395100	-2.10615500	-3.13418000
H	-7.61238400	-1.09765500	-0.09342700
H	-7.09528200	-2.01898200	-2.34037500
C	-3.48985600	-0.00194300	2.66446300
C	-4.08905400	1.16193900	3.15766800
C	-3.36831300	-1.12933100	3.48587100
C	-4.57750100	1.19162700	4.46296100
H	-4.15163700	2.04416100	2.52647000
C	-3.85718800	-1.09102400	4.79007500
H	-2.87970800	-2.02553400	3.11392300
C	-4.46351100	0.06629400	5.27835500
H	-5.03941900	2.09795500	4.84453800
H	-3.75654300	-1.96503900	5.42744400
H	-4.83958400	0.09372600	6.29740900
Pd	0.04854500	1.61272000	0.68725100
H	-0.28912600	-0.42285100	-1.52051800
C	0.17156500	3.86502800	1.16373300
C	0.40662700	4.01155300	-0.16994900
H	-0.82029900	4.03907100	1.56607800
O	-0.62620000	4.24993900	-1.03511400
C	1.72000800	4.16888300	-0.90070800
C	-0.18027300	4.28544100	-2.35309100
C	1.31117700	4.05552000	-2.36574900
H	2.45787400	3.42956300	-0.58633000
H	2.11564300	5.16640500	-0.67225400
O	-0.94452800	4.46212200	-3.25558600
H	1.47169300	3.04690700	-2.76154200
H	1.79128100	4.77131500	-3.03564300
H	1.01338200	3.88015000	1.84766800
C	-1.17052300	0.93683700	-8.07728200
C	-0.71744000	1.07416000	-6.96528200
H	-1.56835600	0.82586400	-9.05994400
C	-0.17793500	1.22325700	-5.61850700
H	0.91670200	1.17164200	-5.64669600
H	-0.42592000	2.21755700	-5.22753600
C	-0.70769500	0.15222800	-4.65936900
H	-1.79267700	0.23173800	-4.53975700
H	-0.52040300	-0.85267900	-5.05683500
C	-0.06615700	0.23602400	-3.29490100
O	0.89627400	0.93301200	-3.02787600
O	-0.66869100	-0.56158100	-2.41427000

Cat-3

H= *-2935.218762* a.u.

G= *-2935.360207* a.u.

S	2.27805300	1.94539200	0.65951700
S	-2.41067700	2.39694800	0.30336100
C	-1.20969100	-1.87797900	0.97346200
C	0.21890500	-2.02656600	1.02520200
C	0.78271900	-3.30482300	1.16367900
C	-0.04968500	-4.41350700	1.23666100
C	-1.44642000	-4.27247100	1.16061400
C	-2.02919500	-3.01894500	1.02503800
C	-1.48441900	-0.46877700	0.84218500
C	-0.26881400	0.24691600	0.78788900
C	0.78705200	-0.70391600	0.88421300
H	1.86163700	-3.43131700	1.19474500
H	0.38432300	-5.40433600	1.33998300
H	-2.07780000	-5.15607000	1.20223900
H	-3.11035500	-2.92650400	0.95016300
P	2.40993100	-0.06325200	1.12902600
P	-2.97092900	0.46292400	0.75892600
Pd	-0.06067400	2.19849100	0.48930200
H	0.56653700	-0.84529400	-1.28516900
H	0.03481800	0.83416800	-2.69102000
C	-4.11138600	-0.22095000	-0.50613000
C	-5.49969100	-0.22826000	-0.33451700
C	-3.54916000	-0.72128300	-1.68786600
C	-6.32018900	-0.72802400	-1.34441000
H	-5.93960400	0.14688700	0.58453700
C	-4.37475100	-1.22054800	-2.69084000
H	-2.47053300	-0.72459400	-1.81412300
C	-5.75979500	-1.22277700	-2.52083900
H	-7.39798400	-0.73404100	-1.20823500
H	-3.93404000	-1.61342700	-3.60247300
H	-6.40189700	-1.61506100	-3.30466300
C	-3.90856200	0.45732200	2.34112300
C	-4.73016400	1.53978600	2.67699300
C	-3.79472300	-0.63081300	3.21376800
C	-5.45059500	1.52151900	3.86936700
H	-4.78493400	2.40223500	2.01833900
C	-4.51462600	-0.63925900	4.40728800
H	-3.13566500	-1.45865400	2.97012100
C	-5.34527100	0.43185500	4.73354600
H	-6.08542500	2.36437600	4.12788900
H	-4.41934500	-1.48252400	5.08553500
H	-5.90273100	0.42256200	5.66612600
C	3.68992000	-0.87379900	0.09375900
C	4.44632900	-1.94283700	0.59125300
C	3.84097200	-0.46887600	-1.23794000
C	5.34173200	-2.60664100	-0.24511500
H	4.34217200	-2.25395500	1.62659600
C	4.73302500	-1.14256400	-2.06851900
H	3.26852400	0.36986200	-1.62277500
C	5.48216100	-2.20988300	-1.57422300
H	5.92746700	-3.43495000	0.14356100
H	4.82703600	-0.83603700	-3.10562000
H	6.17595200	-2.73363700	-2.22596700
C	2.96712300	-0.28139500	2.86494200
C	4.23992500	0.15775500	3.25380600
C	2.09788800	-0.83368100	3.81002300
C	4.63742500	0.04039200	4.58204100
H	4.91471800	0.59454900	2.52244400
C	2.50427500	-0.94969200	5.13942100
H	1.11336500	-1.17278900	3.50226700
C	3.76983300	-0.51362700	5.52542000
H	5.62330000	0.38385900	4.88245300
H	1.82841200	-1.38177700	5.87191800
H	4.08266900	-0.60342400	6.56209300
C	0.28126800	4.42920800	0.68720000
C	0.08700500	4.32576300	-0.52155700
C	-0.16172800	4.47563200	-1.95919900

C	0.94316700	3.92865000	-2.87581200
H	-0.27711400	5.55049200	-2.14861000
H	-1.11640400	3.99969500	-2.20500200
C	1.02906200	2.42099100	-2.97388500
H	1.93029200	4.29541300	-2.58594400
H	0.75590900	4.29503900	-3.89327900
O	-0.12844600	1.81231100	-2.77650900
O	2.06852300	1.84457500	-3.25819000
H	0.49572100	4.73126100	1.69076200
C	1.02418700	-4.11551800	-4.57034500
C	1.63279200	-2.91773400	-4.93458400
C	1.48863300	-1.77278200	-4.14501200
C	0.69886700	-1.85853500	-2.98554900
C	0.08999900	-3.05400400	-2.61948300
C	0.25850000	-4.19155800	-3.40782800
H	1.15591300	-4.99364200	-5.19656100
H	2.24216900	-2.84155700	-5.83002500
H	-0.21145400	-5.12471700	-3.11293900
H	-0.50562000	-3.08830000	-1.71103200
O	0.53340300	-0.69587600	-2.25288400
O	2.11359700	-0.64158300	-4.53277400
H	2.00012400	0.09167300	-3.89376200

TS_{3B}

H= *-2935.207844* a.u.

G= *-2935.345743* a.u.

S	2.50176700	1.98255100	0.60502100
S	-2.17120500	2.33478300	-0.08958300
C	-0.85573800	-1.93844000	0.18346700
C	0.42143700	-1.98822200	0.80213500
C	0.96649800	-3.21487100	1.19565700
C	0.22277300	-4.37541900	0.99661100
C	-1.03780100	-4.32655400	0.39013200
C	-1.57558600	-3.11240200	-0.03498500
C	-1.12525500	-0.53417500	-0.22379100
C	0.01533600	0.25274100	0.30735600
C	0.92272100	-0.62160600	0.86266100
H	1.95891800	-3.26894100	1.63538400
H	0.63488900	-5.33387000	1.30041900
H	-1.59263700	-5.24626000	0.22763000
H	-2.53467500	-3.08549300	-0.54556900
P	2.45272300	0.09134800	1.42020800
P	-2.59798000	0.36385400	0.32435600
Pd	0.18162300	2.21386700	0.14308900
H	-1.06600800	-0.44531700	-1.52544800
H	-0.71839900	0.97468600	-3.30264300
C	-4.13265300	-0.14915500	-0.52319500
C	-5.33797200	-0.26482400	0.18035000
C	-4.10134800	-0.35477500	-1.90993600
C	-6.50738500	-0.59619400	-0.50075400
H	-5.36509700	-0.09878400	1.25253700
C	-5.27695300	-0.68770600	-2.57808900
H	-3.16468600	-0.25948800	-2.45672100
C	-6.47733800	-0.80950400	-1.87781800
H	-7.44117500	-0.68642700	0.04682900
H	-5.25320600	-0.84990600	-3.65192100
H	-7.39066300	-1.06848800	-2.40643300
C	-2.88306900	0.11719400	2.12581700
C	-2.84315100	1.20860500	2.99839300
C	-3.11978100	-1.17088900	2.62825700
C	-3.04204500	1.01531900	4.36451800
H	-2.65332900	2.20074700	2.59909400
C	-3.31526700	-1.35540800	3.99483300
H	-3.14313800	-2.02709100	1.96186000
C	-3.27798300	-0.26418200	4.86328800
H	-3.01137300	1.86751900	5.03748400
H	-3.49612500	-2.35486700	4.38015600
H	-3.43333700	-0.41288600	5.92841300
C	3.88230900	-0.91110800	0.88231000
C	4.94892100	-1.18450400	1.74685700
C	3.89957000	-1.37822700	-0.43964500
C	6.03685600	-1.92388900	1.28744900
H	4.93104100	-0.82709100	2.77194600
C	4.99246200	-2.11748200	-0.88418400
H	3.07491000	-1.17910900	-1.12163800
C	6.05882000	-2.38920100	-0.02647600
H	6.86493900	-2.13626600	1.95774700
H	5.00395500	-2.47995500	-1.90765800
H	6.90807000	-2.96628300	-0.38230800
C	2.51167500	0.15843400	3.25424000
C	3.23611900	1.17003000	3.89480700
C	1.83539800	-0.80654400	4.00967300
C	3.29785500	1.20356100	5.28634800
H	3.73111200	1.93604300	3.30408800
C	1.89807200	-0.76271000	5.40124100
H	1.25190200	-1.57876100	3.51691000
C	2.63092400	0.23735600	6.03929700
H	3.86045600	1.98982100	5.78175300
H	1.36732000	-1.50835800	5.98624900
H	2.67572800	0.26890600	7.12442400
C	0.51985700	4.45347200	0.38460900
C	0.22937900	4.38988600	-0.80706300
C	-0.13683100	4.56717400	-2.21604200
C	0.79976700	3.88440100	-3.22965800
H	-0.14936400	5.64905100	-2.39998200
H	-1.15669600	4.19761000	-2.36934100
C	0.63121100	2.37592600	-3.26177700
H	1.84698000	4.11292400	-3.02465400
H	0.54747400	4.27083200	-4.22367500
O	-0.60419800	2.00259500	-3.47865700
O	1.58546400	1.62138400	-3.10423900
H	0.82694000	4.72256300	1.37283300
C	0.25839600	-3.91578800	-4.65003600
C	1.19284800	-3.05829600	-4.07274300
C	0.79833700	-1.85617600	-3.48292500
C	-0.57294900	-1.50667500	-3.45664800
C	-1.49332300	-2.37225900	-4.05871200
C	-1.09076000	-3.56824000	-4.65140500
H	0.58870400	-4.84549000	-5.10566300
H	2.25238100	-3.29872000	-4.07363500
H	-1.82869300	-4.22224300	-5.10850100
H	-2.54273000	-2.09027900	-4.04241100
O	-0.98404200	-0.36872300	-2.84306900
O	1.75126400	-1.07097500	-2.92247500
H	1.49149400	-0.12084700	-2.93577100

INT-3A

H= *-2935.211043* a.u.

G= *-2935.350669* a.u.

S	2.41036200	1.90073500	0.41351500
S	-2.30012700	2.23148300	-0.09819500
C	-0.94610300	-2.04603700	0.23929700
C	0.31991000	-2.05342800	0.86930300
C	0.86974500	-3.25307900	1.32297400
C	0.14093000	-4.43154200	1.15440100
C	-1.10529600	-4.42280900	0.52483400

C	-1.65130300	-3.22726100	0.04889900
C	-1.23380700	-0.64769700	-0.22497700
C	-0.05997100	0.17097300	0.25576500
C	0.82152600	-0.67583700	0.86386200
H	1.85614200	-3.28025100	1.77761500
H	0.55948600	-5.37204200	1.50193900
H	-1.64618200	-5.35429600	0.38565400
H	-2.60371500	-3.22696900	-0.47458500
P	2.38880500	0.05506200	1.31969100
P	-2.71059100	0.28414400	0.37116700
Pd	0.08418800	2.11357300	0.00042700
H	-1.28713200	-0.58368200	-1.33313300
H	-0.59547900	0.39605600	-3.19504800
C	-4.21818600	-0.30649700	-0.46928700
C	-5.42295700	-0.44596300	0.23069200
C	-4.16572800	-0.55159500	-1.85008700
C	-6.57363100	-0.84177900	-0.44750400
H	-5.46396400	-0.24706700	1.29698900
C	-5.32348800	-0.95115300	-2.51312500
H	-3.23966400	-0.42992000	-2.41058700
C	-6.52349300	-1.09723200	-1.81686400
H	-7.50803900	-0.94897000	0.09579200
H	-5.28445800	-1.14504400	-3.58091900
H	-7.42177600	-1.40748300	-2.34346500
C	-2.92457300	0.01891500	2.17371800
C	-2.79863100	1.10330500	3.04695700
C	-3.20529100	-1.25949700	2.67780100
C	-2.95273900	0.91130000	4.41879900
H	-2.58212600	2.08794600	2.64239200
C	-3.35598000	-1.44235100	4.05002600
H	-3.30186400	-2.10787300	2.00825800
C	-3.23048900	-0.35881900	4.92012300
H	-2.85560600	1.75662500	5.09384700
H	-3.57204300	-2.43339100	4.43858900
H	-3.35185300	-0.50623700	5.98977600
C	3.75709500	-1.00436600	0.74130500
C	4.80957300	-1.36995200	1.58895600
C	3.73507500	-1.42386000	-0.59714800
C	5.84588300	-2.16033400	1.09601600
H	4.82245700	-1.04206600	2.62401100
C	4.77765300	-2.21423400	-1.07315500
H	2.92434600	-1.14769500	-1.27157900
C	5.82952200	-2.58126000	-0.23295700
H	6.66478100	-2.44429100	1.75088200
H	4.76151700	-2.53849900	-2.10912500
H	6.63967700	-3.19633400	-0.61529100
C	2.53535400	0.19821700	3.14261100
C	3.28007400	1.24502100	3.69750700
C	1.91172400	-0.73775600	3.97608000
C	3.41351900	1.34294900	5.08083300
H	3.73382000	1.98800500	3.04734200
C	2.04549600	-0.62925600	5.35896100
H	1.31424900	-1.53963500	3.55251100
C	2.79821200	0.40651400	5.91125500
H	3.99193800	2.15647900	5.50923500
H	1.55537000	-1.35257100	6.00432000
H	2.89902600	0.48857900	6.98990600
C	0.29680000	4.35513100	0.30259700
C	0.24672200	4.31979600	-0.92422000
C	0.19746300	4.48341500	-2.37738300
C	1.28741100	3.72601400	-3.14650900
H	0.27051200	5.56239200	-2.56874300
H	-0.78092700	4.14043300	-2.73032000
C	1.02235800	2.22011400	-3.25525900
H	2.27630300	3.88151400	-2.70527500
H	1.33783400	4.12294600	-4.16709900
O	-0.12195700	1.82096000	-2.88190700
O	1.94639000	1.51362200	-3.71680400
H	0.38761300	4.61954100	1.33439300
C	0.59820000	-3.74401900	-5.15505800
C	1.38942800	-2.85428100	-4.43023300
C	0.84751600	-1.72355000	-3.81305200
C	-0.53986000	-1.48053100	-3.94316800
C	-1.32319000	-2.37112700	-4.68219600
C	-0.76678300	-3.49866100	-5.28311800
H	1.04907900	-4.61629000	-5.62083600
H	2.45729200	-3.02009600	-4.31591300
H	-1.39918100	-4.17609500	-5.85103300
H	-2.38465600	-2.15588900	-4.77322900
O	-1.16460800	-0.44118000	-3.32864900
O	1.64568900	-0.93328300	-3.05213300
H	1.70635900	0.02912500	-3.38277300

INT-3B
H= *-2935.19391* a.u.
G= *-2935.333918* a.u.

S	3.75827900	0.03144600	1.03608100
S	0.51164400	-0.50185800	-2.33389400
C	-0.50502800	-2.93067200	1.30270400
C	0.19559200	-2.36059800	2.39137300
C	-0.11757700	-2.73128400	3.69903000
C	-1.12579400	-3.67265900	3.90727900
C	-1.80758700	-4.24552900	2.83212700
C	-1.49586200	-3.88028700	1.51983200
C	0.06768800	-2.38022100	0.02608800
C	1.14877400	-1.41120600	0.47649900
C	1.19632500	-1.44124400	1.84017300
H	0.41613200	-2.30731600	4.54351700
H	-1.37955400	-3.96784000	4.92155100
H	-2.58617200	-4.98005300	3.01565500
H	-2.03362500	-4.32672900	0.68783300
P	2.48720800	-0.43706800	2.58540300
P	-0.93562800	-1.31924400	-1.13229500
Pd	2.19595900	-0.25958300	-0.72891400
H	0.50268200	-3.14873900	-0.62706100
C	-2.07644000	-2.28697300	-2.16851700
C	-2.95056400	-1.55548300	-2.98697400
C	-2.04993400	-3.68469900	-2.21936500
C	-3.80843900	-2.24393400	-3.84123300
H	-2.93913600	-0.46468500	-2.98190000
C	-2.92033700	-4.35880600	-3.07319000
H	-1.35334600	-4.25168100	-1.60863000
C	-3.80037900	-3.63836400	-3.87984000
H	-4.48295300	-1.68344900	-4.48136700
H	-2.90622900	-5.44420800	-3.11091600
H	-4.47662200	-4.16569800	-4.54685800
C	-1.94093300	-0.16717900	-0.14434200
C	-1.73147100	1.21051300	-0.22646900
C	-2.97377600	-0.69409700	0.64933900
C	-2.56091800	2.06595000	0.50341100
H	-0.96488400	1.63633600	-0.87142400
C	-3.77962500	0.16860200	1.38266400
H	-3.15508200	-1.76335400	0.68965700
C	-3.57412300	1.54854300	1.30591000
H	-2.43189500	3.13723400	0.38505300
H	-4.57998900	-0.23434700	1.99665400

H	-4.22683900	2.22188100	1.85531000
C	3.37353700	-1.38139600	3.88235500
C	3.01043000	-1.27697600	5.23140000
C	4.39440200	-2.25862300	3.49739800
C	3.66409000	-2.05297000	6.18642400
H	2.23051900	-0.58596600	5.53733800
C	5.04151400	-3.03111700	4.45838500
H	4.68538200	-2.31971300	2.45267200
C	4.67723500	-2.92980800	5.80075500
H	3.38431900	-1.96705900	7.23236500
H	5.83562600	-3.70824500	4.15791800
H	5.18734200	-3.53079700	6.54818600
C	1.76714000	1.03946000	3.38010000
C	2.58015800	1.86972400	4.16528800
C	0.43721100	1.39025100	3.12604100
C	2.05310300	3.03892000	4.70370000
H	3.61900300	1.60920100	4.34846200
C	-0.08024400	2.56578500	3.66804900
H	-0.18838300	0.76369400	2.49835800
C	0.72368400	3.38663700	4.45551100
H	2.68212800	3.68373000	5.31042400
H	-1.10979200	2.83854900	3.45940200
H	0.31821400	4.30465500	4.87116000
C	3.69808200	0.38375300	-2.28914800
C	3.24637000	1.50453900	-2.06797200
C	2.83978100	2.90342300	-1.96203100
C	1.99368000	3.37701400	-3.15741400
H	3.76231400	3.49018400	-1.86488800
H	2.24491900	3.06358600	-1.05519300
H	2.33885200	2.95041800	-4.10176400
H	2.09798000	4.46631100	-3.22980400
O	0.05249000	3.23198900	-1.78785300
O	-0.16489400	2.78558000	-3.98460900
H	4.23897400	-0.46131200	-2.66080500
H	-1.57489100	2.25638400	-3.57046600
C	0.49688200	3.09016100	-2.96394600
C	-5.73957100	4.38446800	-3.14145000
C	-4.56446600	4.73312200	-2.48015900
C	-3.43217900	3.91082700	-2.51342600
C	-3.48902300	2.70227000	-3.24991800
C	-4.67461600	2.37222400	-3.91112700
C	-5.80045500	3.19287900	-3.86034800
H	-6.60137900	5.04533600	-3.09292400
H	-4.49309600	5.65567000	-1.91020400
H	-6.70750200	2.90712000	-4.38646700
H	-4.68405400	1.44543300	-4.47957700
O	-2.45155700	1.82351800	-3.28577700
O	-2.35447200	4.32048600	-1.81138100
H	-1.54394900	3.75095300	-1.90950700

TS$_{3C}$

H= *-2935.179388* a.u.

G= *-2935.320713* a.u.

S	4.21750000	-0.45834000	-0.05010100
S	0.72767200	-1.64674200	-3.01521900
C	0.09364100	-3.45453800	1.05242100
C	0.95495500	-2.82847500	1.98345100
C	0.79511900	-3.06510300	3.34893000
C	-0.22184600	-3.92491300	3.76760800
C	-1.06123900	-4.55264000	2.84497100
C	-0.90071500	-4.32784200	1.47418100
C	0.53053000	-3.05836100	-0.32948000
C	1.68128300	-2.09443000	-0.12569100
C	1.90298600	-2.00670100	1.22186800
H	1.45150200	-2.60125800	4.07861900
H	-0.35639600	-4.11456900	4.82888400
H	-1.84078500	-5.22374400	3.19327100
H	-1.54768300	-4.82376000	0.75551000
P	3.26011100	-0.93894500	1.70693100
P	-0.57486300	-2.17145600	-1.53766700
Pd	2.44761600	-0.93265700	-1.53409100
H	0.86084800	-3.92229500	-0.92359500
C	-1.84565600	-3.33281300	-2.14851400
C	-3.20822600	-3.08404300	-1.95054900
C	-1.43359400	-4.48152400	-2.83939100
C	-4.15230400	-3.98915000	-2.43307500
H	-3.53098100	-2.18623500	-1.43352000
C	-2.38297600	-5.38019900	-3.31465400
H	-0.37811900	-4.65846000	-3.02870200
C	-3.74196200	-5.13545700	-3.11013400
H	-5.20962900	-3.79068300	-2.28515300
H	-2.06245100	-6.26632700	-3.85434800
H	-4.48111200	-5.83571100	-3.48846900
C	-1.39575100	-0.73348800	-0.77319600
C	-1.41564000	0.47138200	-1.48727300
C	-2.02610300	-0.83133000	0.47783600
C	-2.06453000	1.58148200	-0.94798500
H	-0.93840000	0.56710800	-2.45861900
C	-2.66888400	0.28659000	1.00596300
H	-2.01620800	-1.76308600	1.03482400
C	-2.68784700	1.48927700	0.29513300
H	-2.06527200	2.50175000	-1.52385600
H	-3.16071000	0.21392900	1.97214700
H	-3.19665400	2.35464000	0.71126700
C	4.40510300	-1.79733600	2.85201600
C	4.27739400	-1.66739400	4.24093600
C	5.38547800	-2.64026000	2.31420600
C	5.12524700	-2.38244600	5.08424400
H	3.53090600	-1.00163200	4.66377800
C	6.22903600	-3.35026500	3.16457700
H	5.49179600	-2.72385600	1.23630700
C	6.09948400	-3.22299500	4.54739400
H	5.02749900	-2.27682600	6.16079600
H	6.99208200	-3.99916500	2.74503500
H	6.76195800	-3.77553400	5.20757700
C	2.62479300	0.53164700	2.58690200
C	3.52515300	1.42398100	3.18637600
C	1.25436300	0.81222200	2.58386500
C	3.04930900	2.58363300	3.79007400
H	4.59191300	1.21750400	3.17578900
C	0.78733600	1.97763800	3.18994800
H	0.55667000	0.13347900	2.10321800
C	1.68088300	2.86044100	3.79269500
H	3.74715000	3.27547600	4.25246800
H	-0.27621300	2.19562200	3.18111600
H	1.31358700	3.76976800	4.25965200
C	3.07980900	0.36042000	-3.19022200
C	2.78302400	1.40423500	-2.57009900
C	2.69064800	2.44426600	-1.54329200
C	2.81402800	3.84425000	-2.15860200
H	3.47129500	2.25464300	-0.79768000
H	1.72040000	2.36070900	-1.03861600
H	3.80586700	3.97764200	-2.60673700
H	2.68010500	4.61497300	-1.39580700
O	1.56986900	2.88067100	-3.89785300
O	1.13579500	5.03486000	-3.40516100

H	3.13763100	-0.04441100	-4.18428300
H	0.10269100	4.89131900	-4.69658500
C	1.74858200	3.96181300	-3.25024100
C	-4.19510700	3.21895700	-5.66400700
C	-3.22053900	2.67287800	-4.82994600
C	-1.93252500	3.20863400	-4.76144900
C	-1.61134500	4.34533300	-5.54340000
C	-2.59822900	4.86967400	-6.38634800
C	-3.87631800	4.31962000	-6.45562800
H	-5.18767300	2.77729800	-5.69986500
H	-3.43311800	1.79842900	-4.21972700
H	-4.61768700	4.75723500	-7.11929200
H	-2.32533500	5.73634700	-6.98206300
O	-0.39433600	4.92250000	-5.56299100
O	-1.04805200	2.61641000	-3.91527000
H	-0.09469800	2.79207200	-4.13694600

INT-3C
H= *-2935.22738* a.u.
G= *-2935.37377* a.u.

S	2.03947000	0.49933600	0.49606700
S	-1.77045700	-1.56283300	-1.37224700
C	-0.55990900	-3.61026500	2.49932400
C	0.37923900	-2.79415300	3.17694500
C	0.72844100	-3.09083100	4.49525700
C	0.14274500	-4.19500700	5.11788900
C	-0.76955100	-5.00633700	4.44068400
C	-1.11896200	-4.72177300	3.11636700
C	-0.69241600	-3.08962700	1.09534800
C	0.17872000	-1.85039400	1.02901800
C	0.79669900	-1.73881800	2.24579100
H	1.44892000	-2.48180400	5.03247000
H	0.40937200	-4.43129500	6.14454400
H	-1.20483900	-5.86610100	4.94171900
H	-1.81919300	-5.35981300	2.58275700
P	1.97934600	-0.39370400	2.35301300
P	-2.29491600	-2.49204000	0.36848200
Pd	0.19533400	-0.55476600	-0.51232400
H	-0.35375900	-3.81933800	0.34558900
C	-3.39428400	-3.92155600	0.05049600
C	-4.66536500	-4.02950100	0.62499500
C	-2.93586300	-4.91884700	-0.82246700
C	-5.46477500	-5.13503600	0.33701400
H	-5.03441300	-3.25354000	1.28765400
C	-3.73805900	-6.02041500	-1.10181600
H	-1.96580800	-4.82122300	-1.30265600
C	-5.00209100	-6.13065100	-0.52038100
H	-6.45278000	-5.21263300	0.78140400
H	-3.37982200	-6.78863600	-1.78068200
H	-5.62864100	-6.98949900	-0.74349800
C	-3.12736200	-1.38490800	1.56850500
C	-3.49564400	-0.10083000	1.15423700
C	-3.40208100	-1.80690100	2.87795400
C	-4.14141500	0.75587200	2.04360500
H	-3.26535000	0.21895800	0.14196600
C	-4.04561100	-0.94141800	3.76047100
H	-3.11241000	-2.79767000	3.21288700
C	-4.41692700	0.33755400	3.34419900
H	-4.42652100	1.75177400	1.71753300
H	-4.25737300	-1.26995700	4.77403600
H	-4.92206500	1.00719100	4.03483100
C	3.63633100	-1.03655500	2.82309900
C	4.03234400	-1.12929200	4.16318800
C	4.48490200	-1.49896900	1.81060000
C	5.26739300	-1.68846400	4.48531600
H	3.38843800	-0.75305900	4.95277700
C	5.71762500	-2.05674200	2.14044200
H	4.18024700	-1.40380500	0.77208200
C	6.10896400	-2.15316200	3.47555000
H	5.57340100	-1.75515900	5.52547800
H	6.37538600	-2.41019100	1.35171900
H	7.07298300	-2.58519800	3.72913100
C	1.49764100	0.77973300	3.67460500
C	2.37818900	1.79974400	4.06104200
C	0.22104000	0.70633600	4.24173400
C	1.98364000	2.73110900	5.01672600
H	3.36620700	1.86786800	3.61403700
C	-0.16614800	1.64274200	5.19947400
H	-0.46736900	-0.07459700	3.93329800
C	0.71236200	2.65216400	5.58786100
H	2.66754400	3.52095500	5.31376500
H	-1.15748600	1.58195400	5.63906100
H	0.40727200	3.38111600	6.33341100
C	0.25558100	0.73256800	-2.10887300
C	-0.00710100	2.03405500	-2.10825500
C	-0.41630600	2.99134200	-1.01672100
C	0.01856300	4.34797900	-1.57093200
H	0.04533600	2.72431700	-0.06468200
H	-1.50597600	2.96871400	-0.88253900
H	1.03308300	4.61754400	-1.25180100
H	-0.63616600	5.19017100	-1.33954200
O	0.07092600	2.79772000	-3.31928700
O	0.11221500	4.94877400	-3.94367600
H	0.52211800	0.30637300	-3.07998200
H	0.00644900	4.63804600	-5.67271200
C	0.06515100	4.10746700	-3.06461900
C	-1.59426700	7.81658100	-8.66704800
C	-1.30976600	6.61767600	-9.32751400
C	-0.80928800	5.53631300	-8.61375500
C	-0.58791100	5.64734700	-7.22648200
C	-0.87285200	6.84235000	-6.57272400
C	-1.37739900	7.92758100	-7.29693800
H	-1.98545700	8.65917100	-9.23043500
H	-1.47148600	6.50579800	-10.39549800
H	-1.59722900	8.85799700	-6.78058400
H	-0.69712500	6.91515600	-5.50330100
O	-0.09382000	4.52462200	-6.64330400
O	-0.52687500	4.35979800	-9.23413800
H	-0.20345400	3.76808800	-8.53752400

INT-3D
H= *-2935.224824* a.u.
G= *-2935.367267* a.u.

S	2.44504900	1.16876800	0.39554600
S	-2.19917100	1.58071800	-0.37374300
C	-0.99649200	-2.76538000	0.10532200
C	0.29372300	-2.81771000	0.68748400
C	0.80044800	-4.03498100	1.14563600
C	0.01561800	-5.18293000	1.01935800
C	-1.25077200	-5.13002200	0.43385000
C	-1.76075800	-3.91599600	-0.03831600
C	-1.23636200	-1.35522700	-0.35364000
C	-0.02424900	-0.56259200	0.09146000
C	0.84210400	-1.45768200	0.66015100
H	1.79294100	-4.09713000	1.58248700
H	0.40232200	-6.13472000	1.37368300

H	-1.83997100	-6.03735300	0.33658900
H	-2.73879800	-3.87758200	-0.51169900
P	2.43203800	-0.76344800	1.10228900
P	-2.63862100	-0.31818100	0.25957200
C	3.77700700	-1.74978000	0.33523100
C	4.55174300	-2.65002400	1.07551700
C	3.97785100	-1.61993300	-1.04504300
C	5.52286200	-3.41811500	0.43452700
H	4.40550300	-2.74653100	2.14717800
C	4.94703400	-2.39342000	-1.67676400
H	3.38709000	-0.90981200	-1.61701700
C	5.71964900	-3.29133100	-0.93934300
H	6.12726000	-4.11231800	1.01153800
H	5.09819900	-2.28981800	-2.74705700
H	6.47845900	-3.88997800	-1.43568000
C	2.70780900	-0.83493200	2.91189400
C	3.85326800	-0.23745300	3.45542800
C	1.76718500	-1.43835000	3.75228800
C	4.05877800	-0.25796700	4.83126400
H	4.57306700	0.25383000	2.80631800
C	1.98093800	-1.45501400	5.13062000
H	0.86950700	-1.88439200	3.33544500
C	3.12384700	-0.86854900	5.66934000
H	4.94507200	0.20928900	5.25062800
H	1.24814100	-1.92281300	5.78176400
H	3.28534700	-0.87964100	6.74364600
C	-4.21435000	-0.90841500	-0.45519800
C	-4.27087700	-1.15457100	-1.83536400
C	-5.36058000	-1.06532200	0.33206400
C	-5.46725000	-1.56977600	-2.41271800
H	-3.39457500	-1.00826000	-2.46184200
C	-6.55455400	-1.47809700	-0.25693500
H	-5.32329900	-0.86546600	1.39815400
C	-6.60792500	-1.73362100	-1.62552700
H	-5.50856800	-1.76031200	-3.48108900
H	-7.44287800	-1.59711600	0.35657300
H	-7.53983700	-2.05559000	-2.08161700
C	-2.74380800	-0.45757700	2.08213000
C	-2.55727900	0.68409500	2.86817100
C	-2.99054000	-1.69828900	2.68829000
C	-2.62283100	0.58523800	4.25698900
H	-2.35321300	1.63741900	2.38887400
C	-3.05291100	-1.78635200	4.07690000
H	-3.12694400	-2.59098800	2.08621900
C	-2.87115700	-0.64605600	4.86051600
H	-2.47572300	1.47334400	4.86435900
H	-3.24391600	-2.74755900	4.54550800
H	-2.92330800	-0.71914600	5.94337800
Pd	0.14457300	1.43844100	-0.05252200
H	-1.32731300	-1.26861000	-1.44843600
C	0.45915900	3.47652300	-0.14645000
C	-0.18768200	4.48736400	-0.73596100
H	1.31823200	3.81921000	0.43724900
O	0.27607600	5.81011900	-0.55221700
C	-1.41213800	4.56106800	-1.62022000
C	-0.31707000	6.67909900	-1.41239300
C	-1.25020400	5.90579200	-2.32581300
H	-2.31766300	4.56104300	-1.00003500
H	-1.47401500	3.71346400	-2.30386600
O	-0.07762100	7.85887100	-1.40693900
H	-2.17316300	6.46569200	-2.48767100
H	-0.75097300	5.80115000	-3.29670600
H	0.62779500	2.35198900	-2.30775900
C	1.93179300	-1.26002000	-4.94157200
C	0.60697700	-1.25047500	-4.50350000
C	0.09398900	-0.10846300	-3.89490600
C	0.90245500	1.02978000	-3.73508300
C	2.22165600	1.01257300	-4.17270200
C	2.74131200	-0.13735400	-4.77281200
H	2.32830500	-2.15150700	-5.41949600
H	-0.03444500	-2.11825000	-4.62476700
H	3.76980000	-0.14472000	-5.12198000
H	2.82701200	1.90559300	-4.04694700
O	0.29106600	2.13988800	-3.20419600
O	-1.19053400	-0.07935800	-3.44309800
H	-1.30346700	0.78879600	-3.01299500

TS$_{3E}$

H= *-2935.209124* a.u.

G= *-2935.347963* a.u.

S	2.76163800	1.40521400	-0.60048800
S	-1.86928800	1.80662500	-1.47148400
C	-0.70485900	-2.47237500	-0.70698400
C	0.56072000	-2.49872000	-0.07461800
C	1.04371300	-3.68938200	0.46933400
C	0.25657300	-4.83883400	0.37895800
C	-0.98760900	-4.81214200	-0.25412100
C	-1.47264100	-3.62489100	-0.81184900
C	-0.92024500	-1.09129400	-1.25296100
C	0.28211200	-0.28619800	-0.80277000
C	1.12797500	-1.14896500	-0.15823900
H	2.01836200	-3.72983900	0.94674300
H	0.62382300	-5.77117200	0.79923000
H	-1.57802800	-5.72117100	-0.32384300
H	-2.43035900	-3.60968900	-1.32605600
P	2.76455100	-0.50326400	0.15860800
P	-2.36036000	-0.04678200	-0.76731800
C	3.99946900	-1.55127500	-0.69911100
C	4.78198000	-2.48678600	-0.01211700
C	4.09975200	-1.43120400	-2.09151100
C	5.66646800	-3.29892000	-0.71999500
H	4.71044300	-2.57675900	1.06774600
C	4.98181400	-2.25162300	-2.78862600
H	3.50403700	-0.69819500	-2.62940100
C	5.76540500	-3.18215400	-2.10567700
H	6.27916500	-4.02028600	-0.18666200
H	5.04938300	-2.15429200	-3.86761800
H	6.45667500	-3.81625100	-2.65406200
C	3.17312000	-0.53647400	1.94097500
C	4.41714400	-0.05721900	2.37401300
C	2.22974200	-0.98068300	2.87297600
C	4.71414100	-0.03287600	3.73305000
H	5.14562000	0.30383200	1.65302100
C	2.53646100	-0.95480200	4.23315600
H	1.26089600	-1.33937200	2.53867600
C	3.77500900	-0.48299800	4.66271500
H	5.67694700	0.34236900	4.06748400
H	1.80328300	-1.30085200	4.95601100
H	4.00941200	-0.46070100	5.72328600
C	-3.87945100	-0.67810700	-1.55969000
C	-3.83188700	-0.98952900	-2.92750600
C	-5.07988300	-0.80002100	-0.85080500
C	-4.98330000	-1.43789900	-3.56827400
H	-2.91301700	-0.86651500	-3.49673900
C	-6.22692400	-1.24627800	-1.50483200
H	-5.12131400	-0.54667800	0.20376100

C	-6.17835500	-1.56890100	-2.85954800
H	-4.94575700	-1.67955700	-4.62634000
H	-7.15807700	-1.33884100	-0.95340700
H	-7.07377900	-1.91735100	-3.36650300
C	-2.57028200	-0.11157100	1.05043600
C	-2.35359600	1.04587600	1.80443500
C	-2.91071100	-1.31394500	1.68787600
C	-2.48327300	1.00281800	3.19145600
H	-2.07795800	1.96744900	1.29979700
C	-3.03761300	-1.34685300	3.07431100
H	-3.07109100	-2.21955700	1.11150800
C	-2.82601800	-0.19000800	3.82541100
H	-2.31345000	1.90336500	3.77418200
H	-3.30111800	-2.27831700	3.56701500
H	-2.92835300	-0.22026800	4.90668800
Pd	0.50967500	1.61028100	-1.26195300
H	-0.94318300	-1.05264100	-2.35728000
C	1.04279500	3.63587700	-1.99287000
C	0.22961400	4.71442700	-1.97420800
H	2.06169100	3.86698700	-1.68132000
O	0.68102900	5.93395500	-1.51334300
C	-1.18073800	4.87715000	-2.47928800
C	-0.18525500	6.95694700	-1.84187200
C	-1.30273500	6.38486700	-2.68918900
H	-1.88779300	4.53371500	-1.71412700
H	-1.33991500	4.27200500	-3.37341100
O	0.00995300	8.08454100	-1.48832200
H	-2.25870600	6.82229400	-2.39651000
H	-1.11052200	6.67484800	-3.72886400
H	0.83458000	2.92271000	-3.03882300
C	2.54122000	-0.94742400	-5.70731300
C	1.21145200	-1.07229100	-5.27699900
C	0.56171300	0.03597600	-4.76192100
C	1.19473600	1.30745500	-4.66310400
C	2.52545300	1.40173900	-5.09165300
C	3.18914500	0.28193100	-5.60845700
H	3.05276800	-1.80874900	-6.12885900
H	0.68295400	-2.01962600	-5.34798000
H	4.21725700	0.38281100	-5.94869100
H	3.02567000	2.36453000	-5.02824800
O	0.45142200	2.29644700	-4.18312400
O	-0.73384000	-0.00940600	-4.31683500
H	-0.87531700	0.92378200	-4.03264900

INT-3E

H= *-2935.223641* a.u.

G= *-2935.363479* a.u.

S	2.40351800	1.43964800	0.25388500
S	-2.35519500	1.86576400	0.08940800
C	-1.04260100	-2.42668600	-0.05009300
C	0.26839100	-2.50367700	0.47418300
C	0.82468400	-3.74377700	0.79091700
C	0.06016600	-4.89391100	0.58738200
C	-1.23082300	-4.81634600	0.06083800
C	-1.78768100	-3.57782100	-0.27257300
C	-1.34274500	-0.98939000	-0.35420000
C	-0.10991400	-0.22685300	0.07507400
C	0.79649800	-1.13773400	0.54355600
H	1.83904200	-3.82099500	1.17183100
H	0.48303700	-5.86521800	0.82902900
H	-1.80208100	-5.72536100	-0.10407400
H	-2.78285900	-3.51972600	-0.70578400
P	2.40721800	-0.48448800	0.95755000
P	-2.72011400	-0.09909300	0.49648400
C	3.69545800	-1.50208200	0.15381900
C	4.69245600	-2.15742400	0.88524700
C	3.63663800	-1.62948300	-1.24129300
C	5.63227000	-2.94203600	0.21834800
H	4.73762700	-2.05825000	1.96547200
C	4.57761600	-2.41987600	-1.89400300
H	2.87412500	-1.11210200	-1.82068100
C	5.57352800	-3.07464400	-1.16801100
H	6.40881500	-3.44869800	0.78439700
H	4.52305500	-2.52067400	-2.97361200
H	6.30589100	-3.68922100	-1.68439900
C	2.69925500	-0.56258000	2.76520700
C	3.70147400	0.23351300	3.33373600
C	1.92665900	-1.40251700	3.57412500
C	3.93822500	0.17459700	4.70437800
H	4.27870300	0.90834500	2.70749600
C	2.16692700	-1.45170300	4.94683200
H	1.13580400	-2.00641500	3.13978900
C	3.17216300	-0.66843400	5.51076300
H	4.71483700	0.79367100	5.14420300
H	1.56207900	-2.09960500	5.57463800
H	3.35512100	-0.70789800	6.58097500
C	-4.32362600	-0.64591600	-0.18344100
C	-4.45707000	-0.76632000	-1.57546100
C	-5.41939000	-0.88393500	0.65497800
C	-5.68643100	-1.13946200	-2.11253900
H	-3.61926900	-0.56047600	-2.23936100
C	-6.64461700	-1.25202800	0.10329100
H	-5.31916800	-0.78242500	1.73097100
C	-6.77761400	-1.38295000	-1.27788300
H	-5.78986800	-1.23350600	-3.18947500
H	-7.49433400	-1.43431600	0.75484500
H	-7.73410400	-1.67030800	-1.70558500
C	-2.70053000	-0.49021400	2.28798800
C	-2.48907200	0.53853300	3.21085800
C	-2.89302900	-1.80568800	2.73476600
C	-2.47068700	0.25409700	4.57512600
H	-2.33891000	1.55249700	2.85129600
C	-2.87079600	-2.08101100	4.09990100
H	-3.05495700	-2.61121800	2.02593700
C	-2.66133800	-1.05285000	5.01963400
H	-2.30733100	1.05638100	5.28876100
H	-3.01954800	-3.10059800	4.44381400
H	-2.64937700	-1.27189900	6.08385700
Pd	0.04073100	1.72771900	0.00756900
H	-1.53984200	-0.78952500	-1.42487200
C	0.27580200	3.94786100	0.58530700
C	0.06590900	4.26737900	-0.72488400
H	-0.52915000	4.07723500	1.30104800
O	-1.17832800	4.59777500	-1.14732500
C	1.07090100	4.46885700	-1.83331000
C	-1.18429500	4.90568300	-2.52010100
C	0.19193900	4.65359300	-3.06588700
H	1.72414800	3.60107300	-1.92985200
H	1.67256900	5.35639800	-1.60248400
O	-2.18662500	5.28803700	-3.04232500
H	0.13994300	3.69241900	-3.59638800
H	0.48679900	5.45413600	-3.74592300
H	1.29151500	3.92350600	0.96292400
C	1.15194200	-1.66888500	-4.82800600
C	-0.16458200	-1.42477100	-4.39956200
C	-0.43690000	-0.26149300	-3.70770000

C	0.56084700	0.71480600	-3.39430800
C	1.86811600	0.43873400	-3.83813400
C	2.14838500	-0.73635200	-4.55144200
H	1.38002700	-2.57390500	-5.38430800
H	-0.96675200	-2.12552800	-4.61869500
H	3.16297900	-0.91133900	-4.90590000
H	2.65323000	1.16302400	-3.63329900
O	0.14793100	1.77190200	-2.74351700
O	-1.68704500	0.08908800	-3.25693100
H	-1.47366000	0.99254800	-2.89149700

TS_{3F}

H= *-2935.215853* a.u.

G= *-2935.354378* a.u.

S	2.63409200	1.72032800	-1.32000200
S	-2.09353100	1.96768100	-1.88007700
C	-0.70665800	-2.21324700	-1.03771900
C	0.56771800	-2.15621000	-0.42456700
C	1.12913600	-3.30757100	0.13010000
C	0.40308500	-4.49873600	0.08500800
C	-0.85439400	-4.55244300	-0.52032100
C	-1.41216800	-3.40903600	-1.09931100
C	-1.00301000	-0.86893500	-1.62787800
C	0.16633700	-0.00163300	-1.23868700
C	1.06279100	-0.78439800	-0.56481500
H	2.12303300	-3.28997700	0.56869200
H	0.83104200	-5.40104500	0.51312400
H	-1.39515900	-5.49373100	-0.55832400
H	-2.37457000	-3.45923700	-1.60201000
P	2.64170200	-0.01656700	-0.23138300
P	-2.49458600	0.09443800	-1.15872200
C	3.97848700	-1.13946800	-0.76623900
C	4.96811300	-1.58969500	0.11468300
C	3.97498300	-1.55184800	-2.10630900
C	5.95368200	-2.46086100	-0.34702400
H	4.97289500	-1.26267700	1.15012500
C	4.96188300	-2.42474300	-2.55293000
H	3.22526100	-1.18850900	-2.80583700
C	5.94843700	-2.87954600	-1.67678100
H	6.72550500	-2.80913000	0.33351000
H	4.94928200	-2.74234500	-3.59090300
H	6.71780200	-3.55951700	-2.03244600
C	2.85857900	0.29739300	1.56219800
C	3.58190100	1.41916800	1.98234700
C	2.30205100	-0.57541300	2.50541200
C	3.76195400	1.65499200	3.34382400
H	3.97766300	2.11529400	1.24802300
C	2.48485500	-0.33023500	3.86486700
H	1.71785400	-1.43289000	2.18437200
C	3.21683900	0.78070500	4.28371800
H	4.31983500	2.52889000	3.66718300
H	2.04865900	-1.00457000	4.59634900
H	3.35475000	0.97014300	5.34466700
C	-3.97988300	-0.62736200	-1.93385600
C	-3.90445300	-1.04881800	-3.27119800
C	-5.18654100	-0.71189500	-1.22809500
C	-5.04457300	-1.56584600	-3.88217600
H	-2.97538900	-0.96994700	-3.83669400
C	-6.31827600	-1.22716900	-1.85568700
H	-5.24352700	-0.37929300	-0.19632100
C	-6.24673400	-1.65621900	-3.18016600
H	-4.98899900	-1.89510900	-4.91560100
H	-7.25434300	-1.29213400	-1.30853200
H	-7.13047000	-2.05899300	-3.66737800
C	-2.73804300	0.06811200	0.65968500
C	-2.73157200	1.26783100	1.37691800
C	-2.93152100	-1.14795000	1.33123500
C	-2.91568100	1.25404300	2.75832600
H	-2.58286200	2.20222700	0.84372400
C	-3.11021700	-1.15313700	2.71241100
H	-2.93672700	-2.08576400	0.78550600
C	-3.10304800	0.04545300	3.42615700
H	-2.91292800	2.18895900	3.31135800
H	-3.25566700	-2.09676900	3.23024600
H	-3.24570600	0.03610400	4.50318400
Pd	0.31250200	1.88216400	-1.77674500
H	-1.05877600	-0.87550200	-2.75260400
C	0.43181100	3.89045900	-2.92880900
C	0.58880200	4.43655800	-1.69544500
H	1.28081800	3.70062200	-3.57498500
O	1.84343700	4.73138900	-1.22009700
C	-0.43134300	4.97722300	-0.72315200
C	1.79741300	5.06341700	0.12353500
C	0.35645200	4.98163500	0.58417000
H	-1.34642700	4.38540400	-0.70639700
H	-0.68559300	6.00194700	-1.02439300
O	2.78595900	5.34897700	0.73249400
H	0.24168600	4.03439500	1.12462200
H	0.12871900	5.79951700	1.26916700
H	-0.55044100	3.85836200	-3.38557800
C	2.07855200	-2.26549100	-5.65264800
C	0.72177900	-2.14204100	-5.29100300
C	0.25384700	-0.89649800	-4.91359800
C	1.11540300	0.24739000	-4.87253500
C	2.45291300	0.10969700	-5.23048900
C	2.92198800	-1.15875000	-5.62640400
H	2.46048900	-3.23346100	-5.96808100
H	0.05208300	-2.99780400	-5.32282700
H	3.96150200	-1.26615700	-5.92984800
H	3.11491000	0.97131300	-5.21591000
O	0.43857100	1.32517500	-4.48690900
O	-0.99120200	-0.52373800	-4.54376800
H	-0.62591600	0.59876700	-4.43238200

INT-3F

H= *-2935.252314* a.u.

G= *-2935.396041* a.u.

S	2.39365600	1.23119500	0.19829400
S	-2.20423000	1.42030300	-0.85786700
C	-1.13547300	-2.43916100	1.12573100
C	0.28115500	-2.55544000	1.33259100
C	0.81244500	-3.74733800	1.85085800
C	-0.04050900	-4.79865400	2.15645200
C	-1.42685200	-4.68597800	1.94951600
C	-1.97746600	-3.51964100	1.43679400
C	-1.37651300	-1.12512100	0.58869700
C	-0.14525500	-0.43310500	0.46504500
C	0.87329100	-1.30854800	0.91904600
H	1.88313500	-3.85336800	2.00586800
H	0.36811900	-5.72340400	2.55524200
H	-2.07430000	-5.52559200	2.18838700
H	-3.04958500	-3.45072100	1.26831700
P	2.50441300	-0.66607300	1.00901800
P	-2.83743500	-0.23931000	0.21218100
C	3.75805600	-1.66746800	0.10918400
C	4.47461000	-2.67617700	0.76502400

C	3.94104200	-1.46208700	-1.26271300
C	5.36407600	-3.47506100	0.04901400
H	4.34797800	-2.83239000	1.83227400
C	4.83326200	-2.26258200	-1.97153300
H	3.39410100	-0.67193200	-1.76863000
C	5.54440900	-3.26873200	-1.31792300
H	5.91890500	-4.25561900	0.56194100
H	4.97069400	-2.09444100	-3.03553000
H	6.24177000	-3.89024600	-1.87290300
C	3.11513900	-0.57760700	2.73716900
C	4.41589400	-0.12685700	2.99900300
C	2.25994000	-0.90257900	3.79433100
C	4.85513800	-0.00534500	4.31361100
H	5.07965000	0.13440800	2.17927800
C	2.70979600	-0.78397200	5.10921400
H	1.25287100	-1.24990800	3.58423200
C	4.00297600	-0.33556200	5.36905800
H	5.86153500	0.35015100	4.51537700
H	2.04617000	-1.04221600	5.92957600
H	4.34916600	-0.24024000	6.39454000
C	-4.03946000	-1.22250500	-0.76021200
C	-3.55954300	-1.91641600	-1.88012900
C	-5.39403300	-1.29178700	-0.41627600
C	-4.44102100	-2.67050300	-2.64903200
H	-2.50750900	-1.86374500	-2.14893900
C	-6.26770900	-2.05080100	-1.19341900
H	-5.76632800	-0.76228000	0.45502600
C	-5.79269300	-2.73849100	-2.30830300
H	-4.06871500	-3.20797800	-3.51635300
H	-7.31875100	-2.10463900	-0.92409800
H	-6.47544400	-3.33009300	-2.91208100
C	-3.73151000	0.31703900	1.72040300
C	-4.57915600	1.43075100	1.68035900
C	-3.55116000	-0.38318400	2.91817800
C	-5.25266800	1.83195900	2.83200700
H	-4.69723000	1.98897600	0.75546800
C	-4.22803600	0.02512600	4.06679300
H	-2.87726500	-1.23459900	2.95188800
C	-5.07855000	1.12884600	4.02463800
H	-5.90847300	2.69758100	2.79948400
H	-4.08400800	-0.51773900	4.99664000
H	-5.60122900	1.44639100	4.92268900
Pd	0.09562100	1.40450100	-0.25650200
H	0.05734500	-1.09791800	-1.80879300
C	0.44434900	3.31054900	-1.53519500
C	0.31048800	3.92205200	-0.32619400
H	1.43196600	3.15675600	-1.95659700
O	1.42203400	4.27112100	0.39621100
C	-0.90982600	4.50143700	0.34841500
C	1.07051100	4.73484600	1.65858000
C	-0.43499500	4.64587600	1.79149500
H	-1.79308400	3.87608800	0.21768100
H	-1.10935000	5.48572800	-0.09505900
O	1.89607200	5.11723500	2.43201900
H	-0.66299000	3.74905700	2.37989600
H	-0.82067200	5.51358800	2.32822700
H	-0.40349300	3.24016800	-2.20647800
C	2.31617700	-1.45372100	-5.24531300
C	1.51966700	-1.81154200	-4.15587600
C	0.52618400	-0.94870500	-3.70685100
C	0.30570400	0.27840700	-4.35544700
C	1.10050600	0.63138100	-5.44678700
C	2.10161100	-0.23315400	-5.88521500
H	3.08792800	-2.13131500	-5.59872700
H	1.65235400	-2.76140300	-3.64596200
H	2.71383500	0.04962900	-6.73716300
H	0.91756800	1.58161500	-5.93928600
O	-0.66903900	1.11601500	-3.93143300
O	-0.33349400	-1.28337300	-2.68034900
H	-1.10595300	0.70515700	-3.16286800

4-Pentynoic acid

H= *-344.297415* a.u.

G= *-344.338666* a.u.

C	-0.02735400	4.55077800	0.74272900
C	-0.04870600	4.56587000	-0.46546700
H	-0.00001100	4.54328900	1.80841800
C	-0.09003500	4.57299000	-1.92329200
H	-0.15701200	5.60353600	-2.29105100
H	0.84882800	4.17597600	-2.32657800
C	-1.26432600	3.75938400	-2.47534500
H	-1.21457700	2.71756300	-2.13930500
H	-2.22022100	4.14486400	-2.10327400
C	-1.29990700	3.77206300	-3.98270800
O	-0.51366400	4.34359600	-4.70389500
O	-2.33761800	3.05313100	-4.45874500
H	-2.28527200	3.11394100	-5.42550100

Catechol

H= *-382.432004* a.u.

G= *-382.470182* a.u.

C	0.34062600	0.72079800	0.12122200
C	1.71499900	0.67503200	-0.08658800
C	2.43331800	1.87549100	-0.21752300
C	1.78048500	3.09894500	-0.14065600
C	0.39931000	3.13802500	0.06815800
C	-0.31530200	1.95067000	0.19832800
H	-0.19792300	-0.21644300	0.21996500
H	2.34914300	4.02136000	-0.24349500
H	-0.10740300	4.09626700	0.12782500
H	-1.38866100	1.97467300	0.36097100
O	2.35846800	-0.51783900	-0.16229100
H	3.29586700	-0.32958400	-0.30808700
O	3.78150300	1.71950100	-0.41897400
H	4.19978300	2.58243100	-0.50370700

Product

H= *-344.344800* a.u.

G= *-344.38194* a.u.

O	1.19472300	-0.28100900	0.17837700
C	0.51836600	1.97379300	0.01445000
C	-0.66451800	1.06916300	-0.32710100
C	-0.16873900	-0.32418600	0.01640500
C	1.67949300	1.00813800	0.03782500
C	2.98511100	1.24021400	-0.04197500
H	3.35000300	2.25517500	-0.14486900
H	3.70309600	0.43014600	0.00334900
O	-0.78795600	-1.34409600	0.12358500
H	0.67984200	2.78680700	-0.69625600
H	0.38993300	2.42110100	1.00729000
H	-0.90215600	1.07872500	-1.39696800
H	-1.58624800	1.28328200	0.21623600

References

S1 N. Á. Espinosa-Jalapa, D. Ke, N. Nebra, L. Le Goanvic, S. Mallet-Ladeira, J. Monot, B. Martin-Vaca, D. Bourissou *ACS Catal.* **2014**, *4*, 3605-3611.

S2 Y. Matsuura, W. Chai, E. Endoh, J. Suhara, H. Hamada, C. A. Horiuchi *Phytochemistry* **2002**, *61*, 669-673.

S3 Gaussian 09, Revision D.02, M. J. Frisch, G. W. Trucks, H. B. Schlegel, G. E. Scuseria, M. A. Robb, J. R. Cheeseman, G. Scalmani, V. Barone, B. Mennucci, G. A. Petersson, H. Nakatsuji, M. Caricato, X. Li, H. P. Hratchian, A. F. Izmaylov, J. Bloino, G. Zheng, J. L. Sonnenberg, M. Hada, M. Ehara, K. Toyota, R. Fukuda, J. Hasegawa, M. Ishida, T. Nakajima, Y. Honda, O. Kitao, H. Nakai, T. Vreven, J. A. Montgomery, Jr., J. E. Peralta, F. Ogliaro, M. Bearpark, J. J. Heyd, E. Brothers, K. N. Kudin, V. N. Staroverov, R. Kobayashi, J. Normand, K. Raghavachari, A. Rendell, J. C. Burant, S. S. Iyengar, J. Tomasi, M. Cossi, N. Rega, J. M. Millam, M. Klene, J. E. Knox, J. B. Cross, V. Bakken, C. Adamo, J. Jaramillo, R. Gomperts, R. E. Stratmann, O. Yazyev, A. J. Austin, R. Cammi, C. Pomelli, J. W. Ochterski, R. L. Martin, K. Morokuma, V. G. Zakrzewski, G. A. Voth, P. Salvador, J. J. Dannenberg, S. Dapprich, A. D. Daniels, O. Farkas, J. B. Foresman, J. V. Ortiz, J. Cioslowski, D. J. Fox, Gaussian, Inc., Wallingford CT, **2009**.

S4 a) A. D. Becke, *J. Chem. Phys.* **1993**, *98*, 5648–5652; b) J. P. Perdew, Y. Wang, *Phys. Rev. B* **1992**, 45, 13244-13249.

S5 a) D. Andrae, U. Haüssermann, M. Dolg, H. Stoll, H. Preuss, *Theor. Chim. Acta*, **1990**, 77, 123–141; b)

S6 A. W. Ehlers, M. Böhme, S. Dapprich, A. Gobbi, A. Höllwarth, V. Jonas, K. F. Köhler, R. Stegmann, A. Veldkamp, G. Frenking, *Chem. Phys. Lett.* **1993**, *208*, 111–114.

S7 A. Bergner, M. Dolg, W. Küchle, H. Stoll, H. Preuss, *Mol. Phys.* **1993**, 80, 1431–1441.

S8 A. Höllwarth, M. Böhme, S. Dapprich, A. Ehlers, A. Gobbi, V. Jonas, K. F. Köhler, R. Stegmann, A. Veldkamp, G. Frenking, *Chem. Phys. Lett.* **1993**, 208, 237–240.

S9 P. C. Hariharan, J. A. Pople, *Theor. Chim. Acta* **1973**, *28*, 213–222.

S10 a) C. Gonzalez, H. B. Schlegel. *J. Chem. Phys.* **1989**, *90*, 2154–2161. b) C. Gonzalez, H. B. Schlegel *J. Phys. Chem.* **1990**, *94*, 5523–5527.

S11 C. Y. Legault, CYLview, 1.0b, Université de Sherbrooke, **2009**, http://www.cylview.org.
